# Association between SpO_2_ and the risk of death in elderly T2DM patients with cerebral infarction: a retrospective cohort study

**DOI:** 10.3389/fneur.2024.1344000

**Published:** 2024-03-12

**Authors:** Shuo Zhang, Jiaqi Ji, Siqi Gao, Shu Yang, Zeyi Song, Jianmin Li, Junjie Liu

**Affiliations:** ^1^College of Clinical Medicine, North China University of Science and Technology, Tangshan, China; ^2^School of Basic Medical Sciences, North China University of Science and Technology, Tangshan, China

**Keywords:** SpO_2_, 1-year mortality, elderly T2DM with cerebral infarction, Intensive Care Unit, MIMIC-IV database

## Abstract

**Objective:**

This study aimed to evaluate the SpO_2_ (transcutaneous oxygen saturation) -mortality link in elderly T2DM (diabetes mellitus type 2) patients with cerebral infarction and identify their optimal SpO_2_ range.

**Methods:**

In this investigation, we employed a comprehensive approach. Initially, we screened the MIMIC-IV database, identifying elderly T2DM patients with cerebral infarction, utilizing specific ICD-9 and ICD-10 codes. We then harnessed the power of restricted cubic splines to craft a visual representation of the correlation between SpO_2_ and 1-year mortality. To enhance our analysis, we harnessed Cox multivariate regression, allowing us to compute adjusted hazard ratios (HR) accompanied by 95% confidence intervals (CIs). Additionally, we crafted Cumulative Mortality Curve analyses, augmenting our study by engaging in rigorous subgroup analyses, stratifying our observations based on pertinent covariates.

**Results:**

In this study, 448 elderly T2DM patients with cerebral infarction were included. Within 1-year post-discharge, 161 patients (35.94%) succumbed. Employing Restricted Cubic Spline analysis, a statistically significant *U*-shaped non-linear relationship between admission ICU SpO_2_ levels and 1-year mortality was observed (*P*-value < 0.05). Further analysis indicated that both low and high SpO_2_ levels increased the mortality risk. Cox multivariate regression analysis, adjusting for potential confounding factors, confirmed the association of low (≤94.5%) and high SpO_2_ levels (96.5–98.5%) with elevated 1-year mortality risk, particularly notably high SpO_2_ levels (>98.5%) [HR = 2.06, 95% CI: 1.29–3.29, *P*-value = 0.002]. The cumulative mortality curves revealed the following SpO_2_ subgroups from high to low cumulative mortality at the 365th day: normal levels (94.5% < SpO_2_ ≤ 96.5%), low levels (SpO_2_ ≤ 94.5%), high levels (96.5% < SpO_2_ ≤ 98.5%), and notably high levels (>98.5%). Subgroup analysis demonstrated no significant interaction between SpO_2_ and grouping variables, including Sex, Age, Congestive heart failure, Temperature, and ICU length of stay (LOS-ICU; *P*-values for interaction were >0.05).

**Conclusions:**

Striking an optimal balance is paramount, as fixating solely on lower SpO_2_ limits or neglecting high SpO_2_ levels may contribute to increased mortality rates. To mitigate mortality risk in elderly T2DM patients with cerebral infarction, we recommend maintaining SpO_2_ levels within the range of 94.5–96.5%.

## 1 Introduction

Type 2 Diabetes Mellitus (T2DM) is a non-communicable disease, ranking third in terms of mortality rates after cancer and cardiovascular disease, which is more common in the middle-aged and elderly population, accounting for more than 90 % of all types of diabetes (1, 2). Based on the latest data of the International Diabetes Federation (IDF), the number of adults with diabetes in the world has exceeded 537 million, and the number of patients is still increasing. By 2045, it is anticipated that the figure will rise to 783 million, while the number of patients in China accounts for about 25% of its total number, ranking first in the world (3). Owing to shifts in lifestyle patterns and the aging population, the incidence of T2DM among the elderly in China has witnessed a substantial and ongoing increase. A close relationship exists between T2DM and cerebral infarction. T2DM is recognized as one of the independent risk factors for cerebral infarction. Furthermore, cerebral infarction represents a severe vascular complication of T2DM (4). Studies have shown that the risk of stroke in diabetic patients is 1.5–3.0 times that of non-diabetic patients. The elevated rates of disability and mortality associated with cerebral infarction in elderly individuals suffering from T2DM have emerged as a significant social problem (5, 6).

Oxygen is considered essential for human survival. In the Intensive Care Unit (ICU), oxygen therapy is one of the commonly used treatments. For the treatment or prevention of hypoxia, sufficient oxygen is often given (7). Peripheral Capillary Oxygen Saturation (SpO_2_) can be used to observe the patients' blood oxygen levels in real time and is typically measured using a pulse oxygen saturation meter, which is more convenient and non-invasive (8). In patients with brain injury, low levels of SpO_2_ may lead to brain tissue hypoxia, eventually leading to cell death. Recent studies have found that high levels of SpO_2_ can cause vasoconstriction in key systems such as cerebral arteries and coronary arteries, prompting the generation of oxygen free radicals, triggering oxidative stress and cellular damage (9). According to the study, a potential *U*-shaped correlation might exist between SpO_2_ and the risk of mortality (10). Therefore, it is crucial to maintain a moderate SpO_2_ range for elderly T2DM patients with cerebral infarction. Nevertheless, the specific range of SpO_2_ for optimal clinical effectiveness remains uncertain.

The aim of this study is to elucidate the relationship between SpO_2_ and 1-year mortality in elderly T2DM patients with cerebral infarction, and to use the MIMIC-IV database to determine the ideal range of SpO_2_ for elderly T2DM patients with cerebral infarction.

## 2 Materials and methods

### 2.1 Data source

We conducted a study involving elderly T2DM patients with cerebral infarction. The data for this study was obtained from the MIMIC-IV (Medical Information Mart for Intensive Care IV, version 1.0) database, which was maintained by the Massachusetts Institute of Technology (MIT). This database contained records of more than 70,000 adult patients who were admitted to the Intensive Care Unit (ICU) at Beth Israel Deaconess Medical Center in Boston between 2008 and 2019. As the data utilized in this study was sourced from the publicly available MIMIC-IV database, the requirement for informed consent was waived. One of the authors, SZ, was granted full access to the MIMIC-IV public database and was responsible for extracting the necessary data. The certification number for this data extraction work was 57262685. Our research adhered to the guidelines outlined in the Statement for Strengthening Reports of Observational Studies in Epidemiology (STROBE).

### 2.2 Participants

A comprehensive cohort comprising 2,197 elderly T2DM patients with cerebral infarction, who were admitted to the Intensive Care Unit (ICU) for the initial occurrence, was meticulously assembled from the MIMIC-IV public database. Following stringent inclusion and exclusion criteria, the final cohort was refined to encompass 448 patients, ensuring an age criterion surpassing 60 years and an ICU stay duration exceeding 24 h.

### 2.3 Variables

Based on a thorough review of the literature and clinical expertise, we incorporated additional variables into the analysis for elderly T2DM patients with cerebral infarction. These variables were deemed as potential confounding factors in determining the outcome of mortality. Their inclusion was warranted due to their collinear relationship with SpO_2_. Demographics and Admission Information: age, gender, respiratory rate, temperature, heart rate, systolic blood pressure, diastolic blood pressure, average blood pressure, Saturation of Peripheral Oxygen (SpO_2_), and Intensive Care Unit length of stay (LOS-ICU). Laboratory parameters: glucose, hematocrit, hemoglobin, platelet, white blood cell (WBC), anion gap, bicarbonate, Blood Urea Nitrogen (BUN), calcium, chloride, creatinine, potassium, International Normalized Ratio (INR), Prothrombin Time (PT), and Activated Partial Thromboplastin Time (APTT). Comorbidities: Congestive heart failure, Renal disease, Severe liver disease and COPD. Severity scores: Charlson Comorbidity Index, APSIII, SAPSII, OASIS, and GCS. Ventilation types: Invasive ventilation, Non-Invasive Ventilation (NIV), Supplemental oxygen, High flow, and Tracheotomy.

Upon admission to the Intensive Care Unit (ICU), a comprehensive set of indicators was gathered on the 1st day. These indicators encompassed factors such as age, gender, Charlson Comorbidity Index, Acute Physiology Score III (APSIII), Simplified Acute Physiology Score II (SAPSII), Oxford Acute Severity of Illness Score (OASIS), Glasgow Coma Scale (GCS), and the length of stay in the Intensive Care Unit (LOS-ICU). The comorbidities of patients were collected, including Congestive heart failure, Renal disease, Severe liver disease and COPD. Within the initial 24 h following admission to the Intensive Care Unit (ICU), the mean values of several vital indicators were documented as observations. These included respiratory rate, temperature, heart rate, systolic blood pressure, diastolic blood pressure, average blood pressure, and glucose levels. Similarly, within the same 24-h period post-ICU admission, the minimum values of various critical indicators were recorded as observations. These indicators encompassed hematocrit, hemoglobin, platelet count, White Blood Cell (WBC) count, bicarbonate levels, Blood Urea Nitrogen (BUN), calcium, chloride, creatinine, sodium, potassium, International Normalized Ratio (INR), Prothrombin Time (PT), and Activated Partial Thromboplastin Time (APTT). Furthermore, the maximum value of the anion gap was documented as an observation within the initial 24 h following admission to the ICU. In the context of the primary independent variable, SpO_2_, measurements were acquired during oxygen therapy after admission to hospital. The oxygen therapy encompassed various modalities, including High Flow Nasal Cannula (HFNC), Invasive Ventilation (InvasiveVent), Non-Invasive Ventilation (NonInvasiveVent), Supplemental Oxygen, and Tracheostomy. The median of SpO_2_ readings taken throughout the duration of oxygen therapy was employed as a representative measure of the central tendency of oxygen exposure.

### 2.4 Outcome

The primary outcome under investigation was 1-year mortality, which was defined as the vital status of patients during the 12-month period following their discharge. The death outcome of the study patients was due to massive cerebral infarction.

### 2.5 Statistical analysis

Using 1-year survival rate post-discharge as an example, we presented the distribution of baseline data for elderly T2DM patients with cerebral infarction after applying inclusion and exclusion criteria. Categorical data were presented using numerical values accompanied by corresponding percentages, while continuous data were depicted either as mean values with their associated standard deviations or as medians along with the interquartile range (IQR). This particular method offered a balanced representation of the data, capturing both the central tendencies and the spread of continuous variables. To gauge distinctions among continuous variables, the analysis of variance was applied. For categorical variables, the chi-square test was utilized to juxtapose the characteristics of the subjects within the outcome group. This meticulous approach offered a holistic perspective on the baseline characteristics, ensuring a thorough evaluation of the factors influencing 1-year survival rates post-discharge. It served to provide a robust foundation for understanding the dynamics at play in this clinical context.

SpO_2_ referred to the percentage of hemoglobin combined with oxygen in the blood to all hemoglobin that could be combined, representing the patient 's oxygen saturation. It was an important respiratory physiological parameter that could determine whether the patient had a hypoxic and hyperoxic state. Therefore, we divided the SpO_2_ of patients after inclusion and exclusion into four levels: SpO_2_ ≤ 94.5%, 94.5% < SpO_2_ ≤ 96.5%, 96.5% < SpO_2_ ≤ 98.5% and SpO_2_ > 98.5% to distinguish and compare the patient 's blood oxygen saturation level.

A restricted cubic spline analysis was conducted with the aim of ascertaining the existence of a noteworthy non-linear correlation between SpO_2_ and 1-year mortality. This analytical approach sought to elucidate whether the risk of mortality displaying either low oxygen saturation or elevated oxygen saturation levels exhibited a substantial escalation.

Cox multivariate regression was harnessed to validate and quantify the insights gleaned from the curve fitting process. Three distinct models were constructed sequentially: Model 1 (not adjusted), Model 2 (adjusted for hematocrit and Charlson Comorbidity Index), and Model 3 (adjusted for Model 2 in addition to respiratory rate and SAPSII). These models were instrumental in computing hazard ratios (HR) along with their accompanying 95 percent confidence intervals (CIs), thus shedding light on the correlation between varying SpO_2_ levels and 1-year mortality. This comprehensive analysis allowed us to assess the heightened risk of mortality among patients with diverse oxygen saturation levels, encompassing those with low, normal, high, and notably high levels.

We considered that the relationship between SpO_2_ levels and 1-year mortality might be influenced by various factors, including Sex, Age, Concomitant diseases such as Congestive heart failure, Temperature, and LOS-ICU. To investigate potential heterogeneity among subgroups, we conducted Cox subgroup analysis, and the interactions between these subgroups were assessed using the likelihood ratio test. Data analyses were carried out using packages R 4.1.2 software and Free Statistics software versions 1.5. Statistical significance was defined as a *P*-value < 0.05. This comprehensive approach allowed us to explore how these various factors may impact the relationship between SpO_2_ levels and 1-year mortality in this specific patient population.

## 3 Results

### 3.1 Population

**Figure 1:**
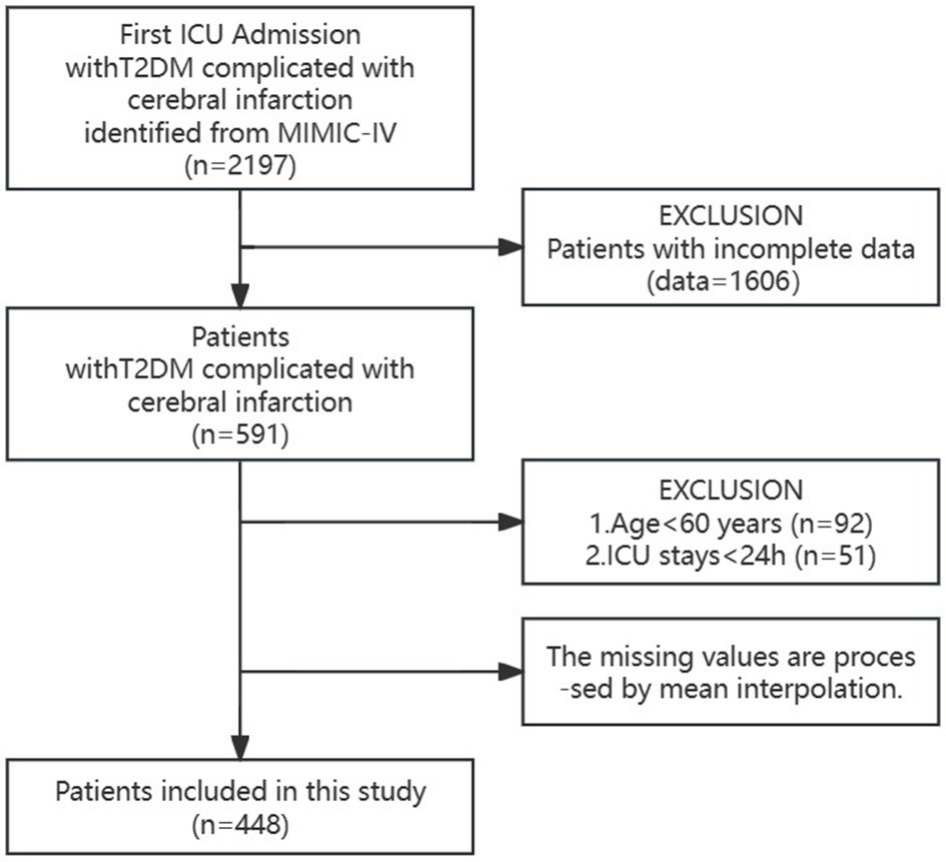
Flowchart of study patients.

In the MIMIC-IV public database, we identified and selected 2,197 elderly T2DM patients with cerebral infarction who were admitted to the ICU for the first time. Among this group, 1,606 patients were excluded due to substantial missing information, while 92 patients under the age of 60 and 51 patients with < 24 h of ICU hospitalization were also excluded. To address the missing data, mean interpolation techniques were applied. Ultimately, the final cohort comprised 448 elderly T2DM patients with cerebral infarction (as illustrated in Figure 1, the study patient flowchart).

### 3.2 Baseline characteristics

Table 1 presented a comprehensive summary of the demographic and clinical profiles of the patient cohort following their inclusion and exclusion criteria. The study encompassed 448 participants, with 226 individuals being female, constituting 50.4% of the total sample. The average age across the entire cohort was 74.0 [IQR 68.0-82.0] years, and the mean SpO_2_ recorded within 24 h after admission stood at 97.2 [IQR 95.9–98.6]%. Notably, within the follow-up period of 1 year after discharge, 161 patients (35.94%) succumbed to their conditions. It was evident that patients in the non-survival group exhibited distinct characteristics compared to their counterparts in the survival group. These patients tended to be older and presented with more severe illness upon admission [Age 78.0 [IQR 70.0–85.0] years vs. 72.0 [IQR 67.0–80.0] years, *p* < 0.001; Charlson Comorbidity Index 9.0 [IQR 8.0–11.0] vs. 8.0 [IQR 7.0–10.0], *p* < 0.001]. Their respiratory rate and heart rate were notably higher, and they had elevated average blood pressure levels when compared to the survival group. Additionally, laboratory investigations revealed that patients in the non-survival group displayed higher values for parameters such as anion gap (AG), blood urea nitrogen (BUN), and prothrombin time (PT) in comparison to the survival group. Furthermore, patients in the non-survival group had a prolonged stay in the Intensive Care Unit, indicating the gravity of their medical condition [LOS-ICU 4.2 [IQR 2.6–9.2] vs. 3.1 [IQR 1.7–5.5], *p* < 0.001]. Remarkably, a heightened prevalence of congestive heart failure was discerned among patients in the non-survival cohort, suggesting a potential comorbidity that could intricately influence their prognostic outlook. Noteworthy disparities in survival rates were also evident across patients with varying ventilation statuses, predominantly featuring Invasive ventilation and Supplemental oxygen modalities. Intriguingly, those subjected to Invasive ventilation exhibited elevated mortality rates. A paradoxical observation emerged as the non-survival group displayed unexpectedly elevated oxygen saturation levels compared to their surviving counterparts [SpO_2_ 97.6 [IQR 96.5–98.9]% vs. 96.8 [IQR 95.7–98.2]%, *p* < 0.001]. This counterintuitive result underscores the imperative for comprehensive investigation to elucidate the underlying mechanisms and unravel the nuanced clinical implications.

**Table 1:** Baseline characteristics according to survival status.

**Variables**	**All (*n* = 448)**	**Survival (*n* = 287)**	**Non-survival (*n* = 161)**	***P*-value**
**Characteristic**				
Female sex, *n* (%)	226.0 (50.4)	142.0 (49.5)	84.0 (52.2)	
Age (year)	74.0 (68.0, 82.0)	72.0 (67.0, 80.0)	78.0 (70.0, 85.0)	< 0.001
Respiratory rate (breath/minute)	18.9 (17.1, 21.1)	18.4 (16.8, 20.5)	19.6 (17.8, 21.8)	< 0.001
Temperature (°C)	36.9 (36.7, 37.1)	36.9 (36.7, 37.1)	36.9 (36.7, 37.2)	0.490
Heart rate (beats/minute)	82.4 (72.4, 90.6)	80.1 (71.1, 89.1)	85.3 (73.9, 96.1)	0.006
Systolic blood pressure (mmHg)	131.1 (116.5, 144.1)	131.7 (118.4, 144.6)	129.4 (113.8, 142.5)	0.120
Diastolic blood pressure (mmHg)	66.6 (58.2, 75.9)	67.3 (59.1, 78.4)	64.4 (57.4, 73.0)	0.007
Average blood pressure (mmHg)	83.9 (75.6, 94.1)	86.0 (77.3, 95.2)	82.4 (74.3, 91.9)	0.013
SpO_2_ (%)	97.2 (95.9, 98.6)	96.8 (95.7, 98.2)	97.6 (96.5, 98.9)	< 0.001
LOS-ICU (d)	3.5 (1.9, 6.8)	3.1 (1.7, 5.5)	4.2 (2.6, 9.2)	< 0.001
**Laboratory parameters**				
Glucose (mg/dL)	160.0 (131.2, 197.5)	155.2 (131.0, 191.0)	170.0 (133.5, 205.5)	0.051
Hematocrit (%)	32.5 ± 7.0	33.3 ± 7.0	31.0 ± 6.6	< 0.001
Hemoglobin (g/L)	10.5 (8.7, 12.3)	10.9 (9.3, 12.6)	10.0 (8.1, 11.4)	< 0.001
Platelet (10^9^/L)	189.5 (145.0, 243.0)	192.0 (148.5, 240.5)	186.0 (133.0, 243.0)	0.632
WBC (10^9^/L)	9.5 (7.3, 12.1)	9.2 (7.4, 11.6)	10.0 (7.2, 12.9)	0.116
Anion gap (mmol/L)	16.0 (14.0, 19.2)	16.0 (14.0, 18.5)	18.0 (15.0, 21.0)	< 0.001
Bicarbonate (mmol/L)	22.0 (19.0, 24.0)	22.0 (20.0, 24.0)	21.0 (18.0, 24.0)	0.020
BUN (mg/dL)	21.0 (14.0, 31.0)	19.0 (13.0, 26.5)	25.0 (16.0, 33.0)	< 0.001
Calcium (mg/dl)	8.5 (8.1, 8.9)	8.5 (8.1, 9.0)	8.5 (8.0, 8.8)	0.021
Chloride (mmol/L)	101.0 (98.0, 104.0)	101.1 (99.0, 104.0)	101.0 (97.0, 105.0)	0.734
Creatinine (mEq/L)	1.0 (0.7, 1.4)	0.9 (0.7, 1.4)	1.1 (0.8, 1.5)	0.043
Sodium (mmol/L)	138.0 (135.0, 141.0)	138.0 (135.0, 140.0)	138.0 (135.0, 141.0)	0.556
Potassium (mmol/L)	4.0 (3.7, 4.4)	4.0 (3.7, 4.3)	4.0 (3.6, 4.4)	0.946
INR	1.2 (1.1, 1.3)	1.2 (1.1, 1.3)	1.2 (1.1, 1.4)	0.016
PT (seconds)	12.8 (11.6, 14.0)	12.6 (11.4, 13.8)	13.0 (11.9, 15.0)	0.006
APTT (seconds)	27.7 (25.7, 31.2)	27.5 (25.7, 30.7)	28.2 (25.6, 32.1)	0.342
**Comorbidities**, ***n*** **(%)**				
Congestive heart failure	173.0 (38.6)	92.0 (32.1)	81.0 (50.3)	< 0.001
Renal disease	143.0 (31.9)	80.0 (27.9)	63.0 (39.1)	0.014
Severe liver disease	8.0 (1.8)	2.0 (0.7)	6.0 (3.7)	0.028
COPD	103.0 (23.0)	60.0 (20.9)	43.0 (26.7)	0.161
**Severity scores**				
Charlson Comorbidity Index	9.0 (7.8, 10.0)	8.0 (7.0, 10.0)	9.0 (8.0, 11.0)	< 0.001
APSIII	46.0 (33.0, 63.2)	40.0 (31.0, 52.0)	59.0 (43.0, 76.0)	< 0.001
SAPSII	37.0 (30.0, 44.2)	34.0 (28.0, 42.0)	40.0 (35.0, 49.0)	< 0.001
OASIS	34.0 (28.0, 40.0)	32.0 (26.0, 38.0)	38.0 (32.0, 44.0)	< 0.001
GCS	14.0 (12.0, 15.0)	14.0 (13.0, 15.0)	14.0 (10.0, 15.0)	0.231
**Ventilation types**, ***n*** **(%)**				0.491
Invasive ventilation	219.0 (48.9)	139.0 (48.4)	80.0 (49.7)	
Non-Invasive Ventilation (NIV)	14.0 (3.1)	8.0 (2.8)	6.0 (3.7)	
Supplemental oxygen	203.0 (45.3)	133.0 (46.3)	70.0 (43.5)	
High flow	3.0 (0.7)	3.0 (1.0)	0 (0)	
Tracheotomy	9.0 (2.0)	4.0 (1.4)	5.0 (3.1)	

SpO2, saturation of peripheral oxygen; ICU-LOS, Intensive Care Unit length of stay; WBC, white blood cell; BUN, blood urea nitrogen; INR, international normalized ratio; PT, Prothrombin Time; APTT, Activated partial thromboplastin time; COPD, Chronic Obstructive Pulmonary Disease; APS III, acute physiology score III; SAPSII, simplified acute physiology score II; OASIS, oxford acute severity of illness score; GCS, Glasgow Coma Scale.

### 3.3 Restricted cubic spline

**Figure 2:**
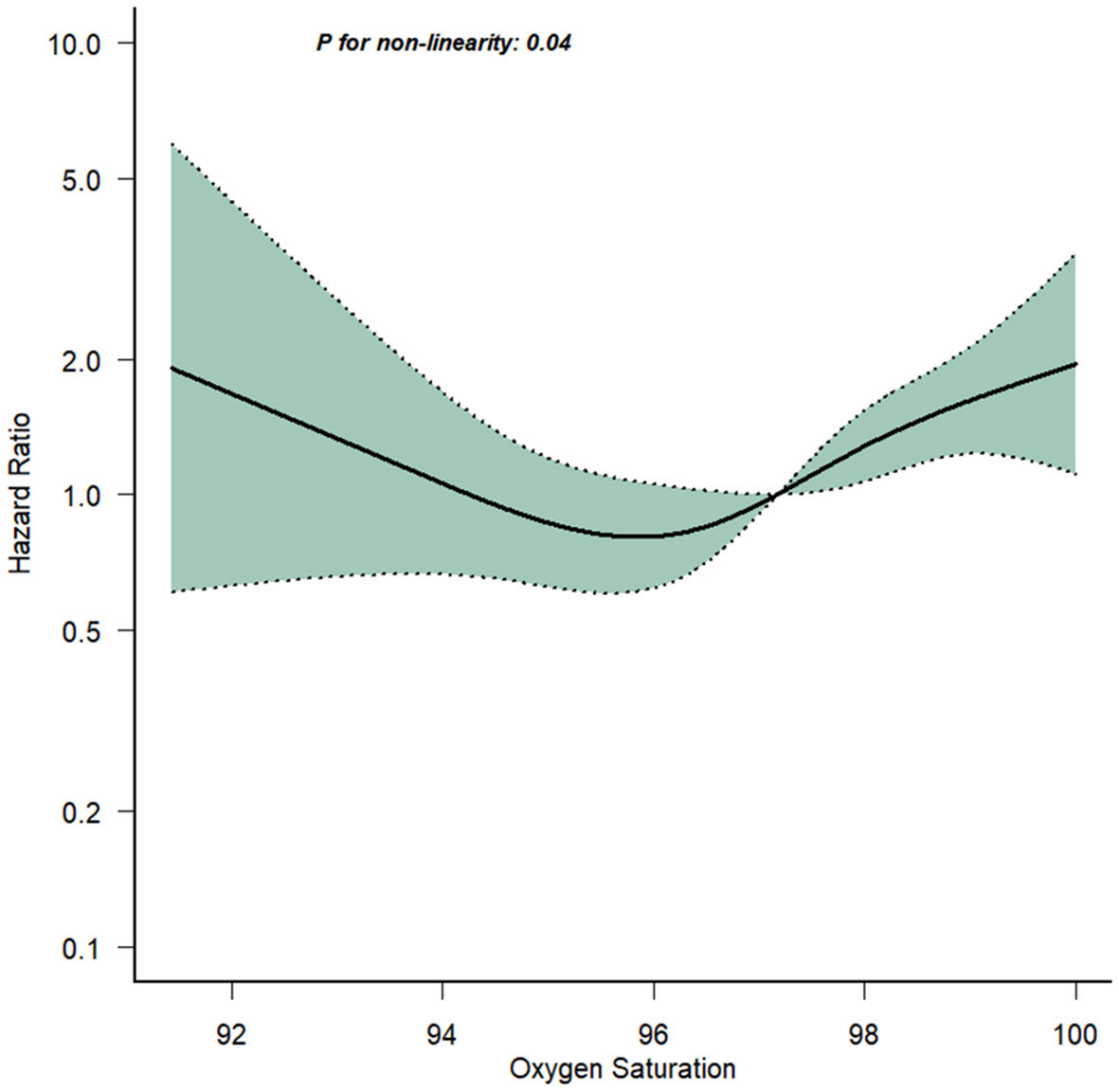
Association of the SpO_2_ and 1-year mortality.

Figure 2 illustrated the relationship between 1-year mortality and the average SpO_2_ levels recorded on the 1st day following admission. The findings revealed that low SpO_2_ levels ( ≤ 94.5%) are linked to a heightened 1-year risk of mortality. In contrast to conventional wisdom, high SpO_2_ levels (>96.5%) also correspond to an elevated 1-year mortality risk. While adjusting for covariates, specifically hematocrit and the Charlson Comorbidity Index, we employed restricted cubic spline analysis to flexibly model the association between SpO_2_ levels and 1-year mortality in elderly T2DM patients with cerebral infarction. The results unveiled a *U*-shaped pattern in the relationship between participants' SpO_2_ levels and 1-year mortality, with a statistically significant *P*-value for non-linearity (0.037). According to the restricted cubic spline analysis, the lowest range of 1-year mortality risk fell between SpO_2_ levels of 94.5–96.5%. When SpO_2_ levels were below 94.5% or above 96.5%, the fitted curve exhibited a moderately steep *U*-shaped trend. This observation indicated that both low SpO_2_ levels and high SpO_2_ levels were associated with an increased 1-year mortality risk among elderly T2DM patients with cerebral infarction. These findings challenged conventional assumptions by highlighting the complex and non-linear nature of the relationship between oxygen saturation and 1-year mortality in this patient population.

### 3.4 Multivariable Cox regression analysis

Using the Cox regression model, we presented in Table 2 both non-adjusted and adjusted estimates regarding the relationship between SpO_2_ levels and 1-year mortality in elderly T2DM patients with cerebral infarction. When SpO_2_ was used as a categorical variable, with the reference group being individuals with normal SpO_2_ levels (94.5% < SpO_2_ ≤ 96.5%), Model 1 (No adjusted) revealed that both low SpO_2_ levels ( ≤ 94.5%) and high SpO_2_ levels (96.5% < SpO_2_ ≤ 98.5%) were associated with an elevated risk of 1-year mortality [HR_l_: 1.18, HR_h_: 1.77]. Conspicuously, notably high SpO_2_ levels (>98.5%) were significantly linked to a higher 1-year mortality risk [HR: 2.25]. Model 2, which adjusted for Hematocrit and the Charlson Comorbidity Index, maintained the associations of low ( ≤ 94.5%) and high SpO_2_ levels (96.5% < SpO_2_ ≤ 98.5%) with an increased 1-year mortality risk [HR_l_: 1.4, HR_h_: 1.64]. Notably high SpO_2_ levels (>98.5%) also persisted as a significant risk factor [HR: 2.06]. In the subsequent analyses, Model 3, which incorporated additional adjustments for respiratory rate and SAPSII based on the foundation of Model 2, and Model 4, featuring further adjustments for PaO_2_/FiO_2_ and COPD based on the refined Model 3, yielded results in alignment with the outcomes of Models 1 and 2. Importantly, these supplementary adjustments did not introduce significant modifications to the identified associations between SpO_2_ levels and 1-year mortality within this specific population. This robust consistency across Models 1 to 4 underscores the robustness and reliability of the observed correlations.

**Table 2:** Hazard ratio and 95% CI of SpO_2_ for 1-year mortality.

**Variable**	**Model 1**		**Model 2**		**Model 3**		**Model 4**	
	**HR (95% CI)**	***P*** **-value**	**HR (95% CI)**	***P*** **-value**	**HR (95% CI)**	***P*** **-value**	**HR (95% CI)**	***P*** **-value**
SpO_2_ < 94.5	1.18 (0.60–2.31)	0.628	1.4 (0.71–2.76)	0.328	1.07 (0.54–2.1)	0.856	1.09 (0.54–2.2)	0.800
94.5 < SpO_2_ ≤ 96.5	1 (Reference)		1 (Reference)		1 (Reference)		1 (Reference)	
96.5 < SpO_2_ ≤ 98.5	1.77 (1.14–2.73)	0.010	1.64 (1.05–2.54)	0.028	1.62 (1.03–2.53)	0.035	1.55 (0.98–2.45)	0.063
SpO_2_ > 98.5	2.25 (1.43–3.54)	< 0.001	2.06 (1.29–3.29)	0.002	2.13 (1.31–3.44)	0.002	1.96 (1.18–3.27)	0.010

Model 1: No adjusted.

Model 2: Adjusted for Hematocrit and Charlson Comorbidity Index.

Model 3: Adjusted for Model 2 Plus Respiratory Rate and SAPSII.

Model 4: Adjusted for Model 3 Plus PaO_2_/FiO_2_ and COPD.

The analysis demonstrated that both low and high SpO_2_ levels were associated with an elevated 1-year mortality risk among elderly T2DM patients with cerebral infarction. Interestingly, the risk of mortality appeared to follow a gradient with notably high SpO_2_ levels posing the highest risk, followed by high SpO_2_ levels, low SpO_2_ levels, and finally, normal SpO_2_ levels. This finding provided a unique perspective, emphasizing that notably high oxygen saturation levels might have an adverse impact on the 1-year mortality risk, further emphasizing the non-linear and complex nature of this relationship within the context of this patient population.

### 3.5 Cumulative mortality curve analysis

**Figure 3:**
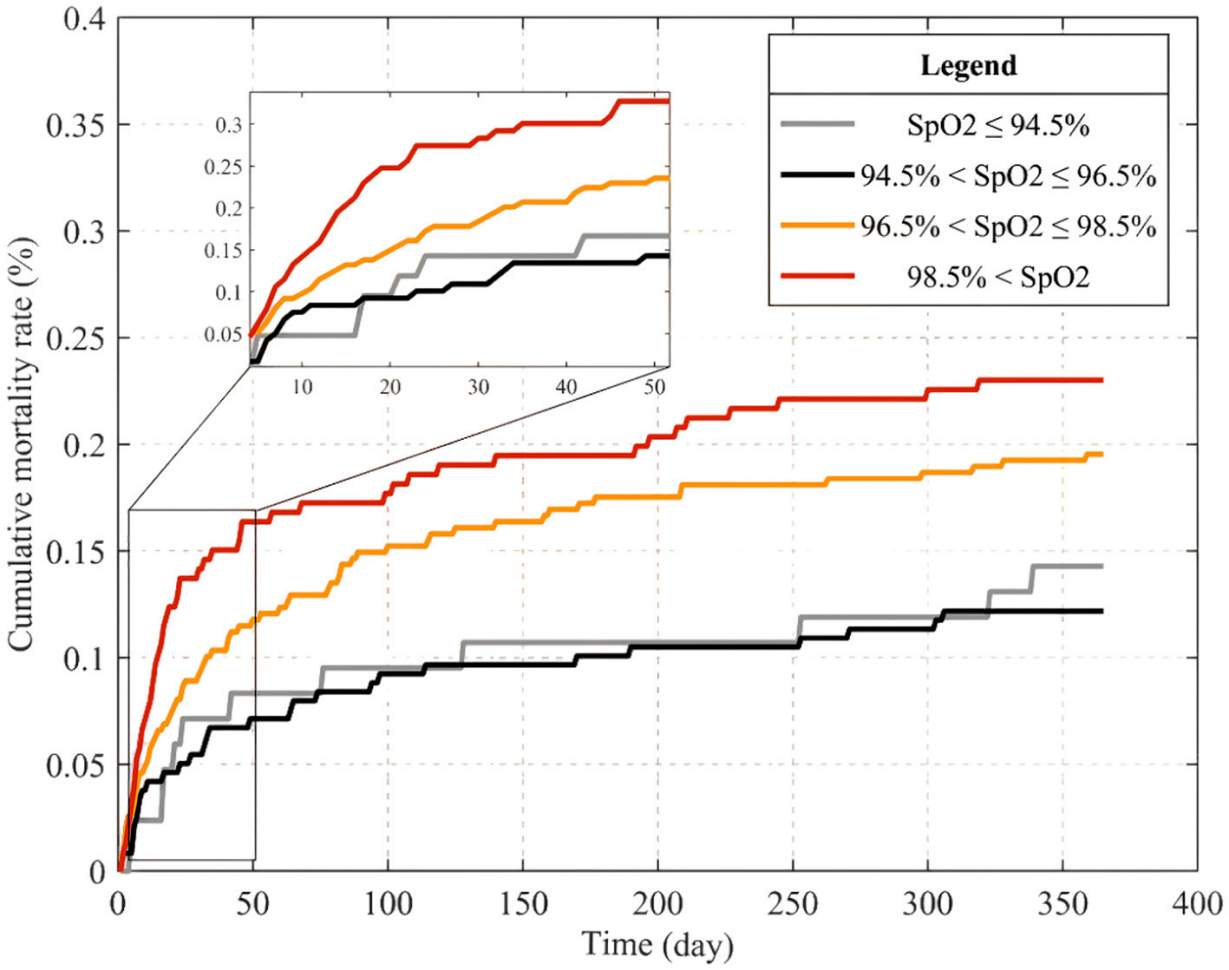
Cumulative mortality curves within 365 days of admission for patients in different SpO_2_ subgroups.

The MATLAB software was employed to meticulously calculate daily cumulative mortality rates for patients stratified into four distinct SpO_2_ subgroups over a 365-day period post-admission. Leveraging survival time data extracted from the MIMIC-IV database, the outcomes were elegantly visualized in Figure 3 using the plot() function, with detailed magnification within a graph window. Upon meticulous scrutiny and analysis of the graphical representation, a distinct pattern emerged on the 365th day, exhibiting a mortality rate hierarchy: normal SpO_2_ levels (94.5% < SpO_2_ ≤ 96.5%), low SpO_2_ levels ( ≤ 94.5%), high SpO_2_ levels (96.5% < SpO_2_ ≤ 98.5%), and notably high levels (>98.5%). Remarkably, this observed trend harmonized seamlessly with the findings derived from the Cox multivariate regression analysis.

### 3.6 Subgroup analysis

**Figure 4:**
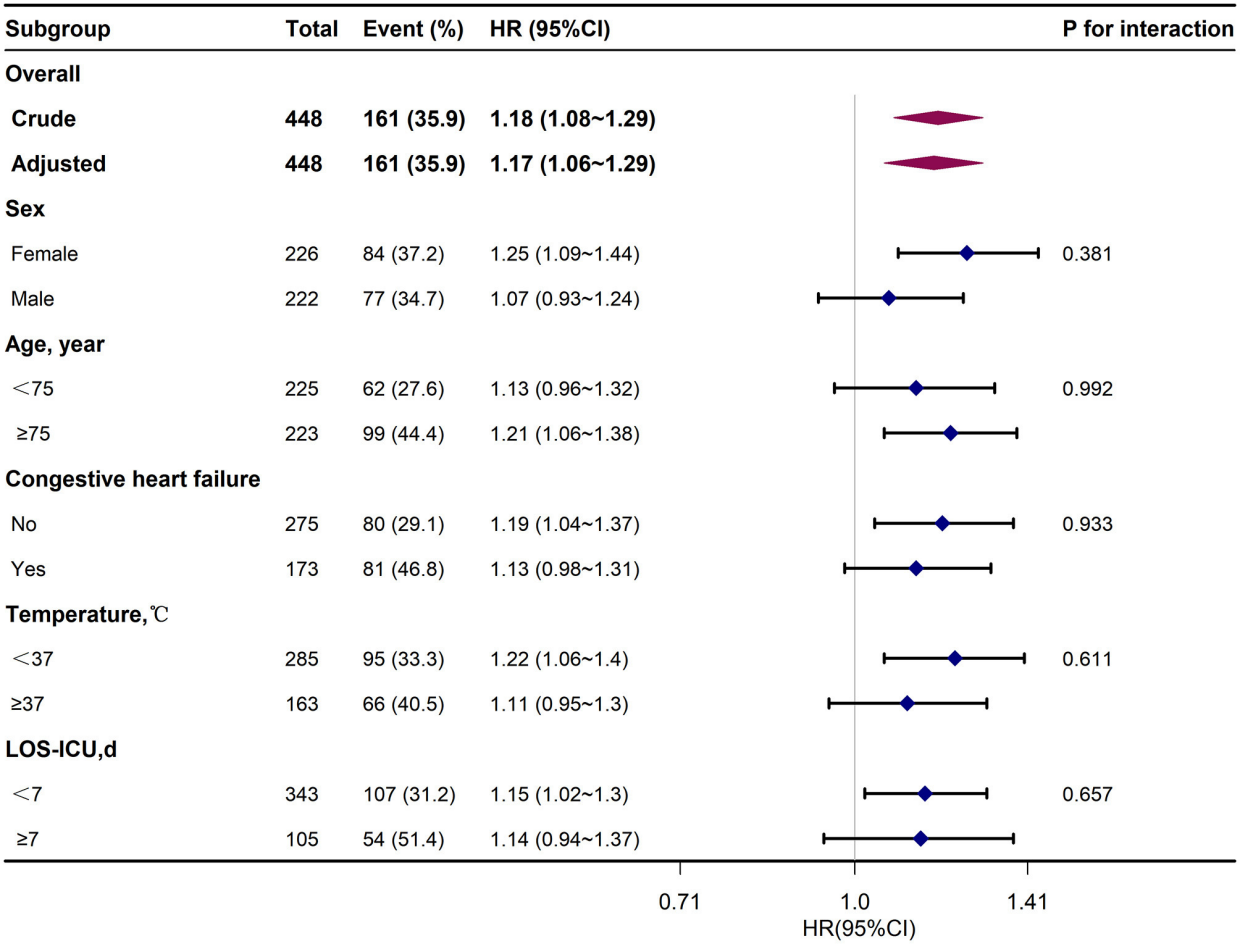
Subgroup analysis of the correlation between SpO_2_ and 365-day mortality forest map.

When conducting subgroup analysis, it was observed that there was no statistically significant interaction between SpO_2_ and the grouping variables, including Sex, Age, Congestive heart failure, Temperature and ICU length of stay (LOS-ICU; *P*-values for interaction were >0.05; as depicted in Figure 4).

## 4 Discussion

Our retrospective cohort study unveiled a pivotal association between admission SpO_2_ levels and 1-year mortality risk in elderly T2DM patients with cerebral infarction. The complexity of SpO_2_ as a continuous variable was further explored using restricted cubic splinted curves, revealing a *U*-shaped, non-linear association between SpO_2_ and 1-year mortality. Remarkably, both low and high SpO_2_ levels emerged as contributors to heightened mortality risk. Subsequent regression analysis, factoring in potential confounders, delineated elevated risks of 1-year death for both low and high SpO_2_ levels, with high levels bearing a significant increase. This nuanced relationship was vividly mirrored in Kaplan-Meier survival curves, where survival rates, descending from normal to low, high, and notably high SpO_2_ levels, highlight the impact over the 400-day trajectory.

Contrary to conventional wisdom associating higher oxygen saturation with improved patients' outcomes, a noteworthy paradox emerged from recent studies is that patients with SpO_2_ levels in the 97–100% range exhibited a significantly lower survival rate than those in the 94–98% range (11). Robust evidence showed that deviating oxygen saturation from the norm contributed to adverse consequences, including heightened risks of decompensated heart failure (12, 13). Addressing the discomfort of adult patients aligned with the British Thoracic Society's recommendation, advocating for oxygen administration to achieve a targeted saturation range of 94–98% (14). Our research resonated with above results, emphasizing that elevated oxygen saturation didn't uniformly enhance survival rates. On the contrary, abnormal saturation levels might escalate the risk of mortality, challenging the presumed benefit of universally higher oxygen saturation levels in patient care.

Hypoxia's ramifications on the human system, particularly the nervous system, were widely acknowledged, holding pivotal implications for the pathogenesis of neurological diseases like Alzheimer's and Parkinson's (15). Experiments demonstrated the potential pathogenicity of chronic hypoxia, particularly in instigating blood-brain barrier damage and subsequent neurological dysfunction (16). The centrality of the nervous system in hypoxia's impact was evident, inducing metabolic aberrations in brain tissue cells and precipitating brain edema, thereby compromising the integrity of the blood-brain barrier (17). Cerebral infarction, a prevalent macrovascular ailment in T2DM patients, manifested a substantial clinical burden marked by heightened disability and mortality rates. Ischemic stroke patients with diabetes had a high risk of stroke recurrence, and female gender was a demographic factor for poor prognosis (18). The nexus between low oxygen saturation and the onset and progression of cerebral infarction in T2DM patients was emphasized, highlighting its potential role as a prognostic marker (19). Notably, low oxygen saturation emerged as a pivotal factor linked to escalated mortality risk across diverse medical contexts. Studies revealed its independent association with mortality in COVID-19-related pneumonia, with concomitant symptoms like dyspnea (20). The intersection of oxygen saturation and health outcomes extended to conditions like Obstructive Sleep Apnea, where nocturnal hypoxemia augmented cancer risks (21). Furthermore, low SpO_2_ levels elevated the mortality risk in conditions such as cerebral embolism, metabolic encephalopathy, and subarachnoid hemorrhage. The optimal SpO_2_ range for these neurological conditions exhibited broad consistency, albeit with subtle variations (13).

At present, there is no clear evidence that low SpO_2_ levels are associated with adverse outcomes in elderly patients with type 2 diabetes mellitus and cerebral infarction. Our findings illustrated a *U*-shaped correlation between admission SpO_2_ levels and 1-year mortality in this cohort. Upon covariate adjustments, individuals with low SpO_2_ exhibited a 0.4-fold higher risk of death compared to those with normal levels. This was basically consistent with previous similar research results (13). In delineating the intricate relationship between low SpO_2_ levels and heightened mortality risk in our study cohort, the underlying mechanisms, while not fully elucidated, prompted compelling considerations. It has been suggested that low oxygen saturation might foster insulin resistance, disrupting glucose homeostasis and intensifying hyperglycemia in T2DM patients. This, in turn, complicated glycemic control, amplifying associated risks (22, 23). Moreover, low oxygen saturation could potentiate systemic inflammation and oxidative stress, marked by heightened VEGF and CRP expression, thereby fostering atherosclerosis and vascular dysfunction (24). Concomitantly, the decline in oxygen levels might unleash a surge of highly reactive free radicals, precipitating accelerated lipid and protein oxidation, culminating in tissue and organ damage (25). The impact extended to the vascular realm, where low oxygen saturation activated the adrenaline-angiotensin system, instigating vasoconstriction and impeding blood flow. This cascade induced vascular endothelial disorder, hastening the progression of endothelial dysfunction (26). In addition, the critical role of endothelial cell dysfunction in diabetes-related vascular complications has also been identified. Low oxygen saturation might exacerbate this vulnerability, amplifying mortality risks (27).

The underestimation of the risks associated with high oxygen saturation has been a prevalent issue. Contrary to the common belief that higher oxygen saturation was always beneficial, it was crucial to recognize that elevated levels might have detrimental effects on oxygen sensing, oxidative stress, and the risk of apoptotic brain injury (28). First, the potential harm of increased oxygen saturation extended to the induction of physiological abnormalities, consequently elevating the mortality risk among patients. Second, evidence supported the connection between high oxygen saturation and increased morbidity and mortality across various medical conditions. However, the threshold at which high oxygen saturation needs to be considered varies among patients with different conditions (29). Research findings suggested that severely high oxygen saturation was linked to adverse neurological outcomes and elevated mortality rates in survivors of cardiac arrest admitted to ICU (30). High oxygen saturation was linked to neurological adverse outcomes in adult patients with acute diseases, especially subarachnoid hemorrhage and cerebral infarction (31). In the case of aneurysmal subarachnoid hemorrhage, elevated oxygen saturation was correlated with poor neurological outcomes, increased mortality, and a higher incidence of DCI (32).

In this current investigation, no studies have demonstrated a correlation between high oxygen saturation and adverse outcomes in T2DM patients with cerebral infarction. Our findings, after meticulous covariate adjustments, revealed that patients with high SpO_2_ levels face a 0.64 times greater risk of mortality than those with normal SpO_2_ levels. Furthermore, the risk of death in patients with notably high SpO_2_ levels was 1.06 times higher than in those maintaining normal SpO_2_ levels. These results harmonized with earlier research (13). However, the intricate relationship between high SpO_2_ and heightened mortality in elderly T2DM patients with cerebral infarction remained enigmatic, but several plausible mechanisms have been proposed. In this demographic, the coexistence of hyperglycemia, hypertension, and lipid metabolism disorders might expedite atherosclerosis, intensifying the risk of fatal cerebral infarction (5, 33). High oxygen saturation held the potential to incite intracellular oxidative stress, instigating an inflammatory cascade. This response might prompt the release of inflammatory mediators and platelet activation, fostering atherosclerotic plaque development and escalating mortality risk (34, 35). Concurrently, the inflammatory milieu might disrupt normal glucose metabolism, amplifying susceptibility to cardiovascular events and mortality (36, 37). In addition, high oxygen saturation might induce cellular damage, encompassing apoptosis and necrosis, exacerbating brain tissue injury and worsening cerebral infarction (38, 39).

Nonetheless, it's imperative to acknowledge several noteworthy limitations in this study. Firstly, within the MIMIC database, the availability of covariates, such as smoking and drinking history, was not attainable, leaving potential residual covariates unaccounted for, as is typical in retrospective cohort analyses. Moreover, upon preliminary data sorting, a small number of missing values were identified. While we made extensive efforts to mitigate this issue through mean interpolation, the possibility of bias persisting between the obtained results and actual data cannot be entirely eliminated. Secondly, this study is inherently constrained by its single-center retrospective cohort design, which lacks the advantages associated with other research methodologies. Thirdly, this retrospective cohort analysis can only establish an association between SpO_2_ levels and 1-year mortality, rather than establishing a direct causal relationship. However, the observed relationship in this study between low and high SpO_2_ levels and 1-year mortality in patients is compelling. Fourthly, the conclusions drawn from this retrospective cohort study require validation through further prospective experiments with extended follow-up periods.

## 5 Conclusion

In elderly T2DM patients with cerebral infarction, it was observed that both excessively high and excessively low levels of SpO_2_ were associated with an elevated risk of mortality. Maintaining SpO_2_ within the range of 94.5–96.5% appears to be associated with the lowest mortality rate. It's important to note that further experimental investigations are warranted to substantiate this conclusion.

## Data availability statement

The data analyzed in this study was obtained from the Medical Information Mart for Intensive Care IV (MIMIC-IV) database. To access the files, users must be credentialed users, complete the required training (CITI Data or Specimens Only Research), and sign the data use agreement for the project. Requests to access these datasets should be directed to PhysioNet, https://physionet.org/, https://doi.org/10.13026/s6n6-xd98.

## Ethics statement

The research involving human participants underwent a thorough review and received approval from both the Massachusetts Institute of Technology and Beth Israel Deaconess Medical Center. The studies were conducted in accordance with the local legislation and institutional requirements. The Ethics Committee/institutional review board waived the requirement of written informed consent for participation from the participants or the participants' legal guardians/next of kin. This practice was in alignment with both national legislation and institutional regulations.

## Author contributions

SZ: Conceptualization, Data curation, Formal analysis, Funding acquisition, Investigation, Methodology, Project administration, Resources, Writing—original draft, Writing—review & editing. JJ: Conceptualization, Data curation, Formal analysis, Funding acquisition, Writing—review & editing. SG: Investigation, Methodology, Project administration, Resources, Writing—review & editing. SY: Software, Supervision, Validation, Visualization, Writing—review & editing. ZS: Conceptualization, Data curation, Formal analysis, Funding acquisition, Writing—review & editing. JLi: Conceptualization, Data curation, Funding acquisition, Investigation, Methodology, Resources, Software, Supervision, Visualization, Writing—review & editing. JLiu: Conceptualization, Data curation, Funding acquisition, Investigation, Methodology, Resources, Software, Supervision, Visualization, Writing—review & editing.
