# Supplementary material for: Association between SpO2 and the risk of death in elderly T2DM patients with cerebral infarction: a retrospective cohort study
Source: Front Neurol. 2024 Mar 12;15:1344000. doi: 10.3389/fneur.2024.1344000 (PMC10964770; doi:10.3389/fneur.2024.1344000)
Supplement: Supplementary file 1 [file Data_Sheet_1.PDF]

Supplementary Material

Association Between SpO2 and The Risk of Death in Elderly T2DM Patients with Cerebral Infarction: A Retrospective Cohort Study

Shuo Zhang[1], Jiaqi Ji[1], Siqi Gao[2], Shu Yang[1], Zeyi Song[1], Jianmin Li[1], Junjie Liu[1*]

[1] School of Clinical Medicine, North China University of Science and Technology, Tangshan, China

[2] School of Basic Medical Sciences, North China University of Science and Technology, Tangshan, China

*** Correspondence:**
Corresponding Author: Junjie Liu
linchuangljj@ncst.edu.cn

1 Supplementary Figures and Tables

1.1 Supplementary Figures

The four levels of SpO2 (SpO2≤94.5, 94.5<SpO2≤96.5, 96.5<SpO2≤98.5, SpO2>98.5) were determined by pre-experiments. Not included in the article due to space limitations. The details are as follows: with the 1-year mortality of elderly T2DM patients with cerebral infarction as the dependent variable and SpO2 as the independent variable, computer simulation was carried out based on MATLAB software, and the data of 448 patients were randomly sampled 50 times, with 300 patients selected for each time. Fifty relationship curves were constructed using restricted cubic splines. It was observed that when the SpO2 of patients was in the range of (94.5,96.5), the risk of death of patients was very low. At the same time, moving 2% to the right, we established an SpO2 cutoff point of 98.5% for high and notably high oxygen-saturation to compare survival in these two conditions.

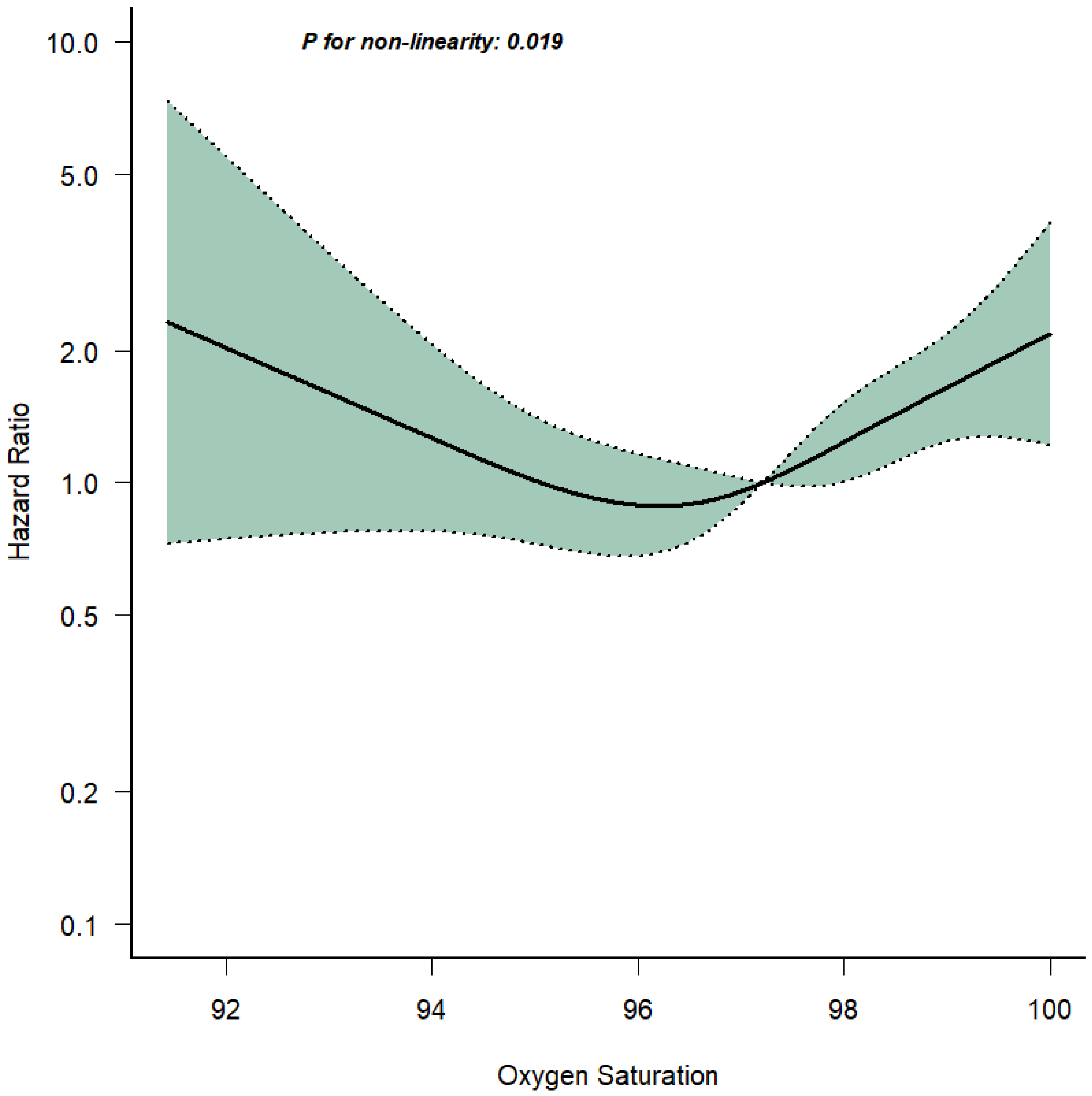
P for non-linearity: 0.019
Hazard Ratio
10.0
5.0
2.0
1.0
0.5
0.2
0.1
92
94
96
98
100
Oxygen Saturation

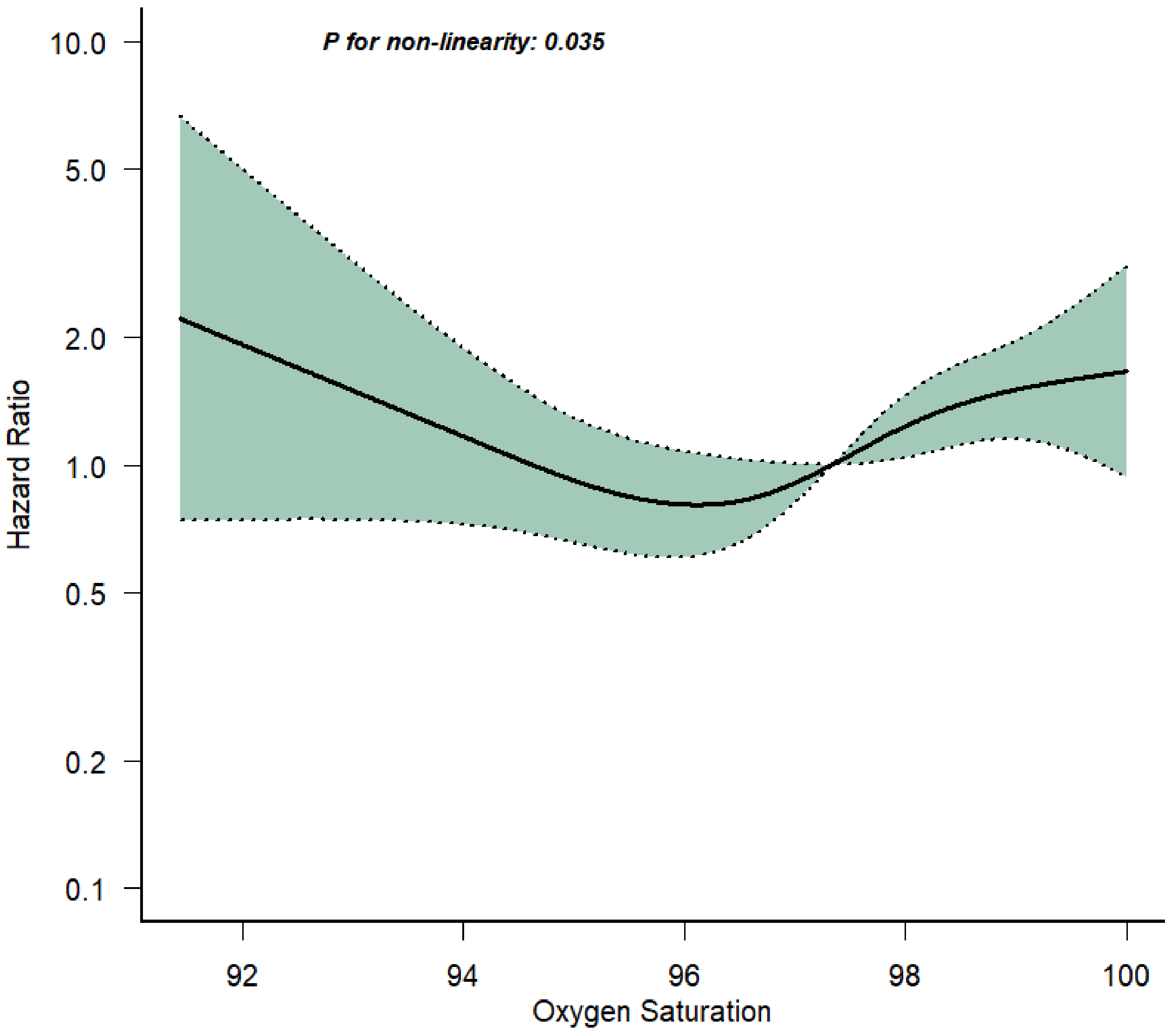

P for non-linearity: 0.035
10.0
5.0
2.0
1.0
0.5
0.2
0.1
Hazard Ratio
92
94
96
98
100
Oxygen Saturation

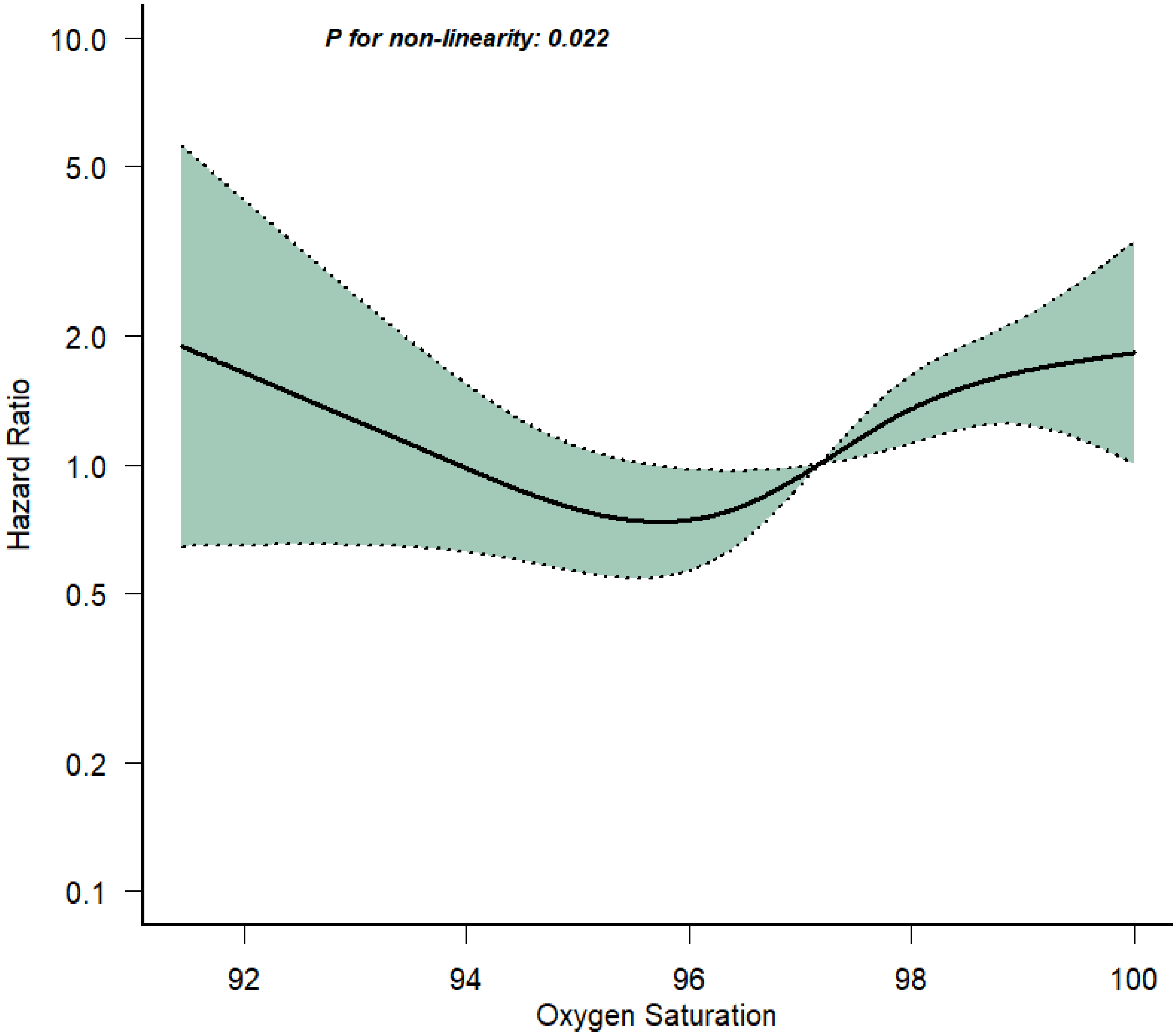
P for non-linearity: 0.022
Hazard Ratio
10.0
5.0
2.0
1.0
0.5
0.2
0.1
92
94
96
98
100
Oxygen Saturation

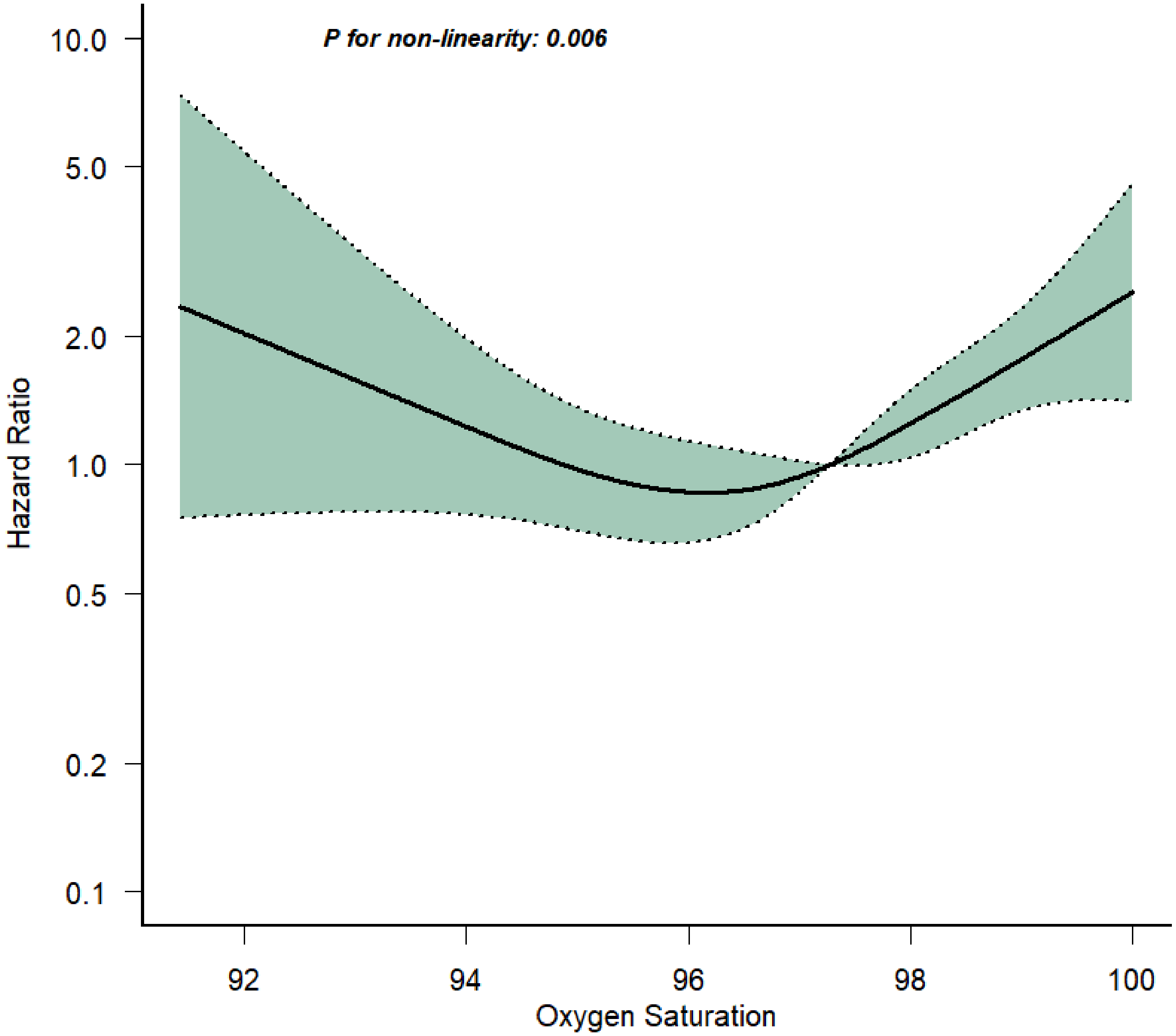

P for non-linearity: 0.006
10.0
5.0
2.0
1.0
0.5
0.2
0.1
Hazard Ratio
92
94
96
98
100
Oxygen Saturation

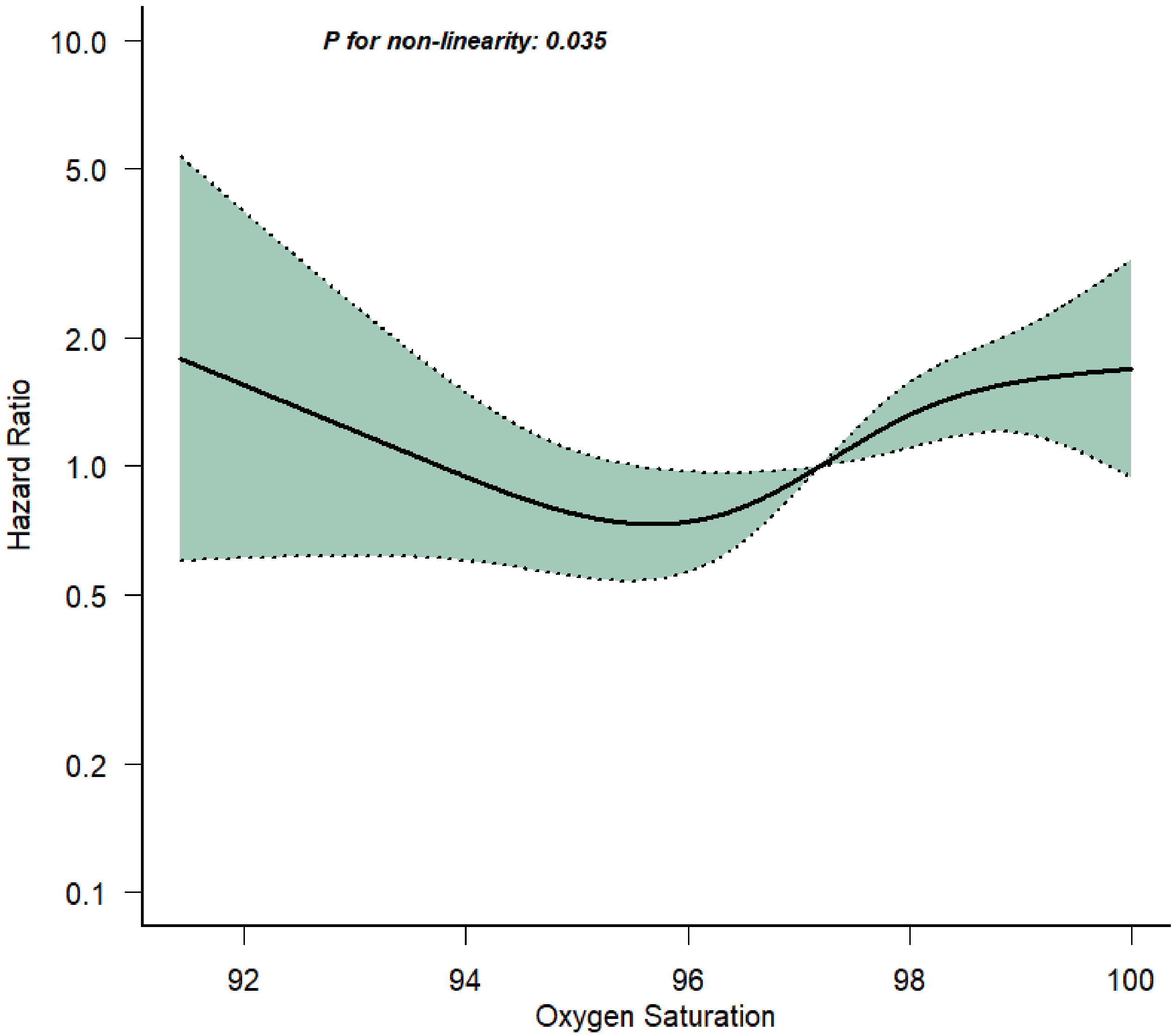
P for non-linearity: 0.035
10.0
5.0
2.0
1.0
0.5
0.2
0.1
Hazard Ratio
92
94
96
98
100
Oxygen Saturation

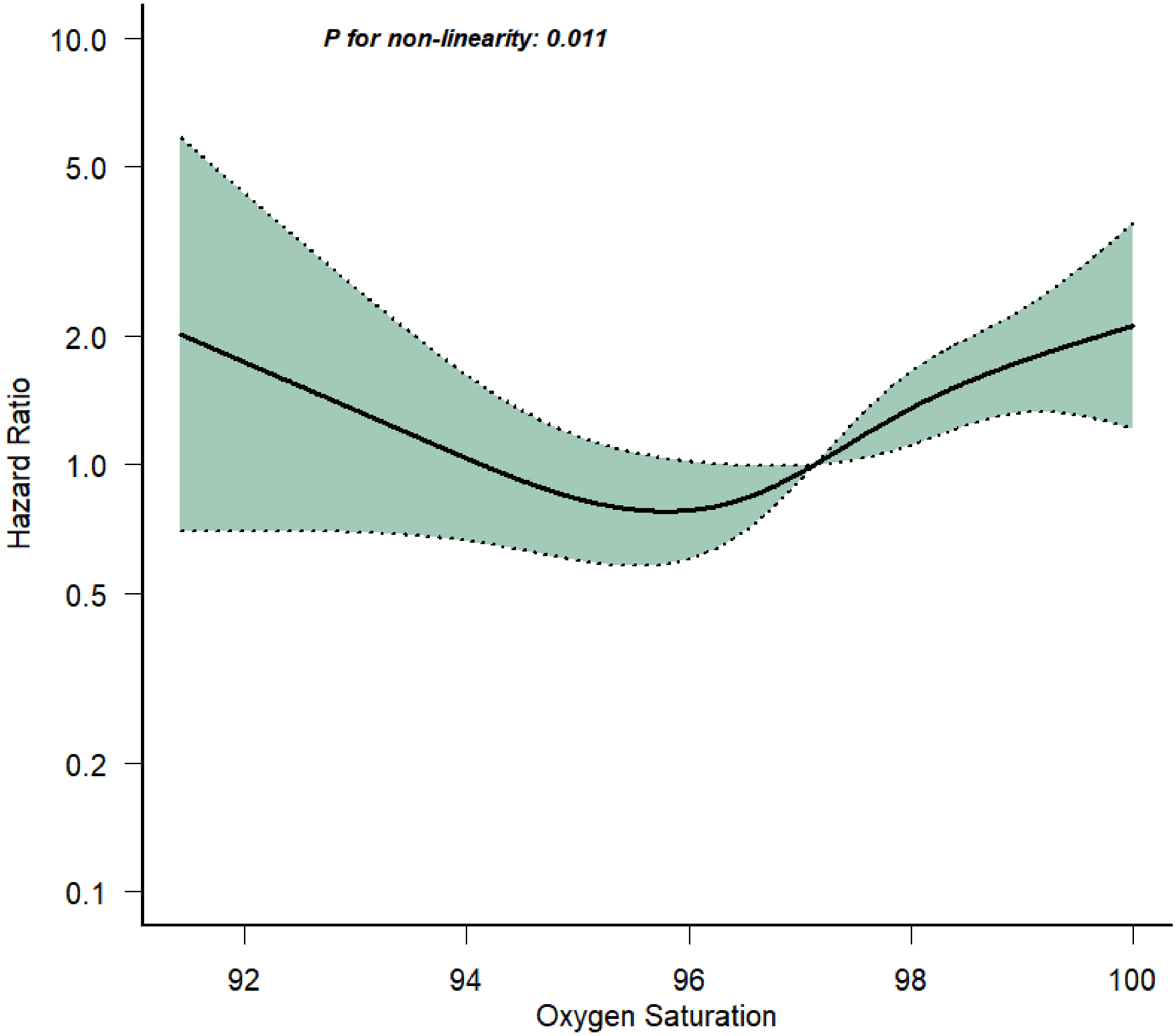

P for non-linearity: 0.011
10.0
5.0
2.0
1.0
0.5
0.2
0.1
Hazard Ratio
92
94
96
98
100
Oxygen Saturation

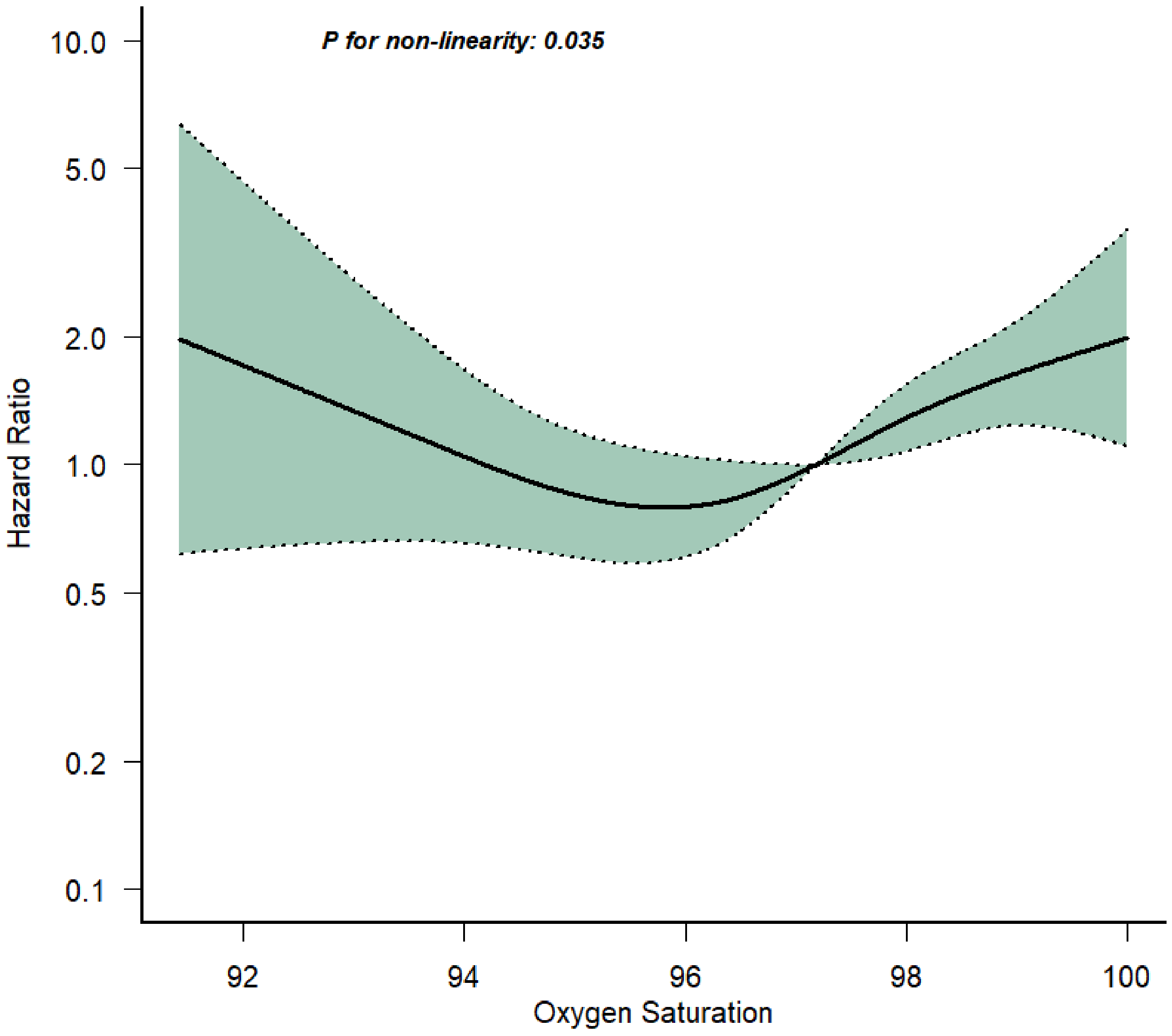
P for non-linearity: 0.035
10.0
5.0
2.0
1.0
0.5
0.2
0.1
Hazard Ratio
92
94
96
98
100
Oxygen Saturation

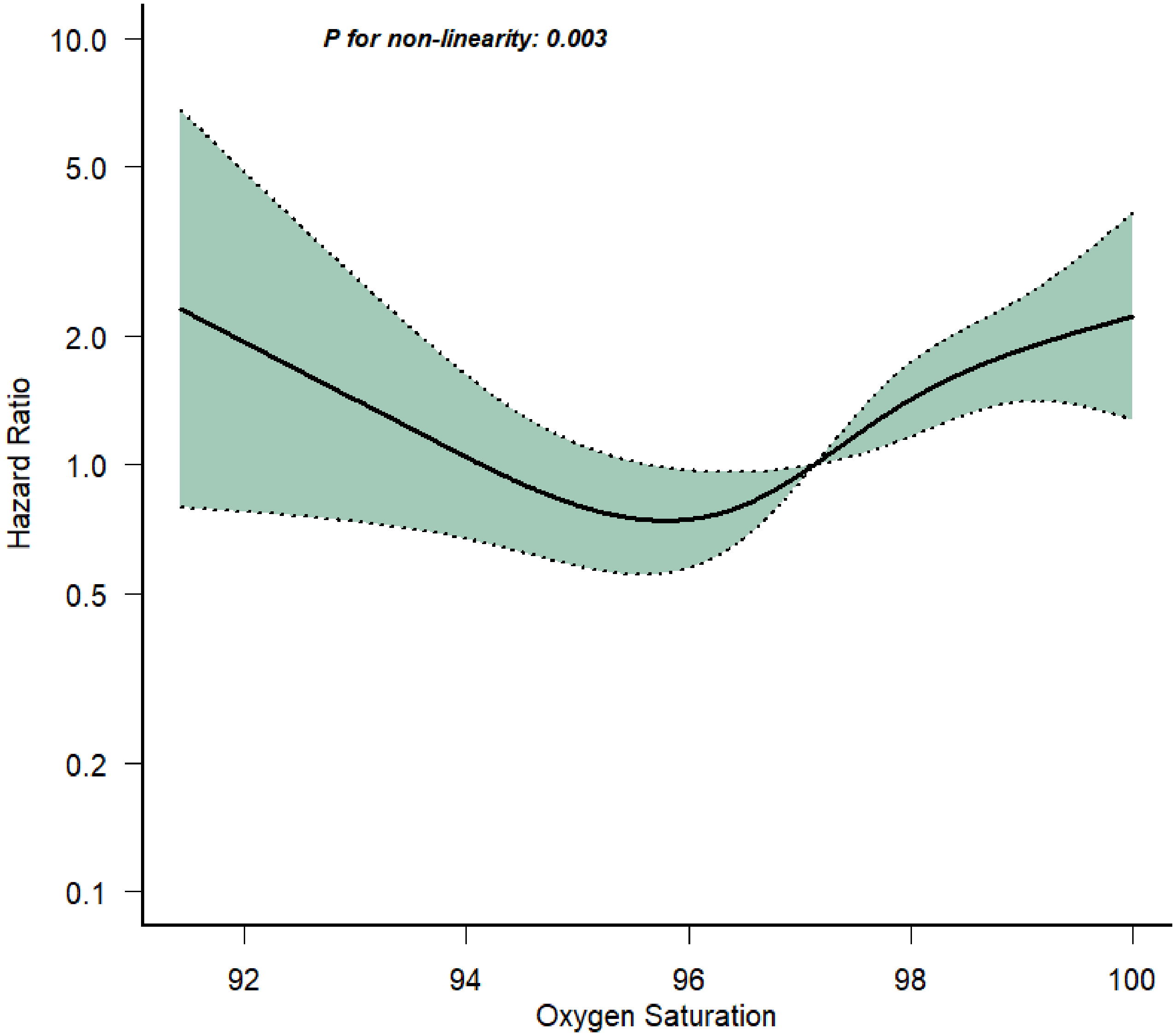
P for non-linearity: 0.003
Hazard Ratio
10.0
5.0
2.0
1.0
0.5
0.2
0.1
92
94
96
98
100
Oxygen Saturation

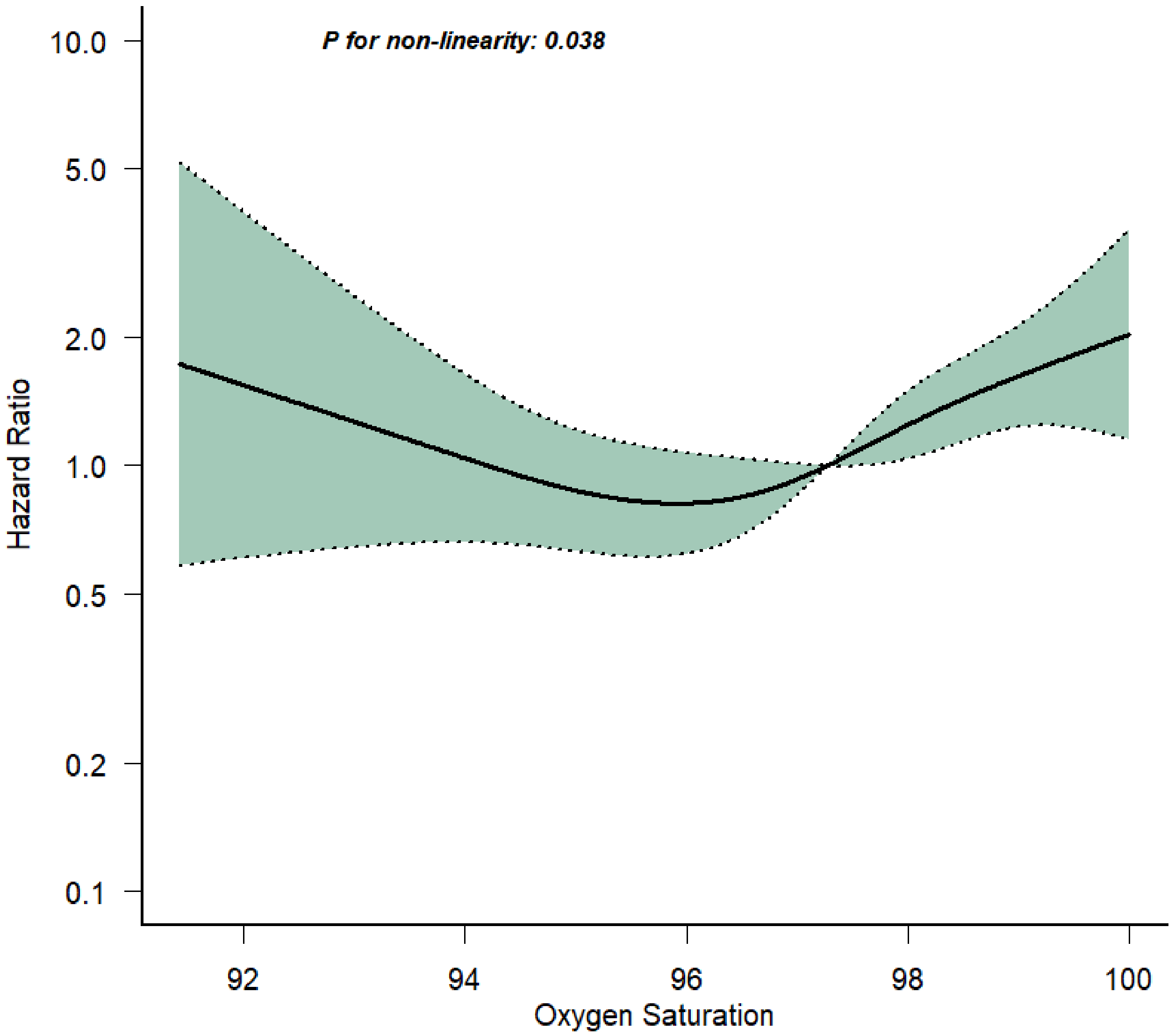
P for non-linearity: 0.038
Hazard Ratio
10.0
5.0
2.0
1.0
0.5
0.2
0.1
92
94
96
98
100
Oxygen Saturation

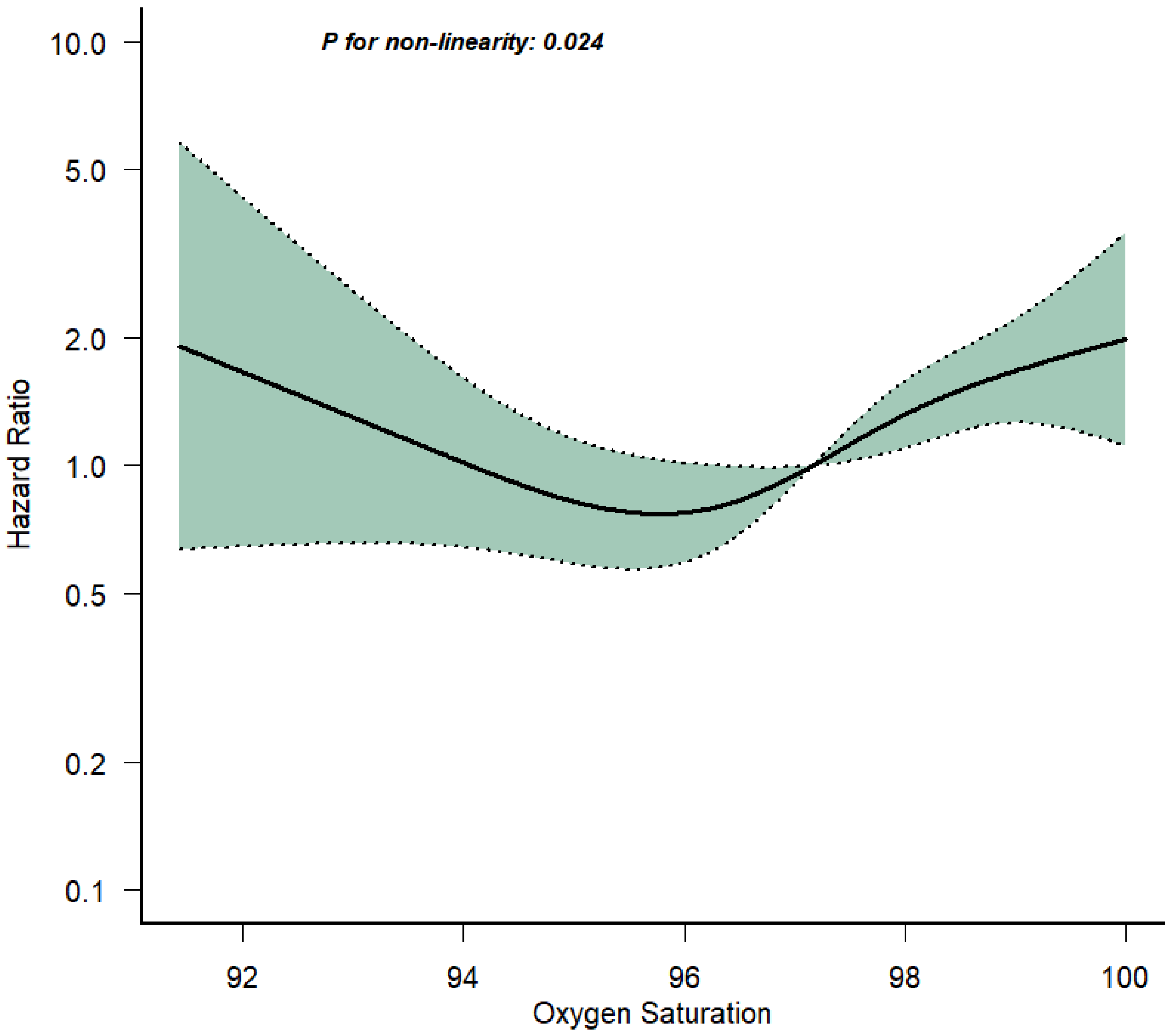
P for non-linearity: 0.024
10.0
5.0
2.0
1.0
0.5
0.2
0.1
Hazard Ratio
92
94
96
98
100
Oxygen Saturation

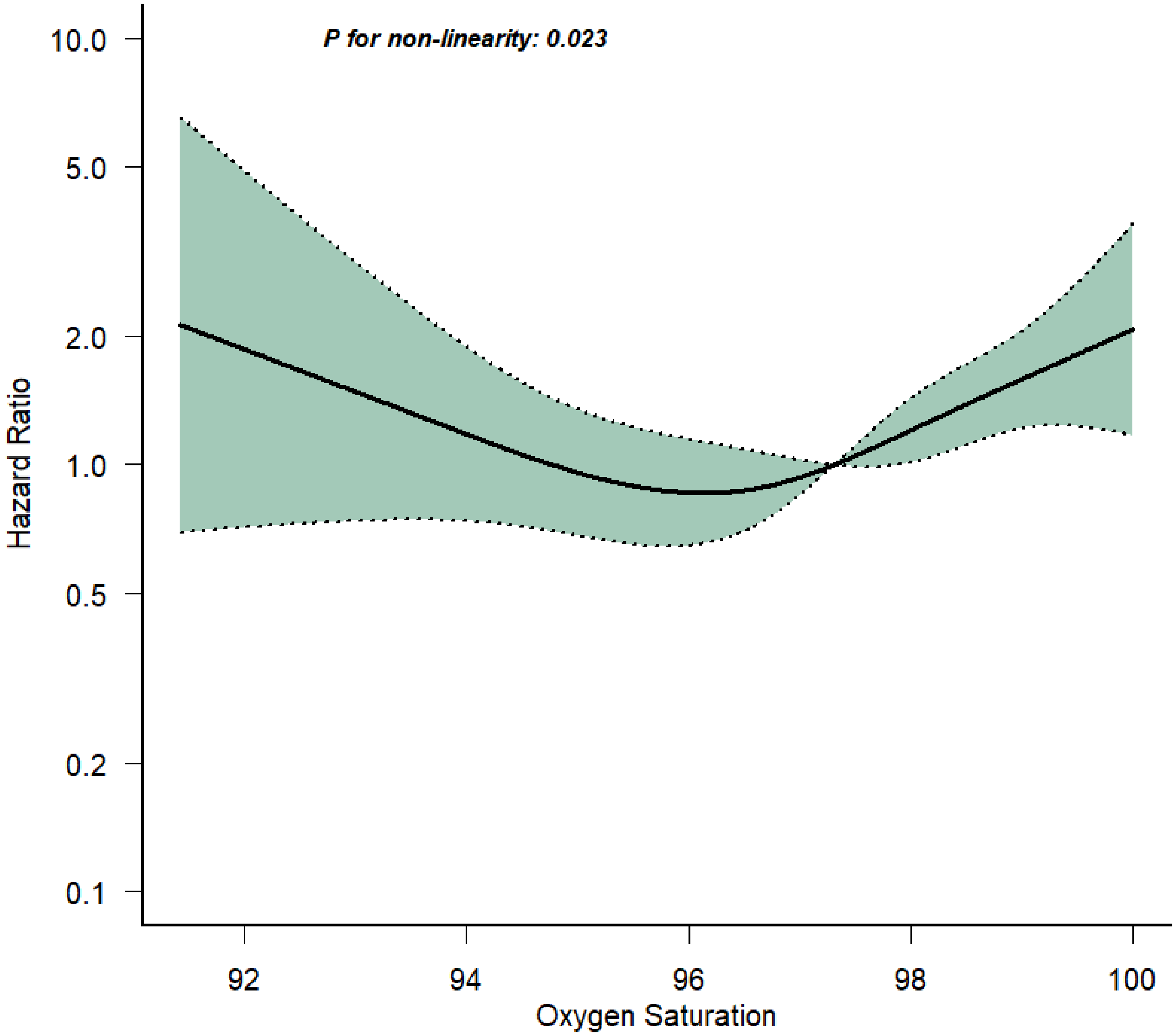
P for non-linearity: 0.023
Hazard Ratio
10.0
5.0
2.0
1.0
0.5
0.2
0.1
92
94
96
98
100
Oxygen Saturation

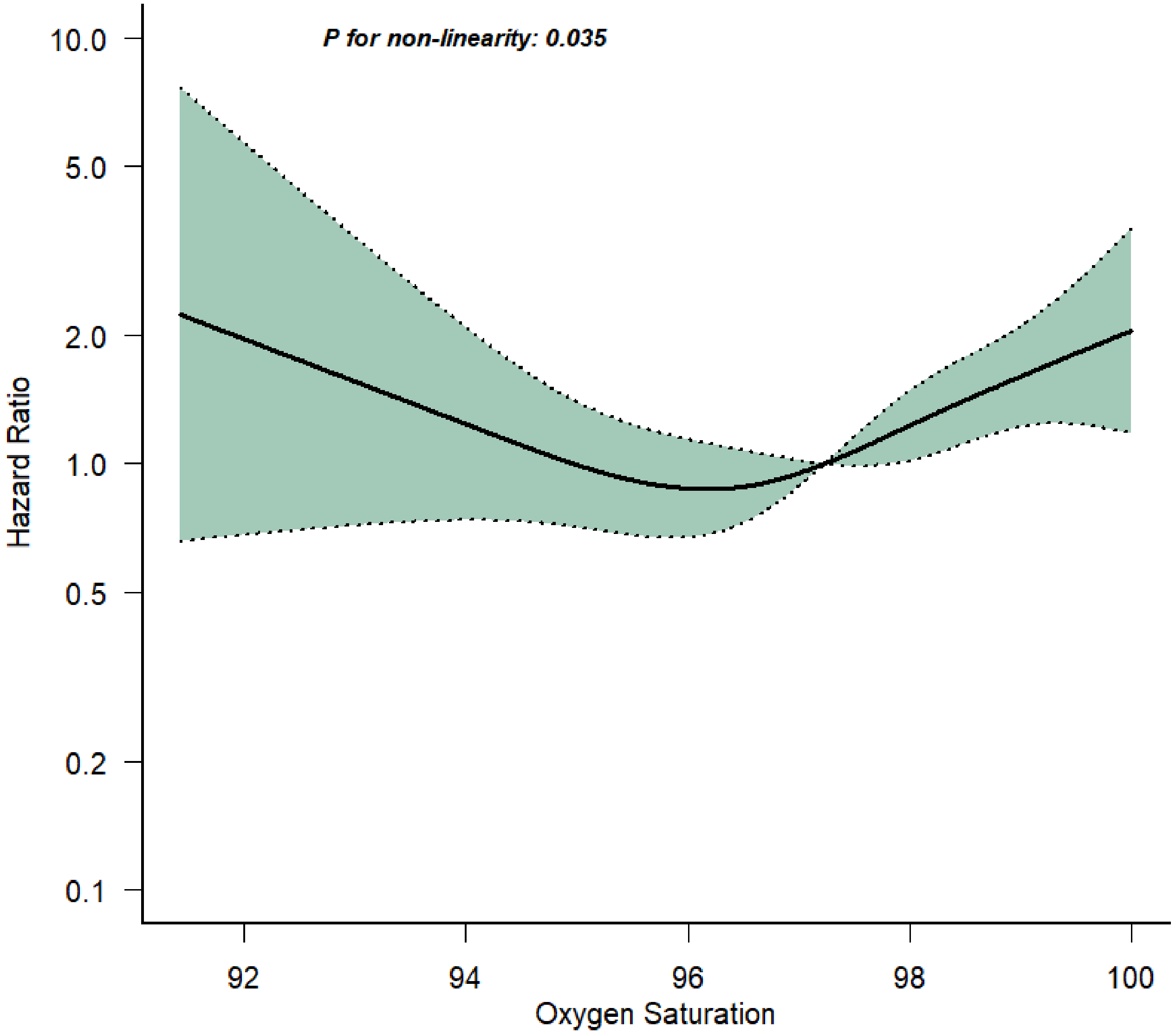
P for non-linearity: 0.035
10.0
5.0
2.0
1.0
0.5
0.2
0.1
Hazard Ratio
92
94
96
98
100
Oxygen Saturation

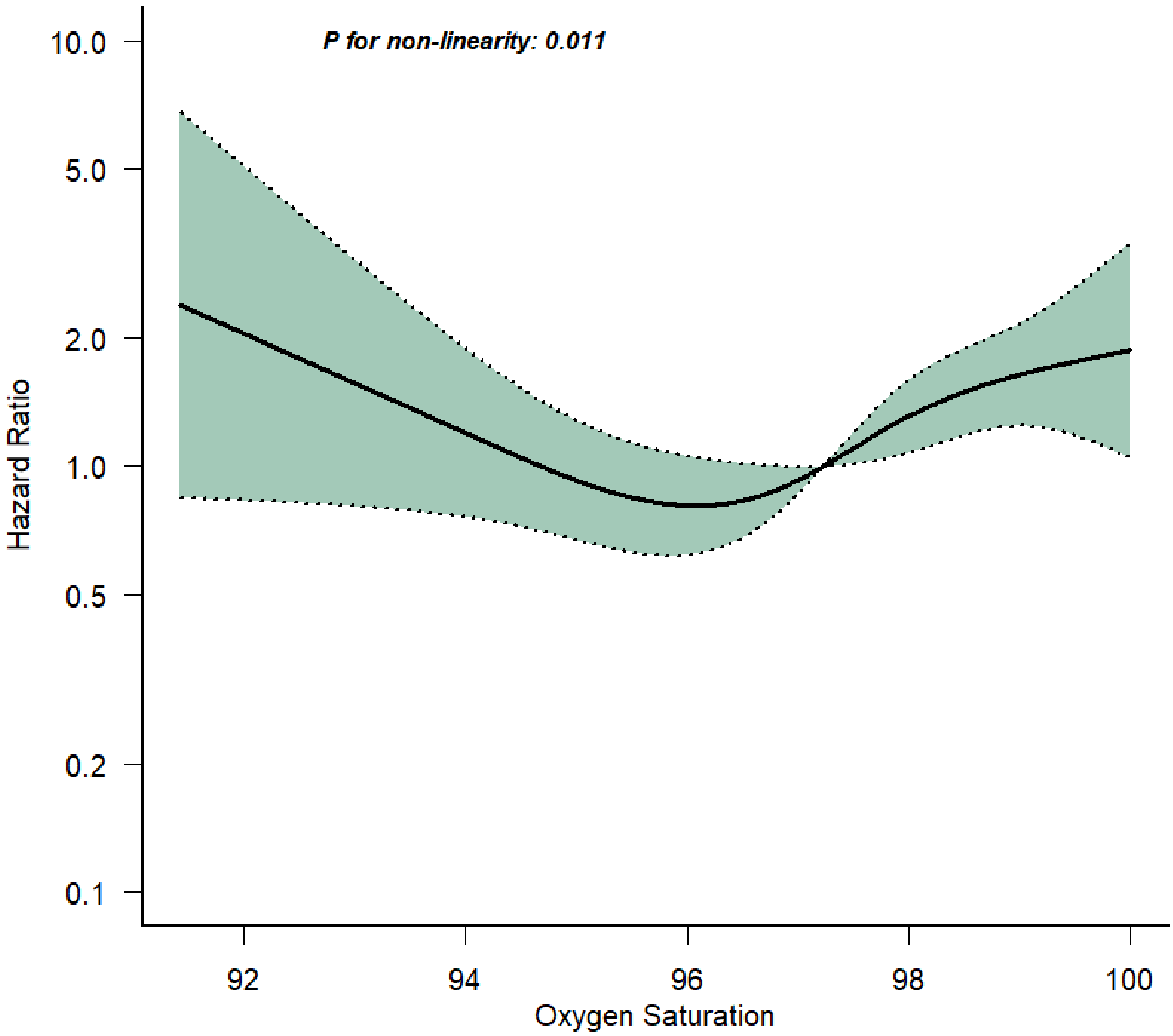
P for non-linearity: 0.011
10.0
5.0
2.0
1.0
0.5
0.2
0.1
Hazard Ratio
92
94
96
98
100
Oxygen Saturation

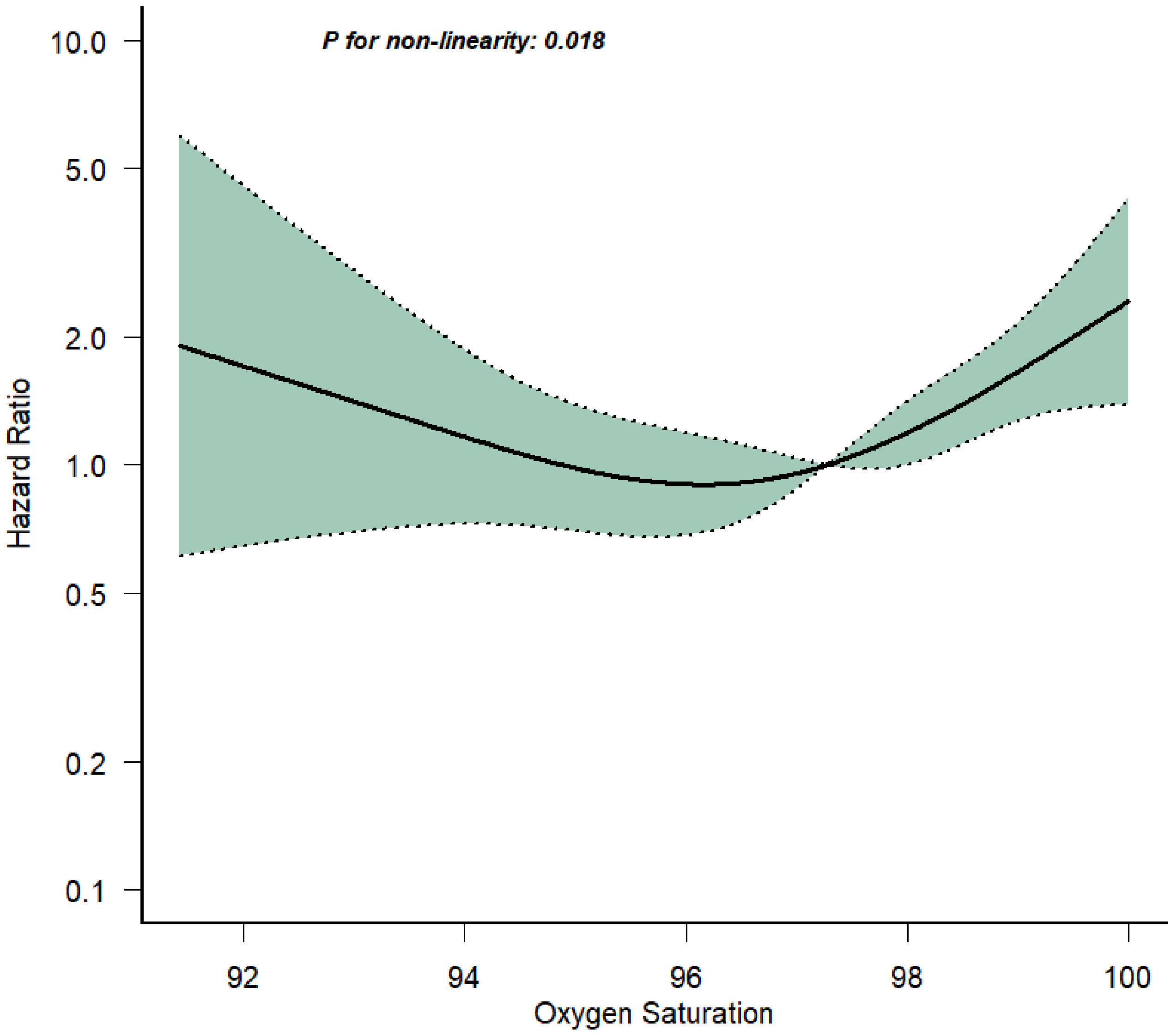
P for non-linearity: 0.018
10.0
5.0
2.0
1.0
0.5
0.2
0.1
Hazard Ratio
92
94
96
98
100
Oxygen Saturation

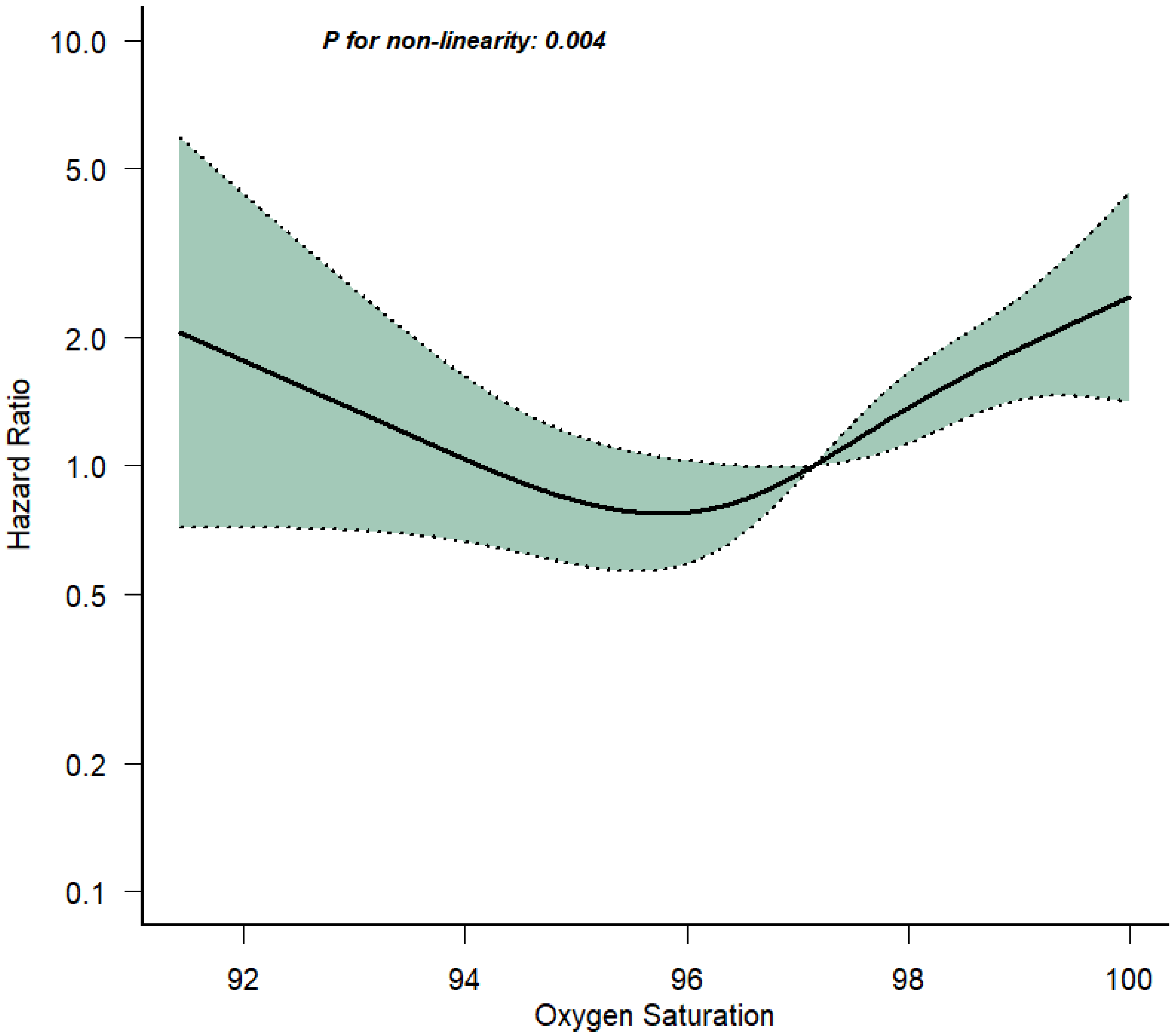
P for non-linearity: 0.004
10.0
5.0
2.0
1.0
0.5
0.2
0.1
Hazard Ratio
92
94
96
98
100
Oxygen Saturation

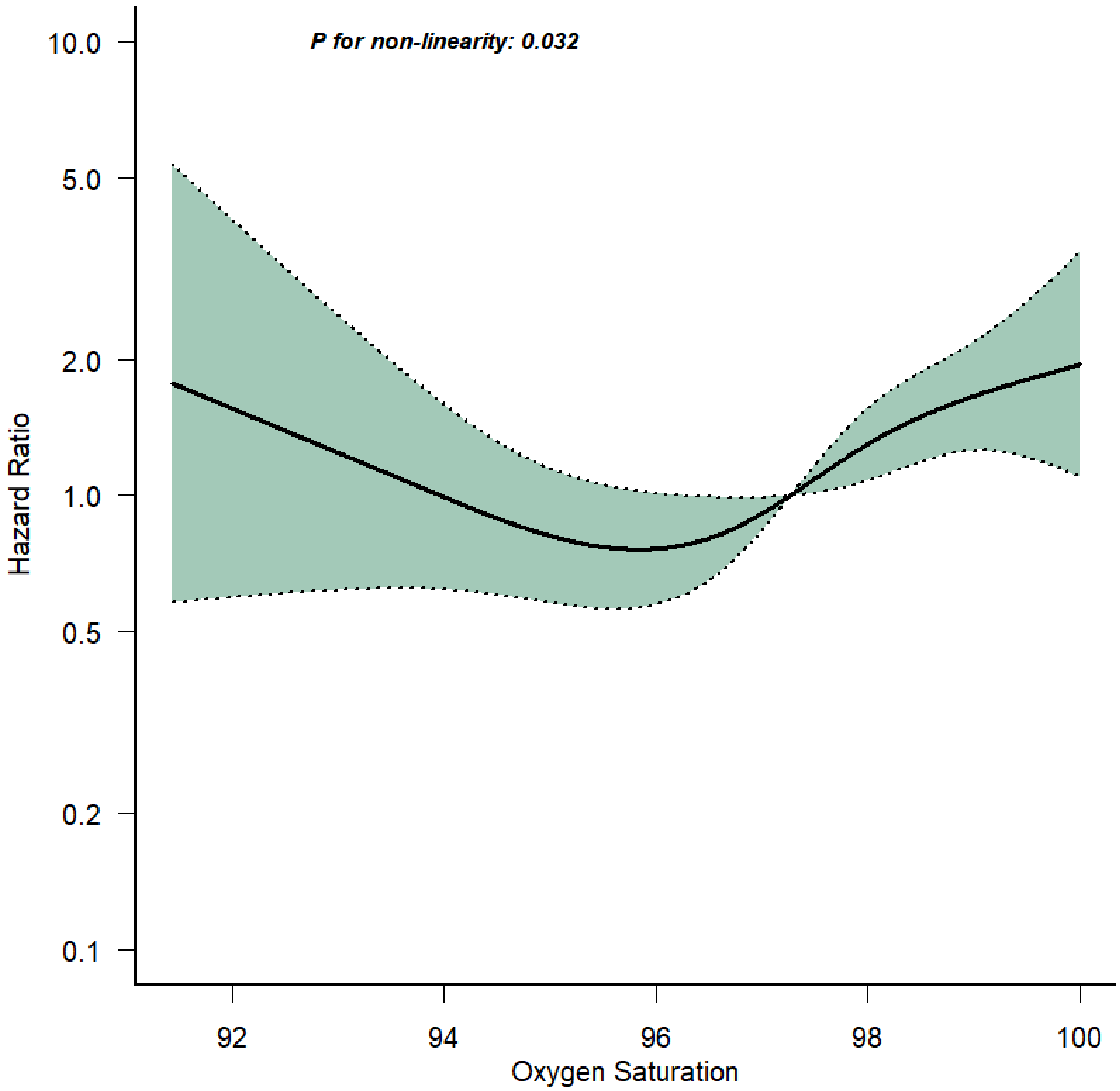

P for non-linearity: 0.032
10.0
5.0
2.0
1.0
0.5
0.2
0.1
Hazard Ratio
92
94
96
98
100
Oxygen Saturation

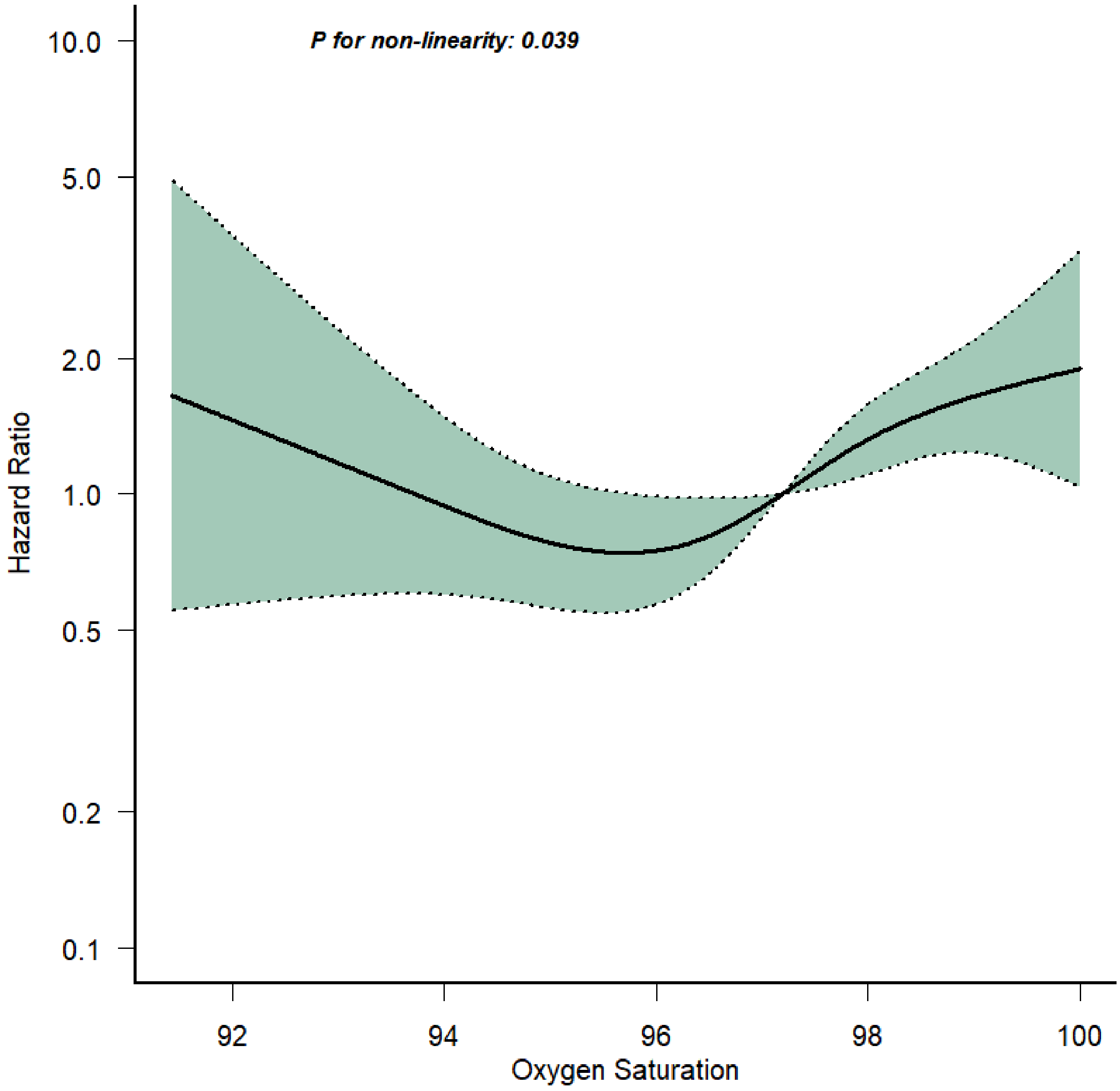
P for non-linearity: 0.039
10.0
5.0
2.0
1.0
0.5
0.2
0.1
Hazard Ratio
92
94
96
98
100
Oxygen Saturation

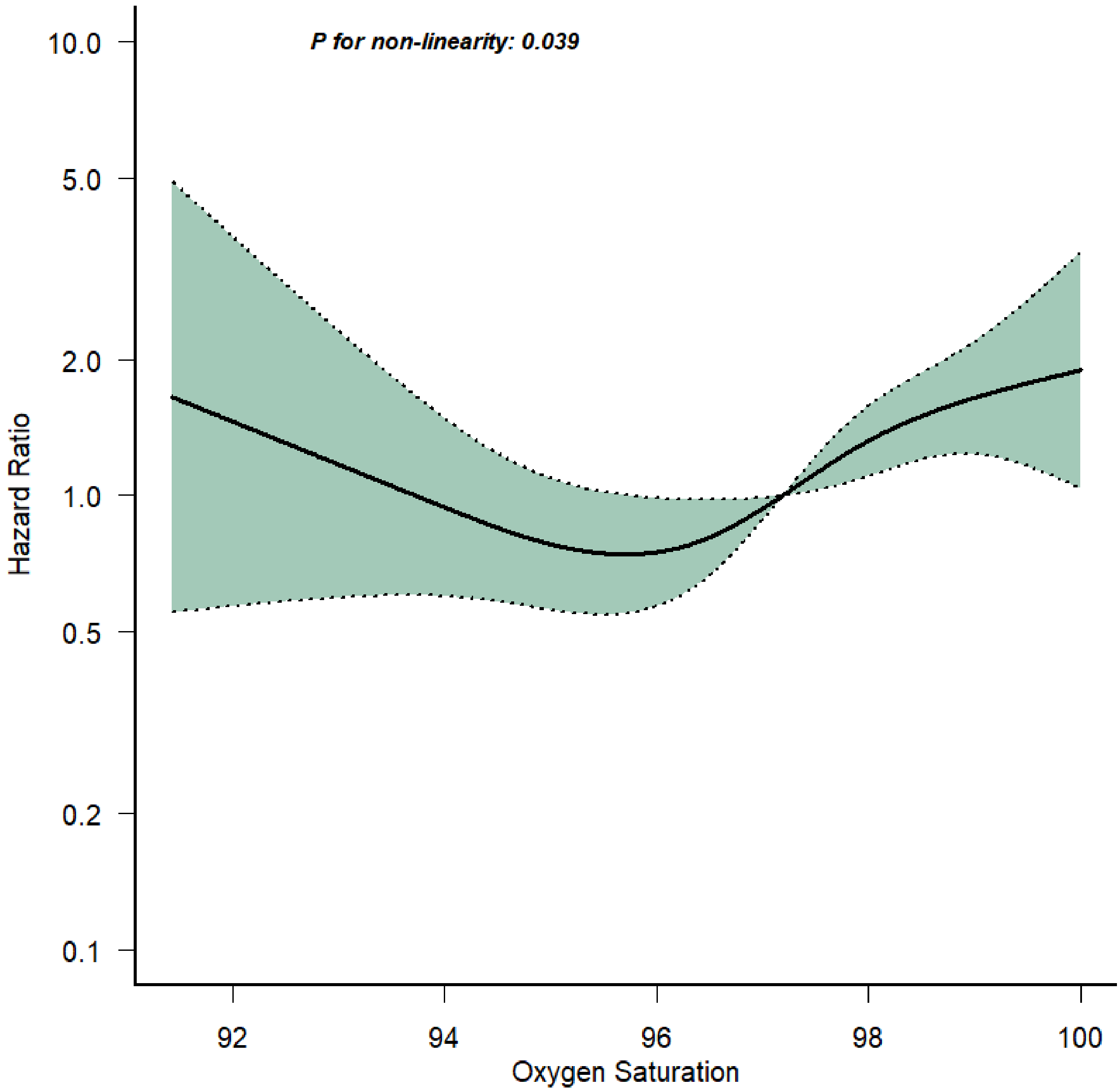

P for non-linearity: 0.039
10.0
5.0
2.0
1.0
0.5
0.2
0.1
Hazard Ratio
92
94
96
98
100
Oxygen Saturation

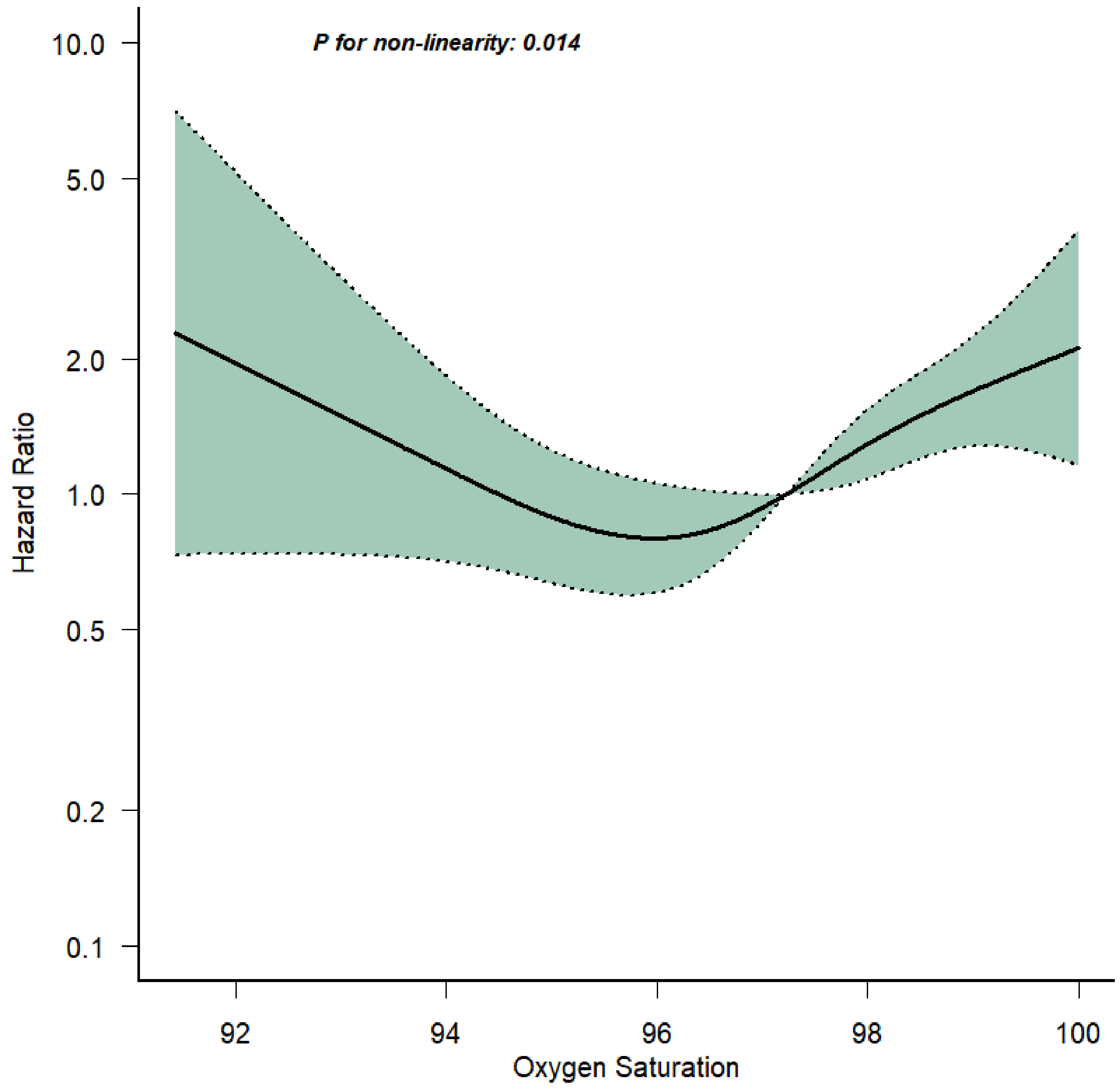
P for non-linearity: 0.014
Hazard Ratio
10.0
5.0
2.0
1.0
0.5
0.2
0.1
92
94
96
98
100
Oxygen Saturation

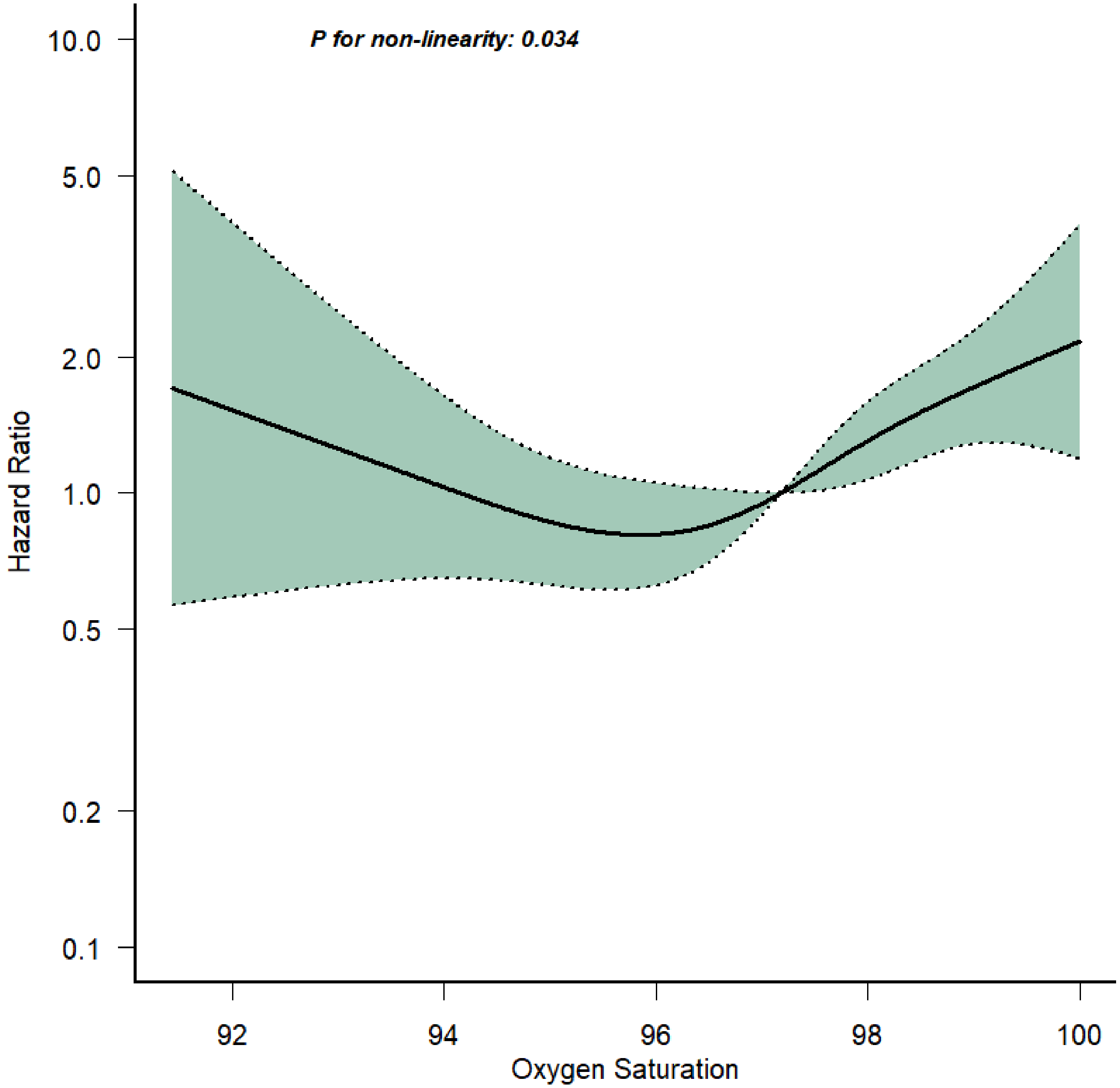
P for non-linearity: 0.034
10.0
5.0
2.0
1.0
0.5
0.2
0.1
Hazard Ratio
92
94
96
98
100
Oxygen Saturation

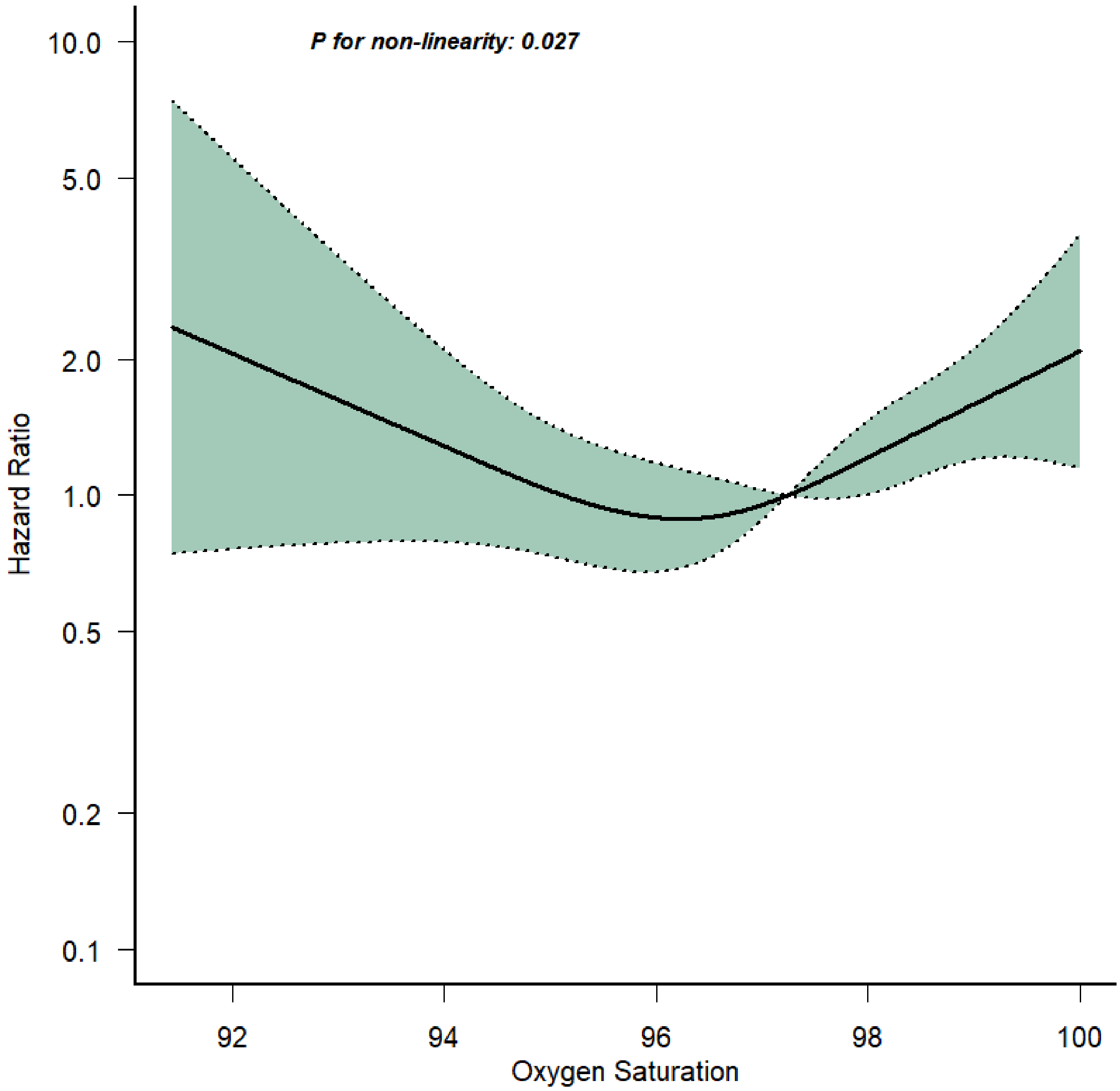
P for non-linearity: 0.027
10.0
5.0
2.0
1.0
0.5
0.2
0.1
Hazard Ratio
92
94
96
98
100
Oxygen Saturation

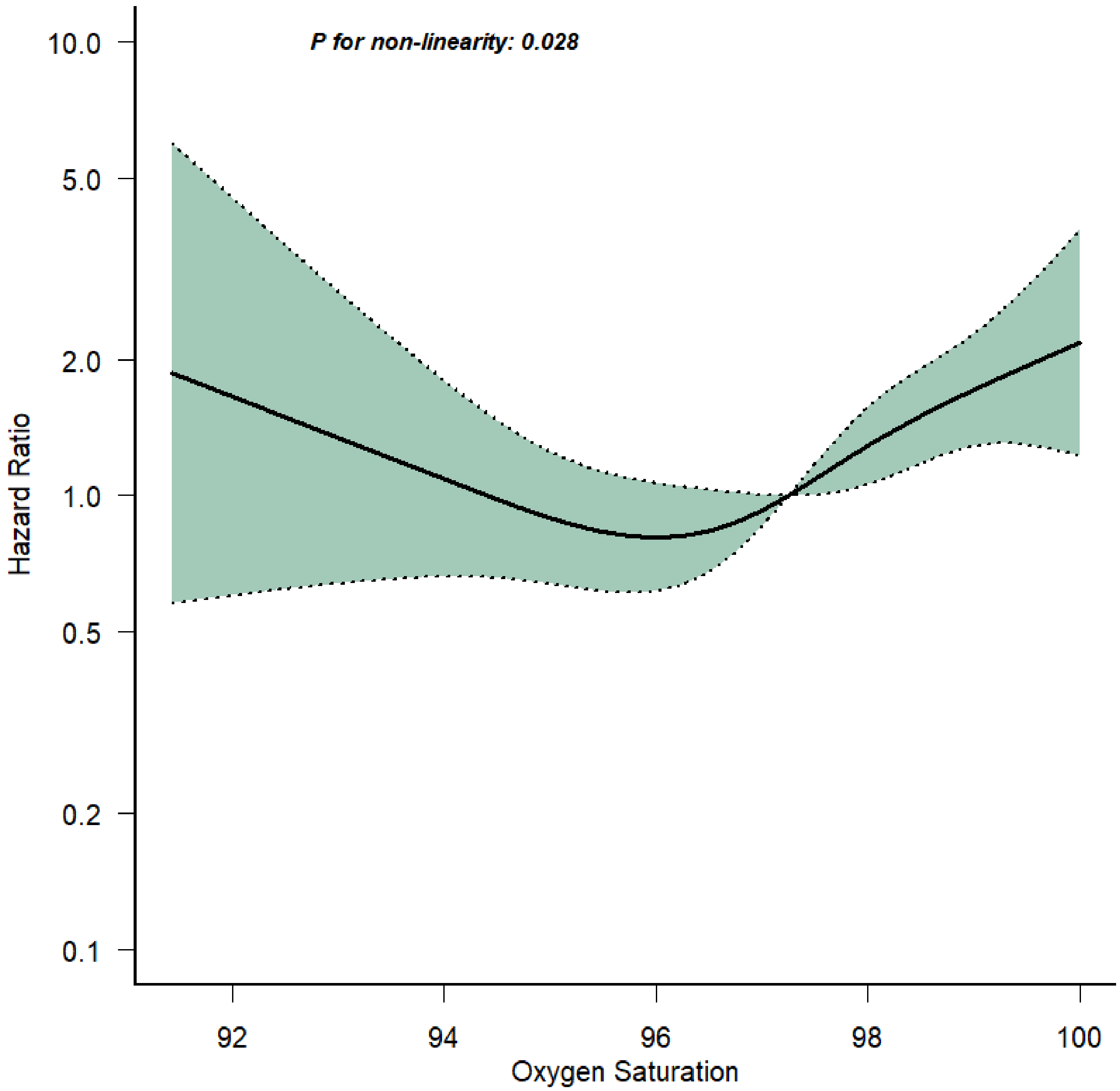
P for non-linearity: 0.028
10.0
5.0
2.0
1.0
0.5
0.2
0.1
Hazard Ratio
92
94
96
98
100
Oxygen Saturation

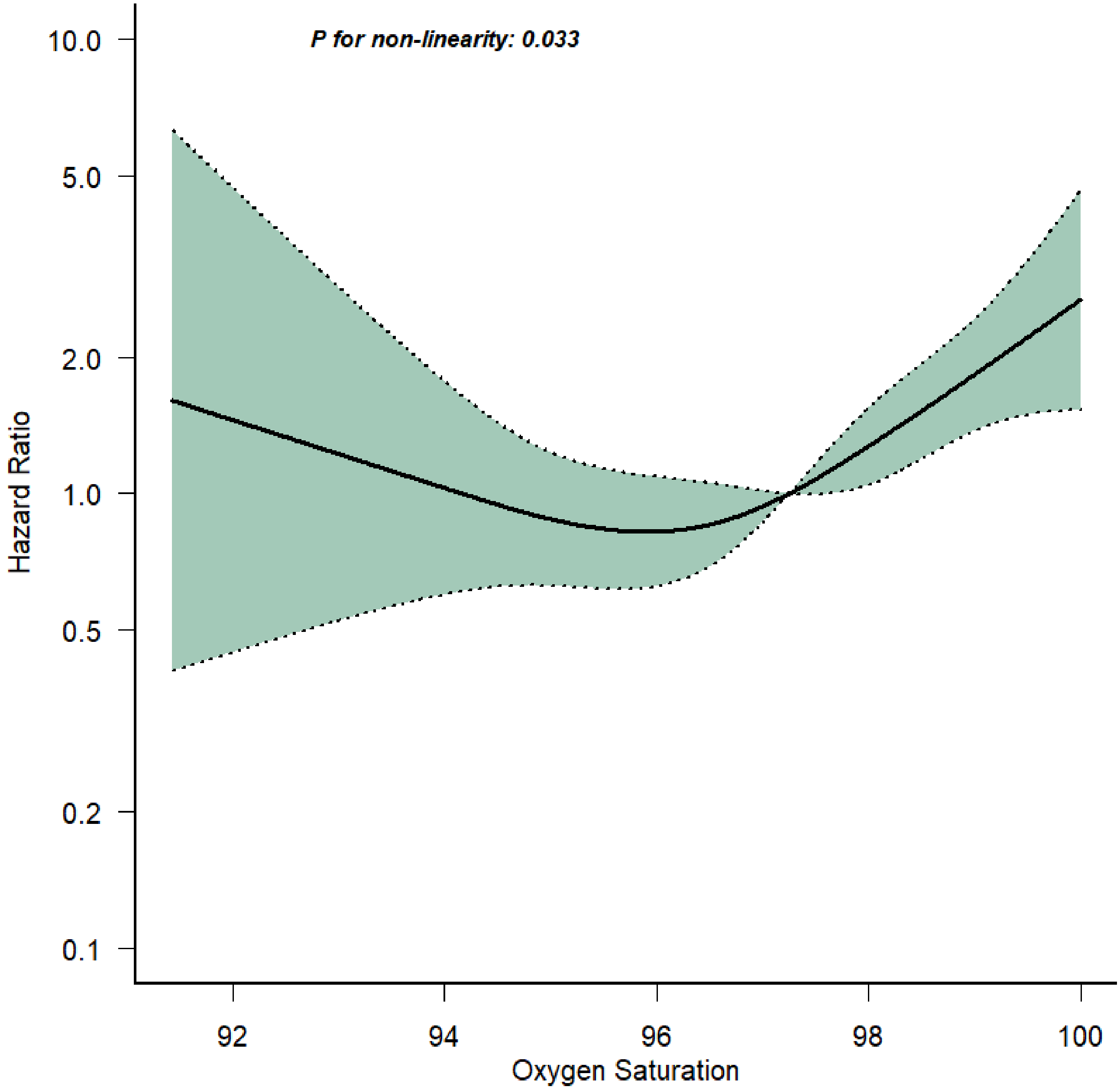
P for non-linearity: 0.033
10.0
5.0
2.0
1.0
0.5
0.2
0.1
Hazard Ratio
92
94
96
98
100
Oxygen Saturation

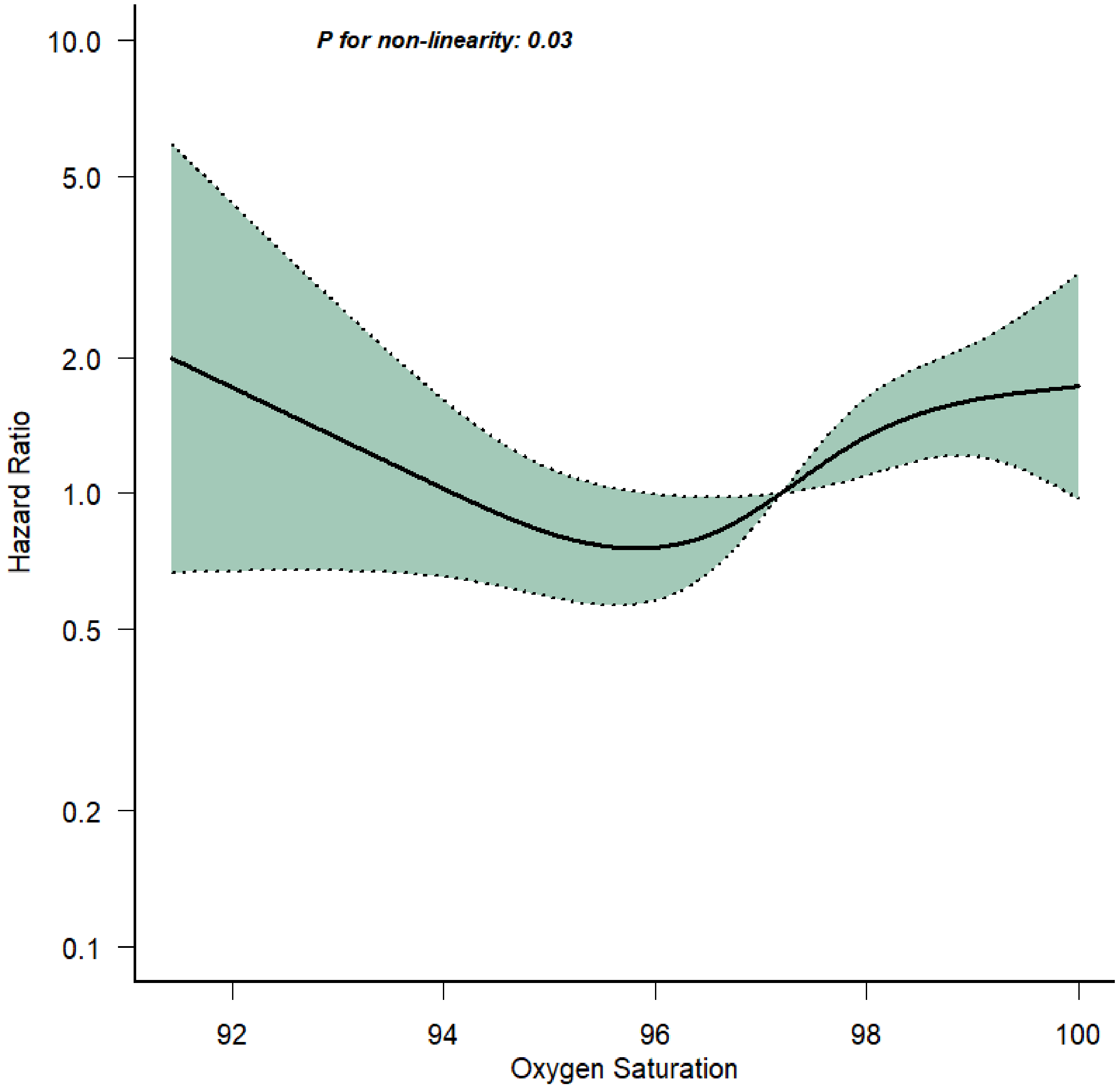
P for non-linearity: 0.03
10.0
5.0
2.0
1.0
0.5
0.2
0.1
Hazard Ratio
92
94
96
98
100
Oxygen Saturation

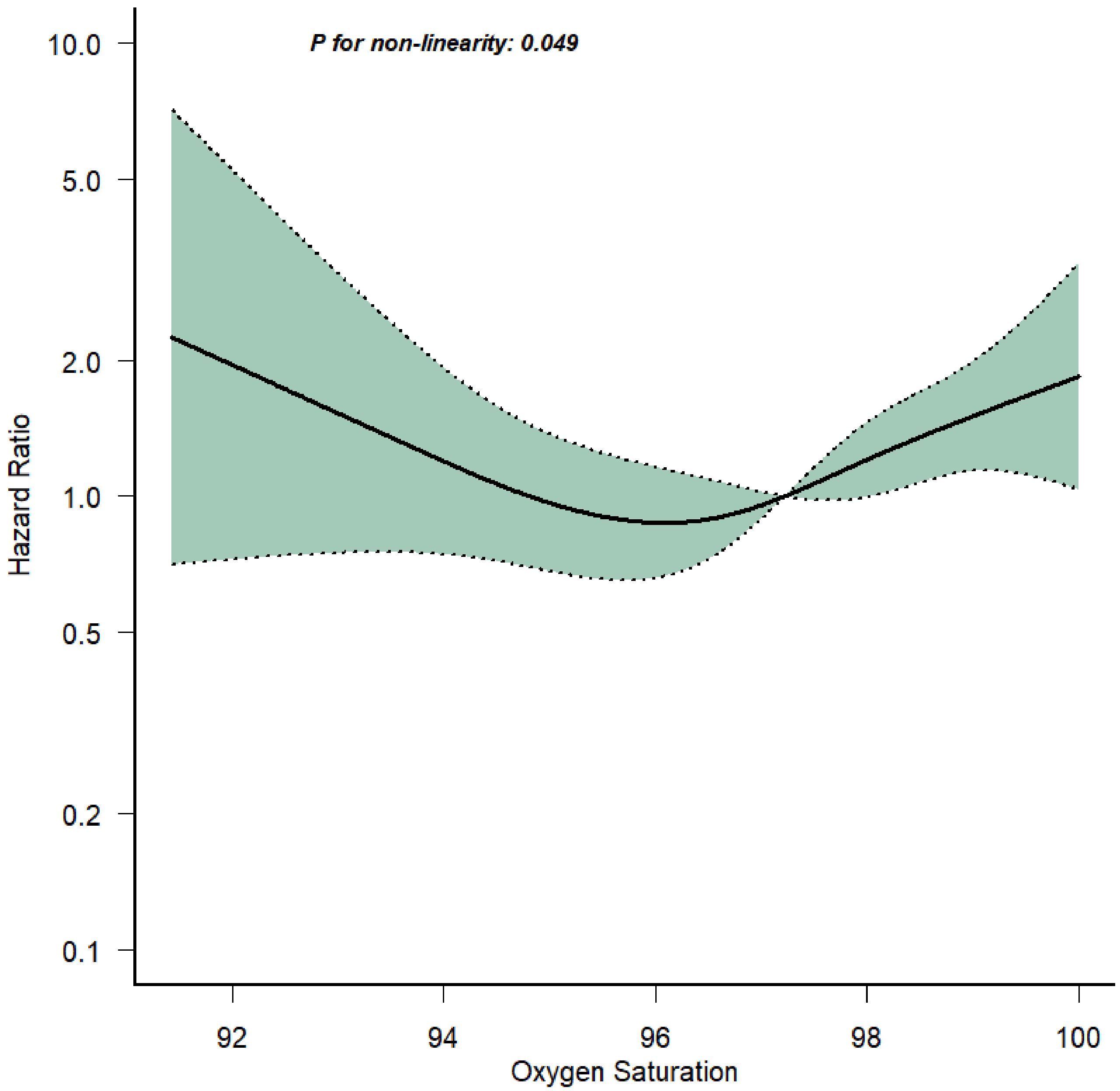
P for non-linearity: 0.049
10.0
5.0
2.0
1.0
0.5
0.2
0.1
Hazard Ratio
92
94
96
98
100
Oxygen Saturation

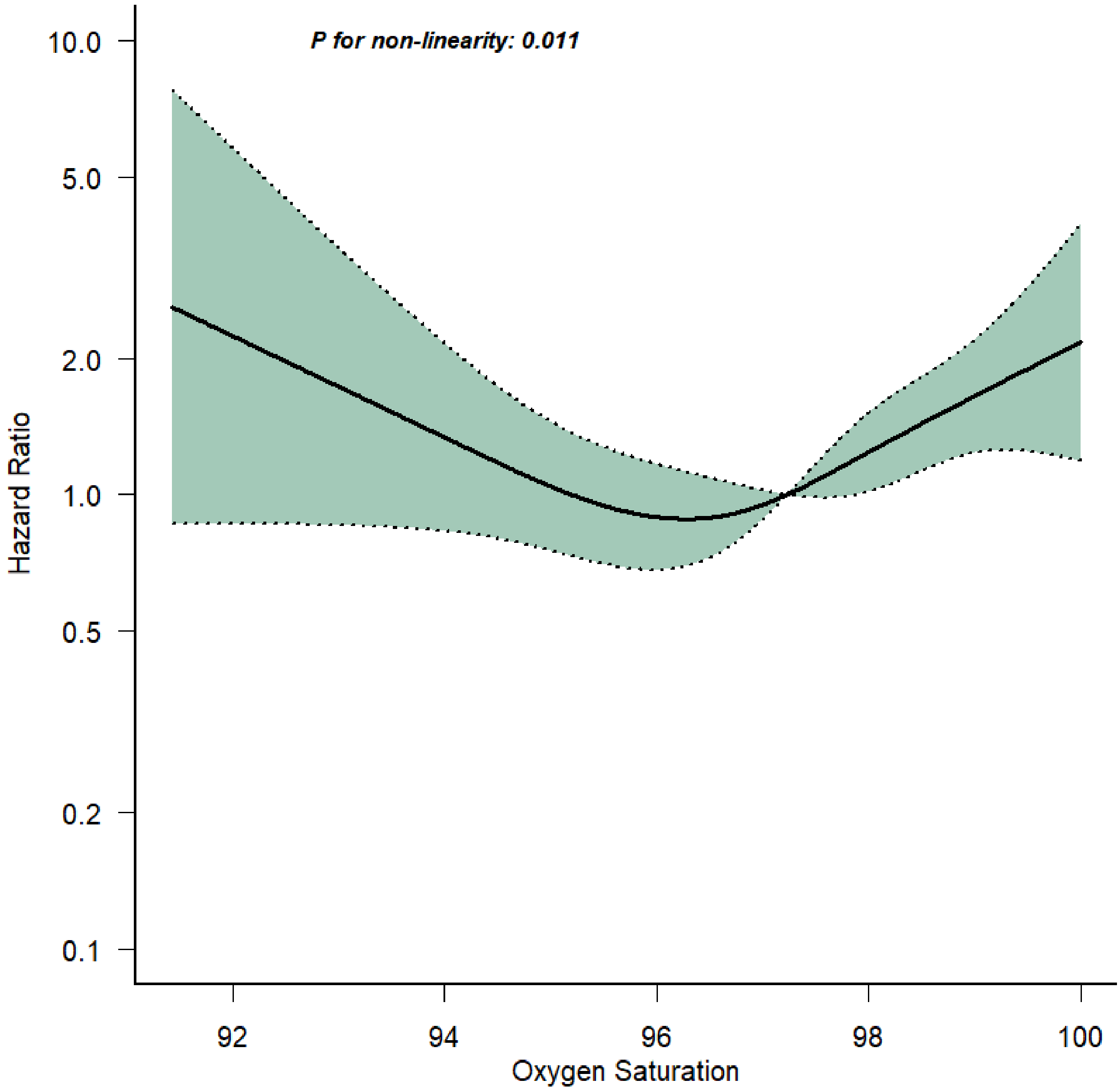
P for non-linearity: 0.011
10.0
5.0
2.0
1.0
0.5
0.2
0.1
Hazard Ratio
92
94
96
98
100
Oxygen Saturation

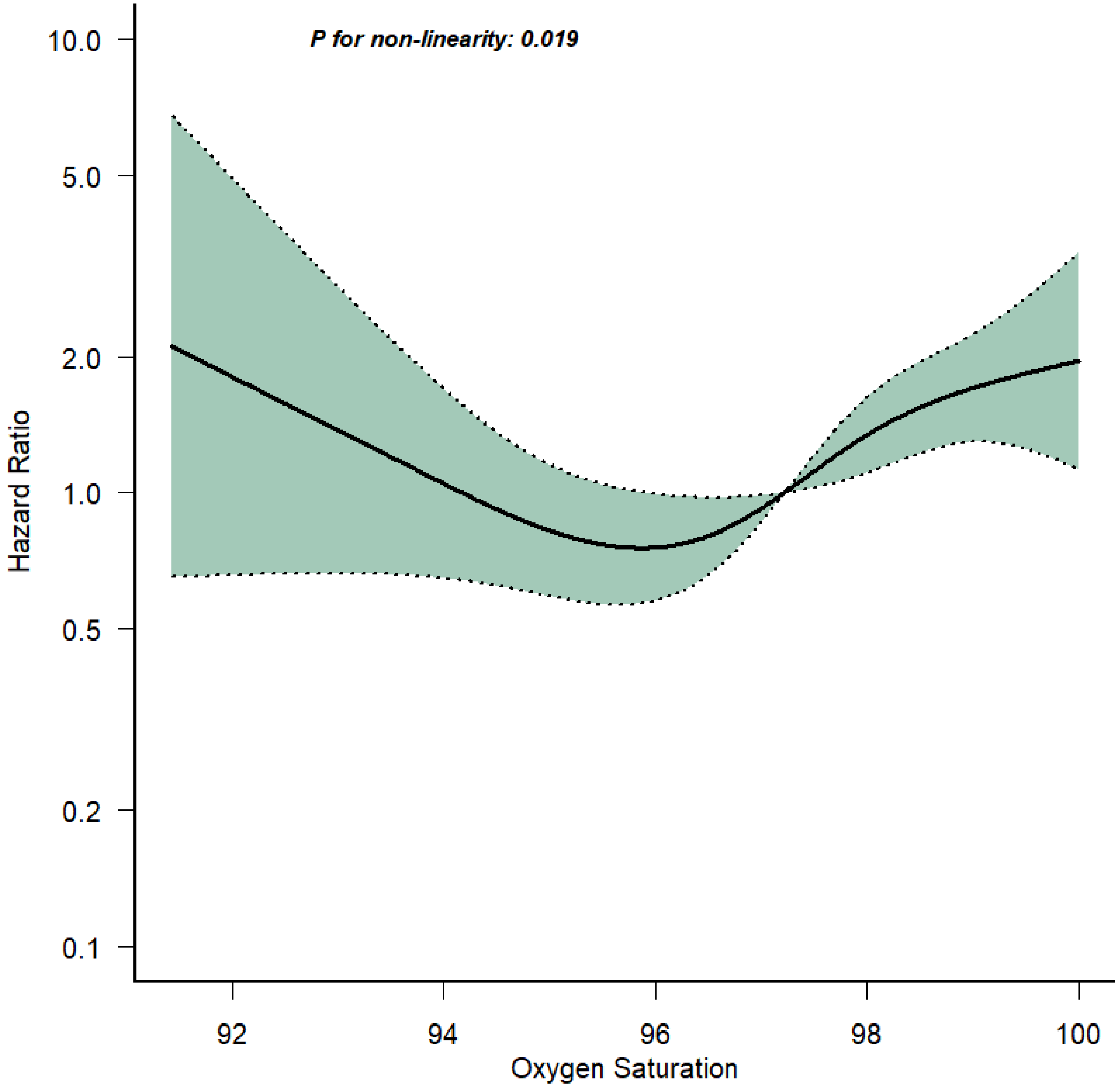
P for non-linearity: 0.019
Hazard Ratio
10.0
5.0
2.0
1.0
0.5
0.2
0.1
92
94
96
98
100
Oxygen Saturation

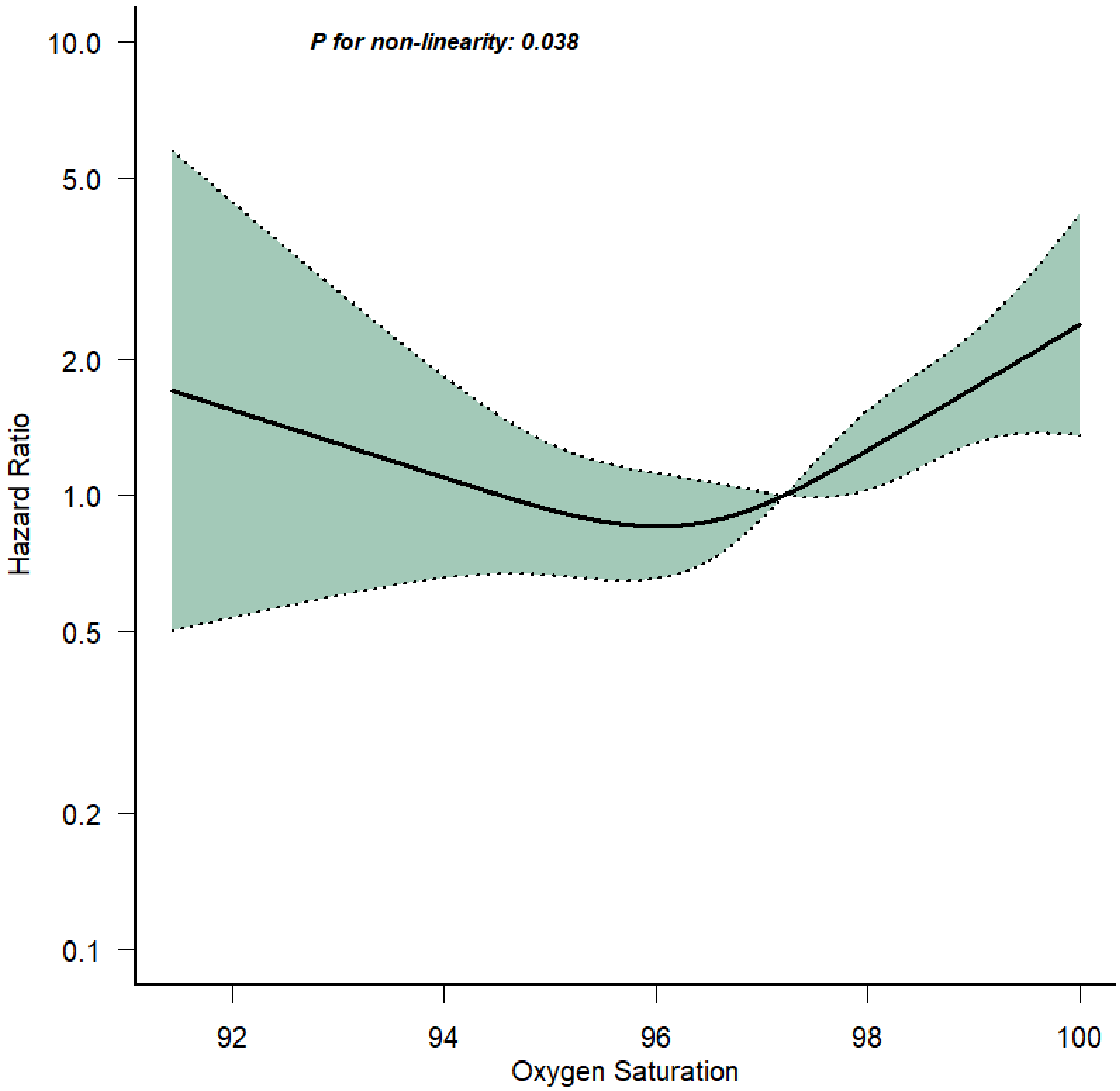
P for non-linearity: 0.038
10.0
5.0
2.0
1.0
0.5
0.2
0.1
Hazard Ratio
92
94
96
98
100
Oxygen Saturation

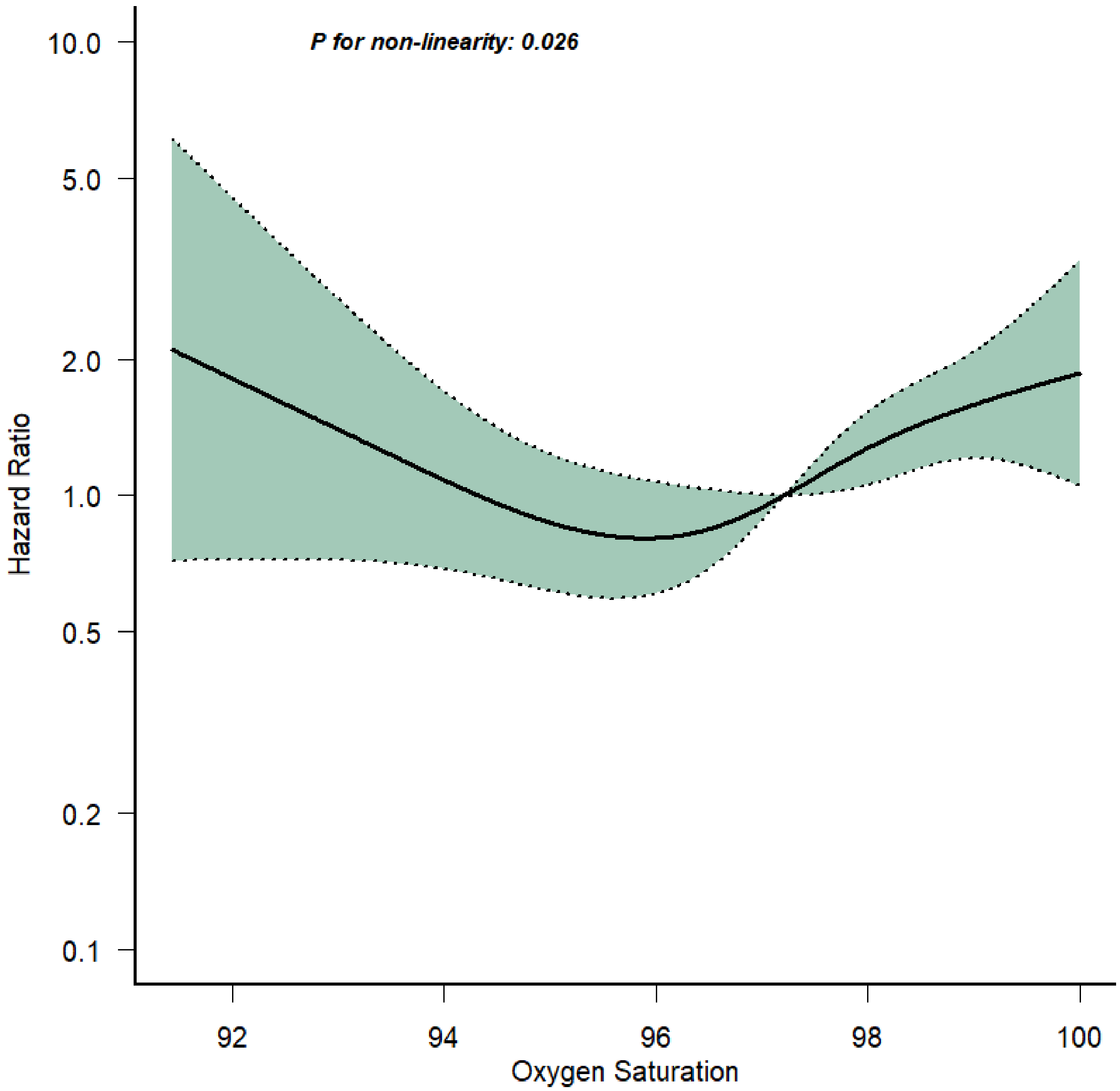
P for non-linearity: 0.026
10.0
5.0
2.0
1.0
0.5
0.2
0.1
Hazard Ratio
92
94
96
98
100
Oxygen Saturation

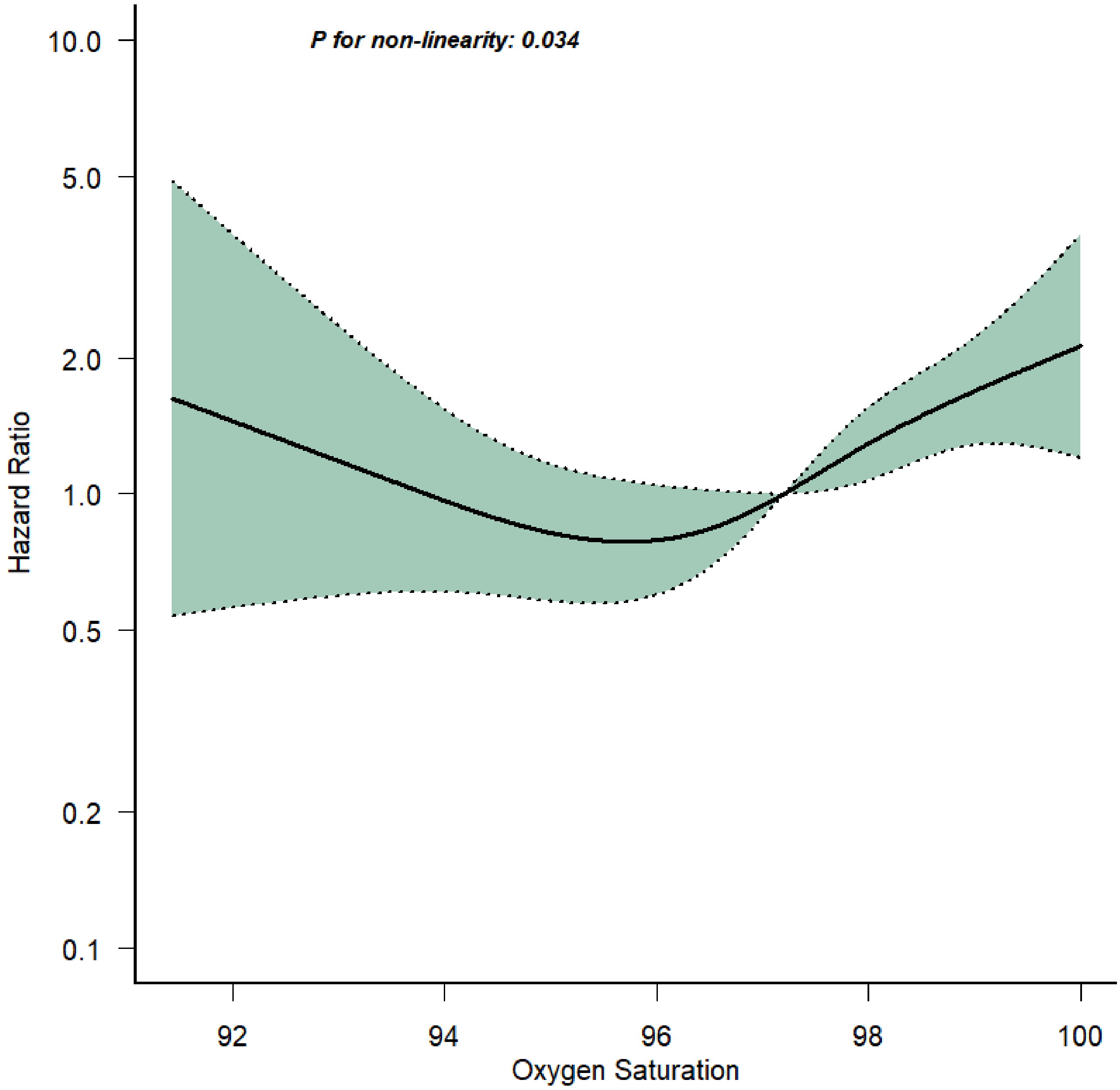

P for non-linearity: 0.034
Hazard Ratio
10.0
5.0
2.0
1.0
0.5
0.2
0.1
92
94
96
98
100
Oxygen Saturation

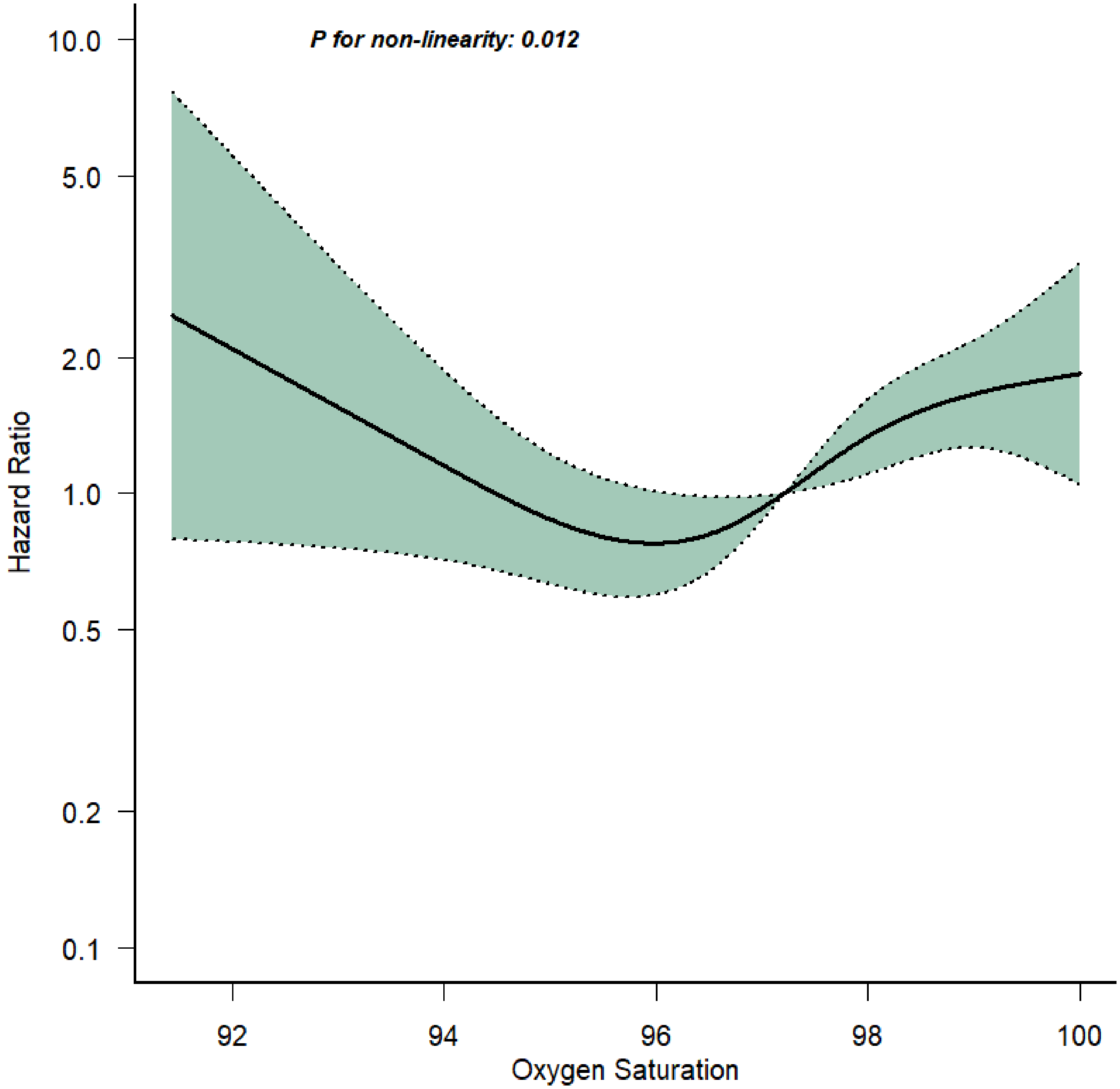
P for non-linearity: 0.012
Hazard Ratio
10.0
5.0
2.0
1.0
0.5
0.2
0.1
92
94
96
98
100
Oxygen Saturation

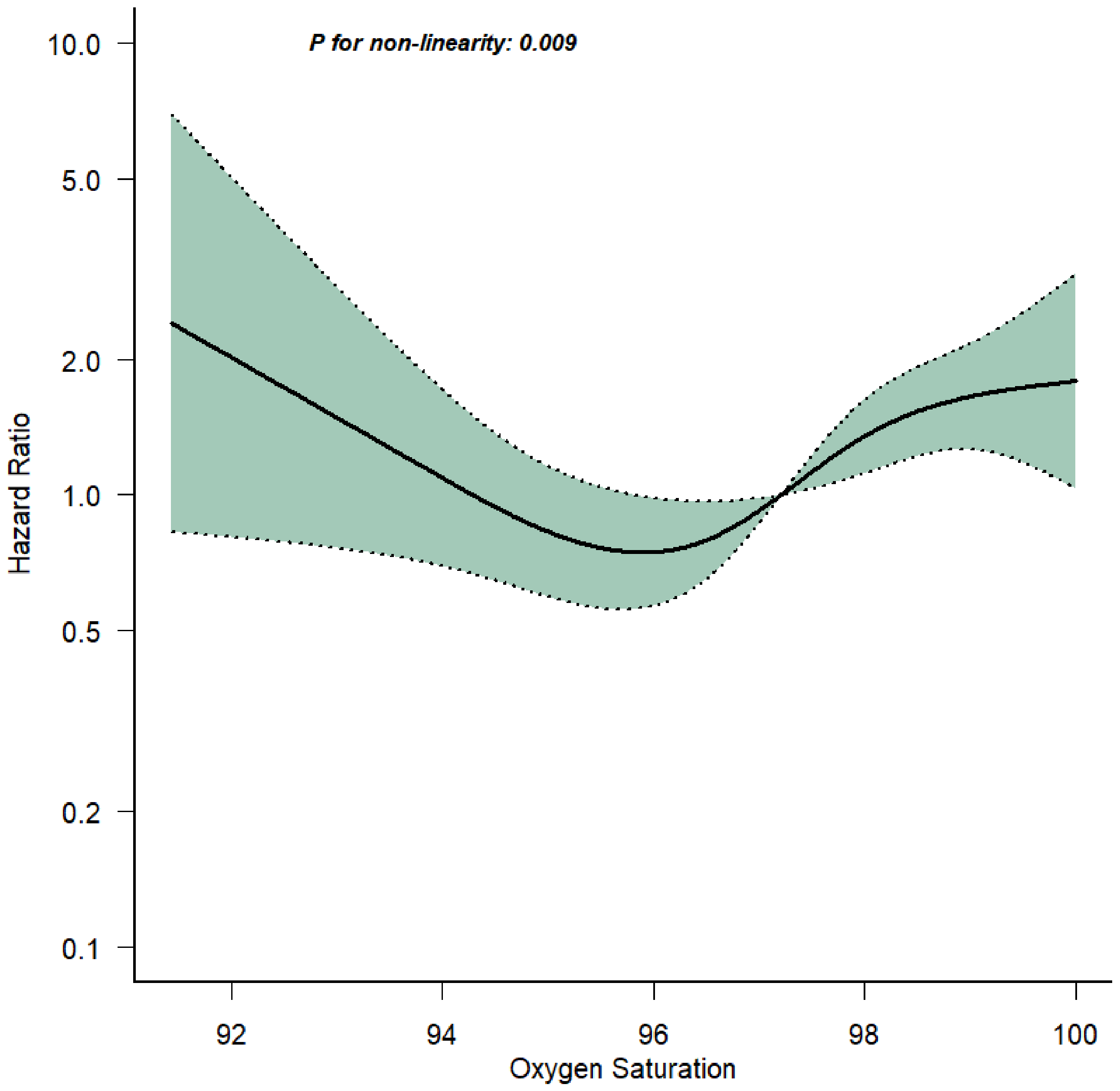
P for non-linearity: 0.009
10.0
5.0
2.0
1.0
0.5
0.2
0.1
Hazard Ratio
92
94
96
98
100
Oxygen Saturation

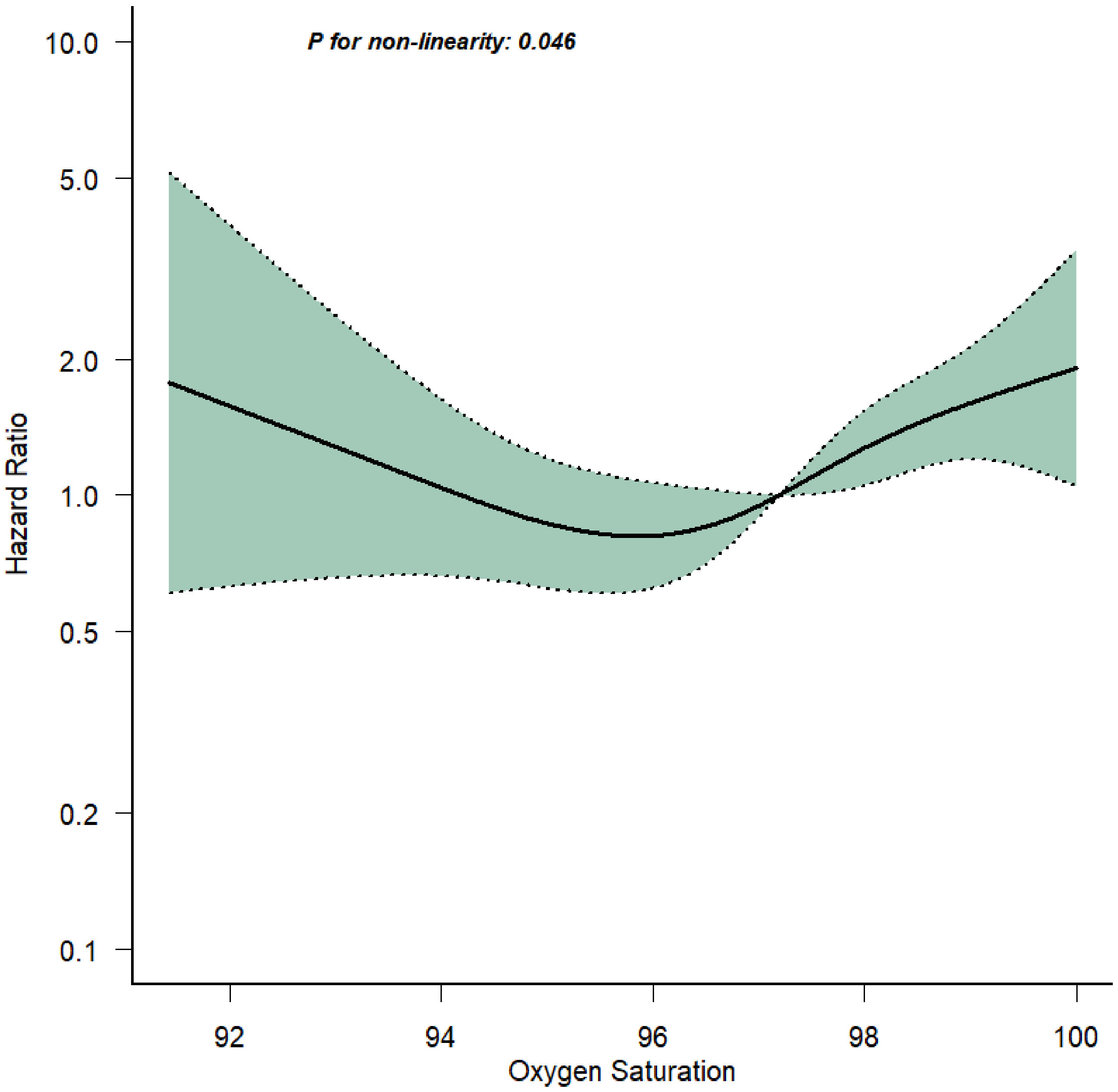
P for non-linearity: 0.046
10.0
5.0
2.0
1.0
0.5
0.2
0.1
Hazard Ratio
92
94
96
98
100
Oxygen Saturation

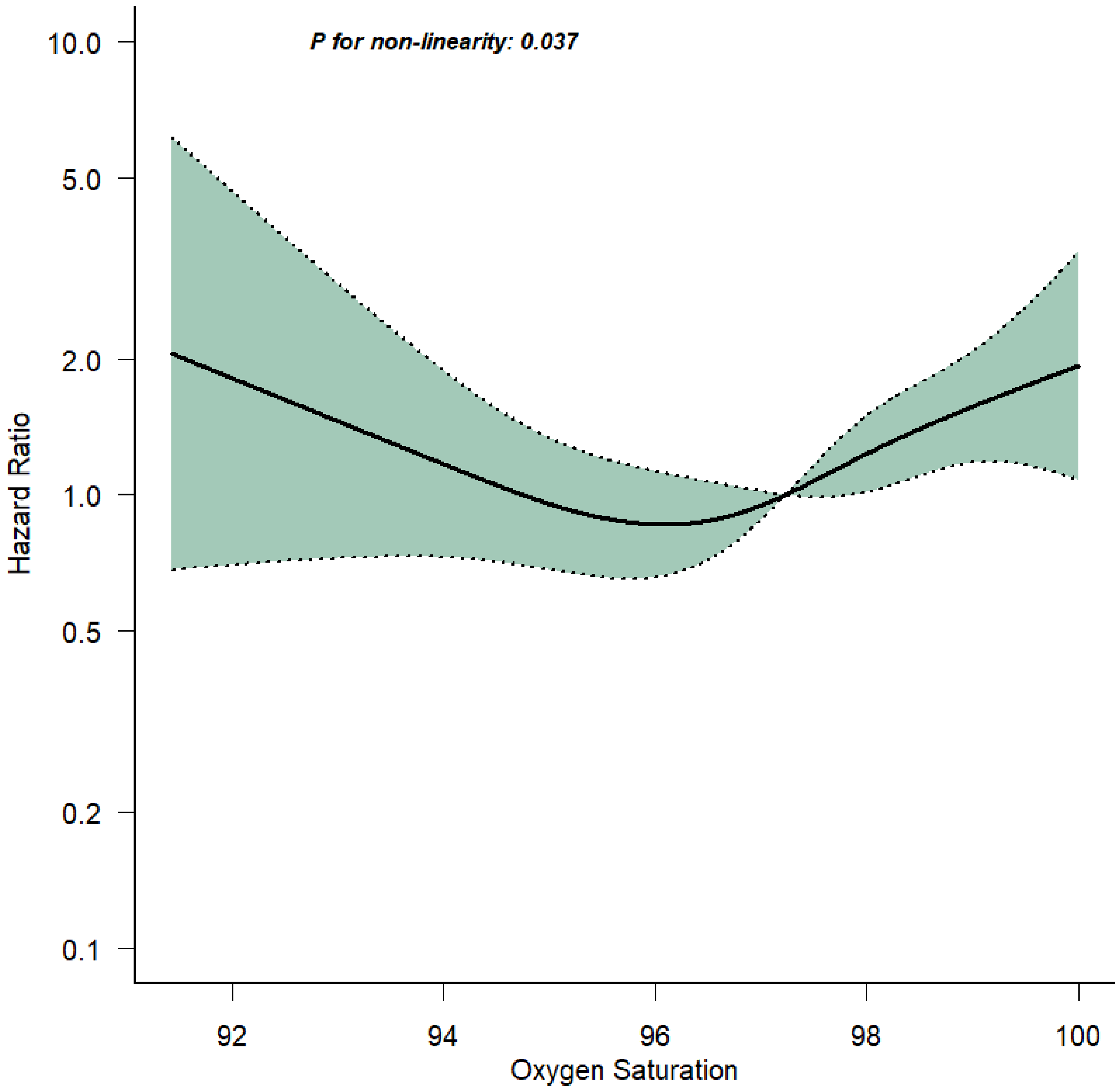
P for non-linearity: 0.037
10.0
5.0
2.0
1.0
0.5
0.2
0.1
Hazard Ratio
92
94
96
98
100
Oxygen Saturation

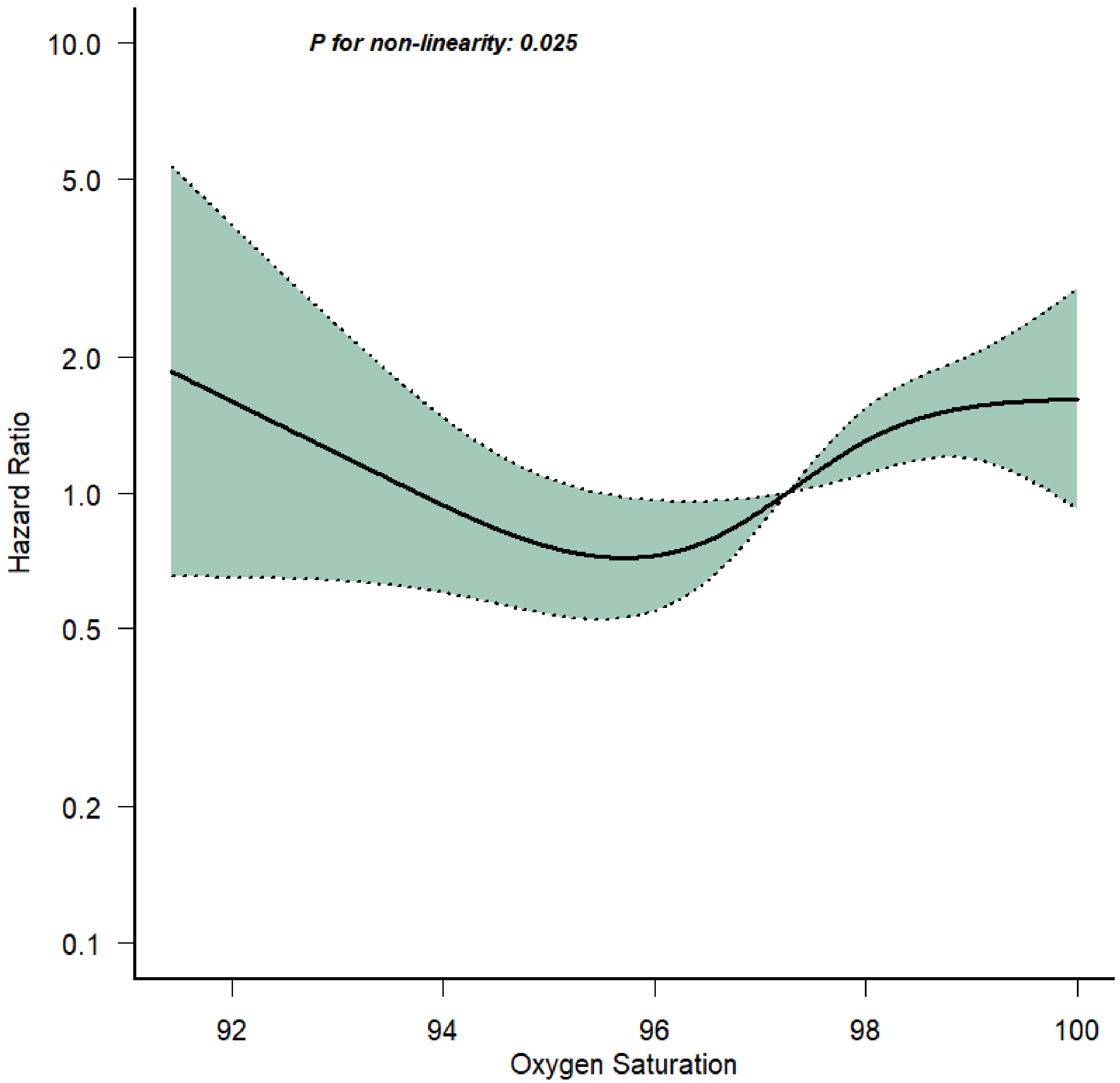
P for non-linearity: 0.025
Hazard Ratio
10.0
5.0
2.0
1.0
0.5
0.2
0.1
92
94
96
98
100
Oxygen Saturation

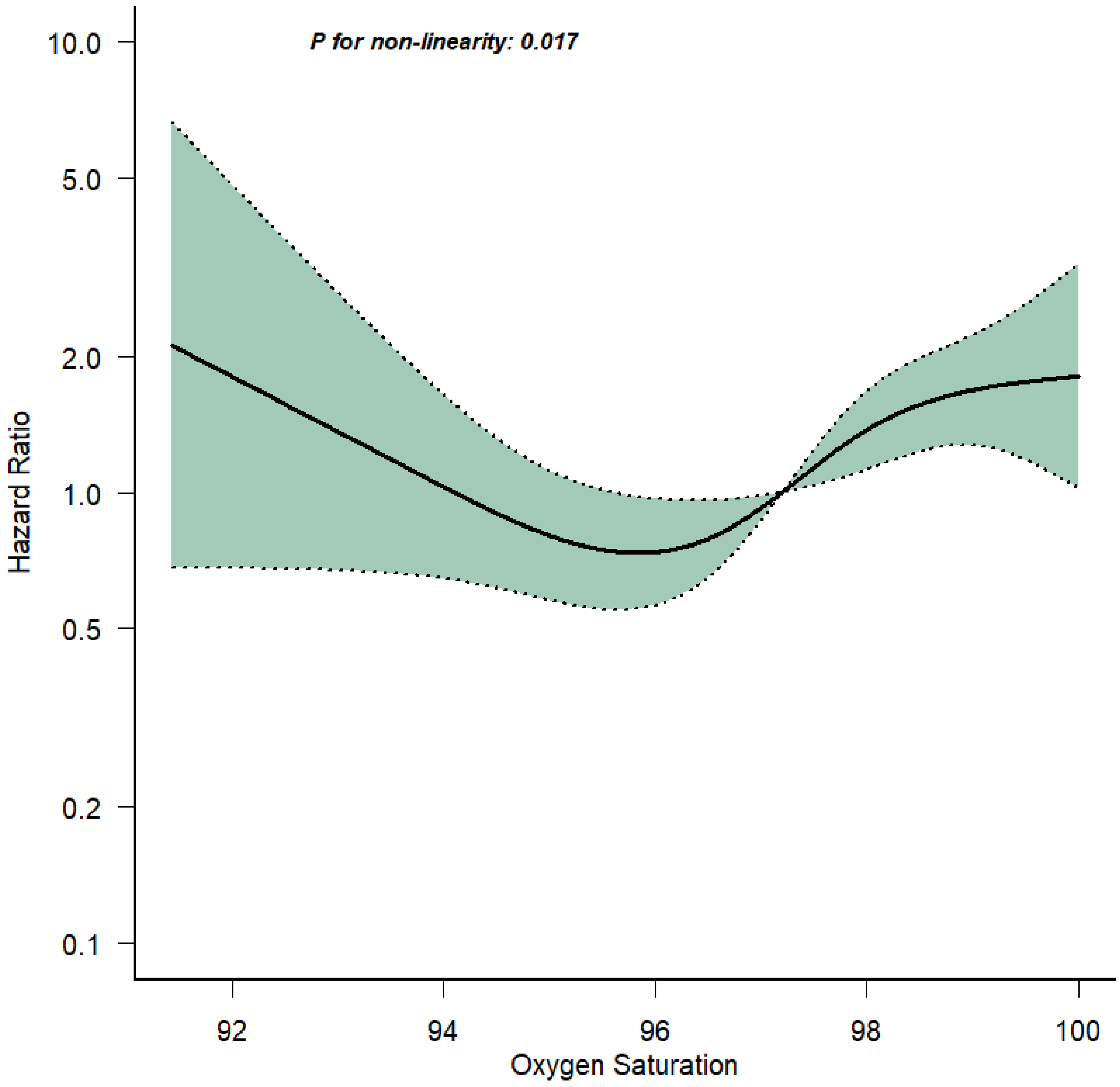
P for non-linearity: 0.017
10.0
5.0
2.0
1.0
0.5
0.2
0.1
Hazard Ratio
92
94
96
98
100
Oxygen Saturation

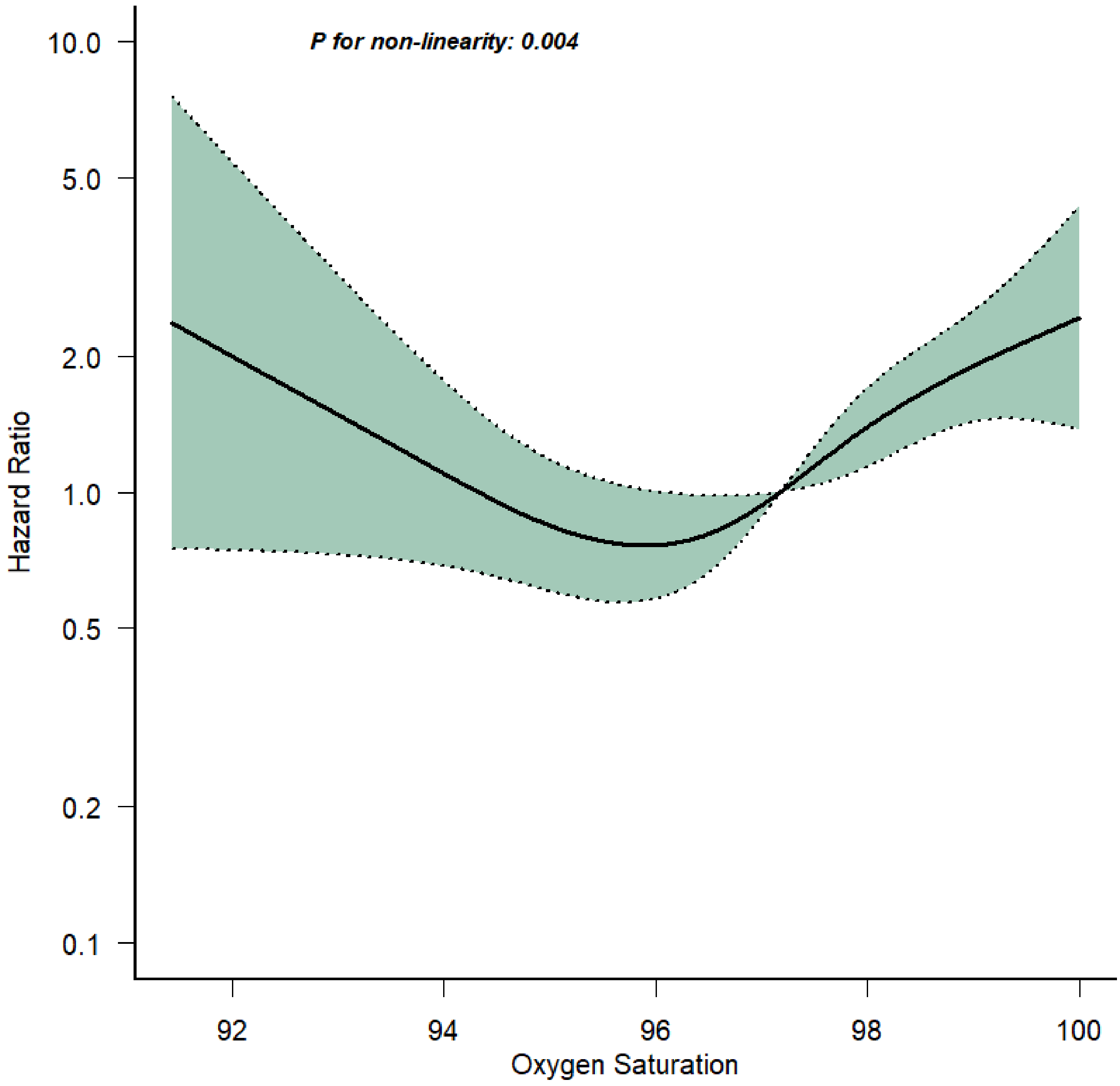
P for non-linearity: 0.004
10.0
5.0
2.0
1.0
0.5
0.2
0.1
Hazard Ratio
92
94
96
98
100
Oxygen Saturation

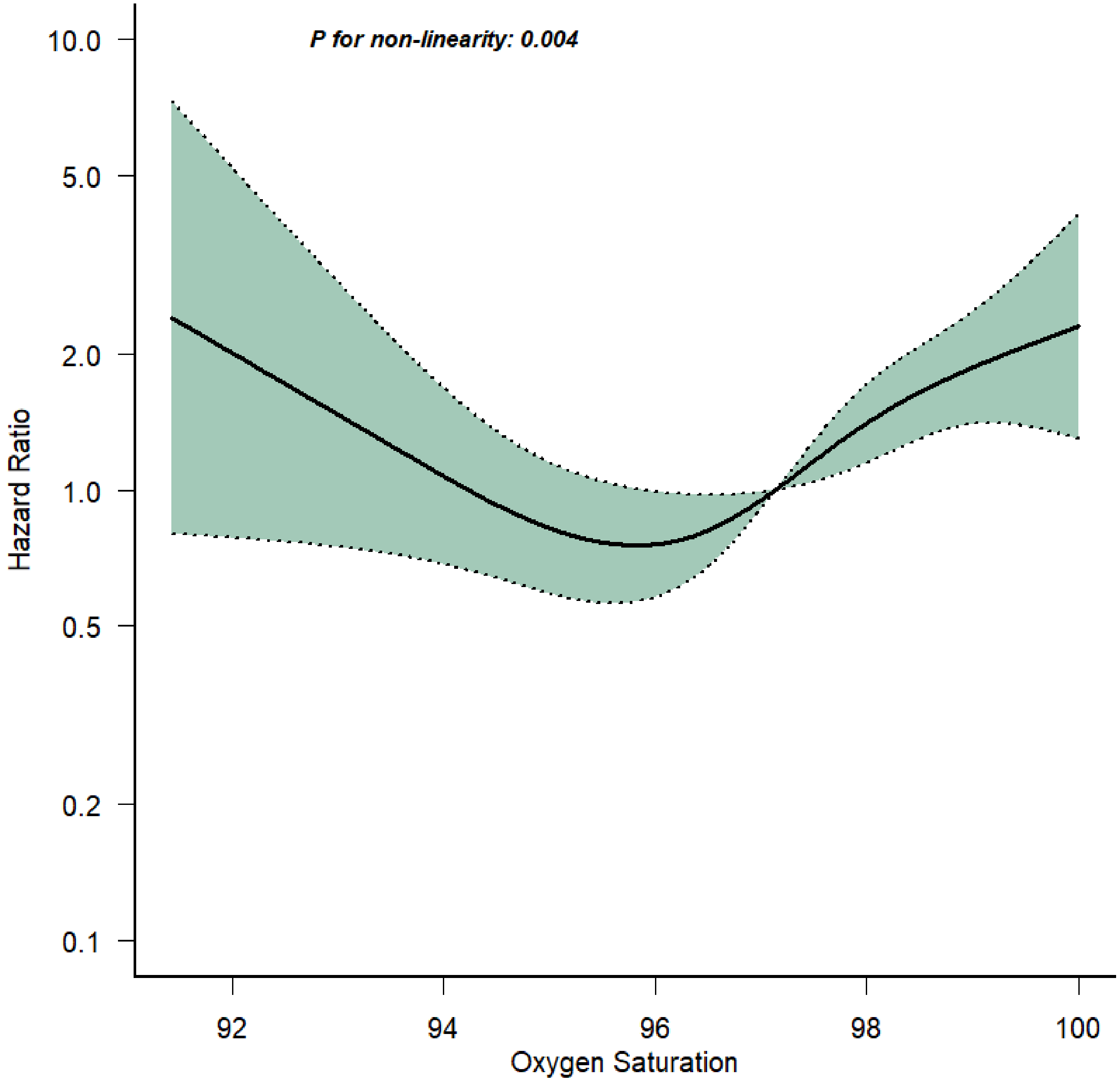
P for non-linearity: 0.004
10.0
5.0
2.0
1.0
0.5
0.2
0.1
Hazard Ratio
92
94
96
98
100
Oxygen Saturation

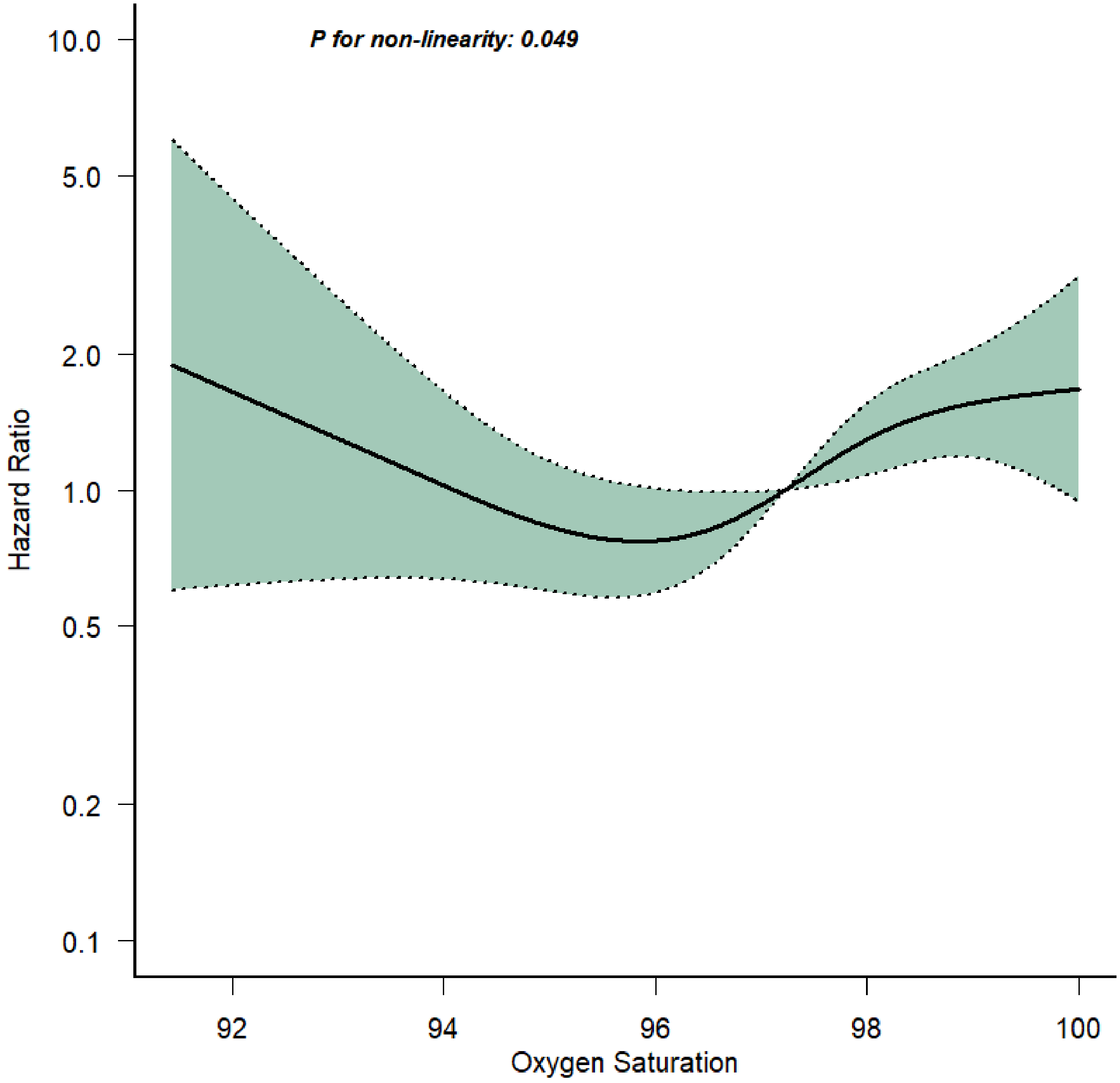
P for non-linearity: 0.049
10.0
5.0
2.0
1.0
0.5
0.2
0.1
Hazard Ratio
92
94
96
98
100
Oxygen Saturation

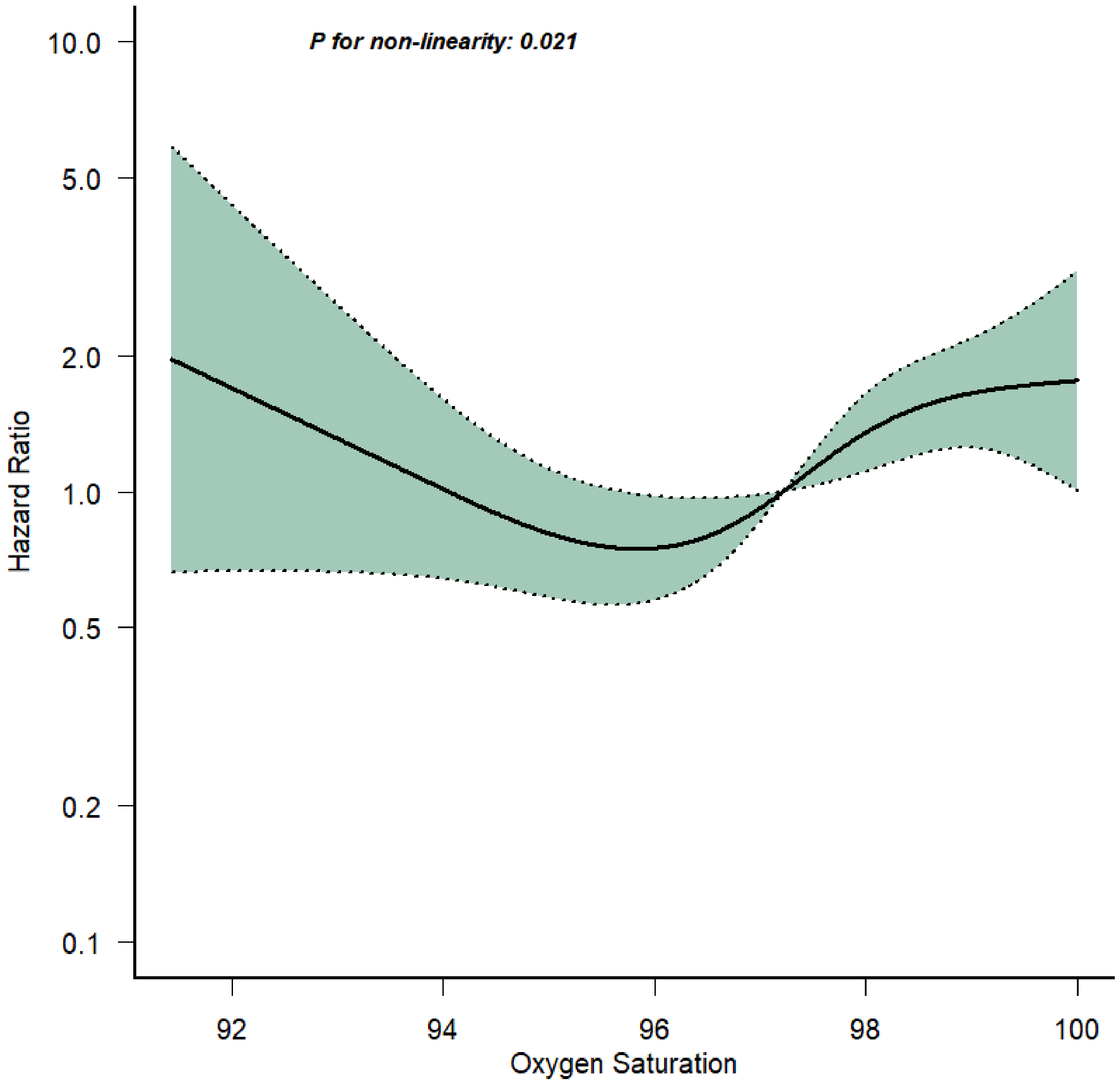
P for non-linearity: 0.021
10.0
5.0
2.0
1.0
0.5
0.2
0.1
Hazard Ratio
92
94
96
98
100
Oxygen Saturation

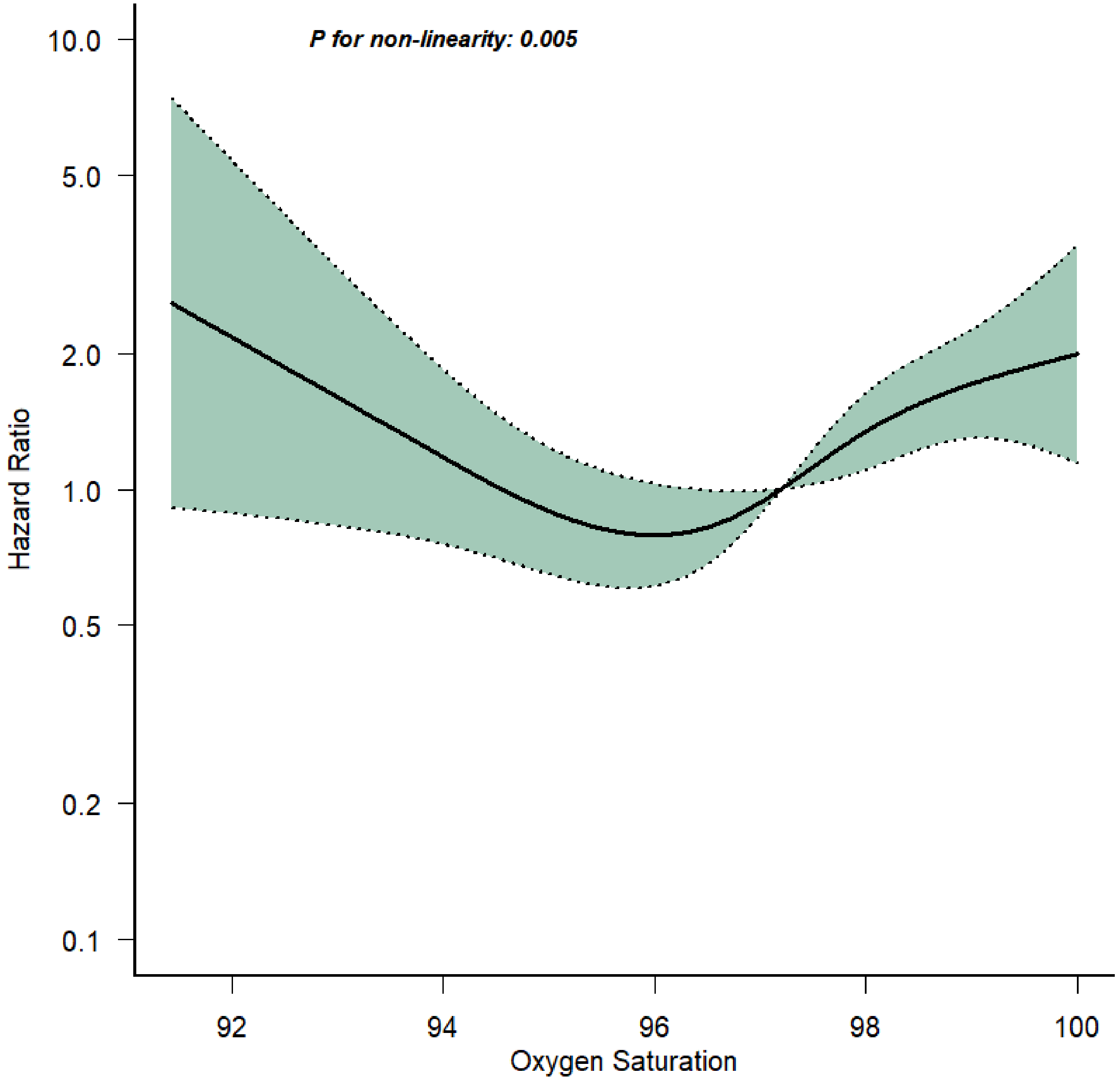
P for non-linearity: 0.005
Hazard Ratio
10.0
5.0
2.0
1.0
0.5
0.2
0.1
92
94
96
98
100
Oxygen Saturation

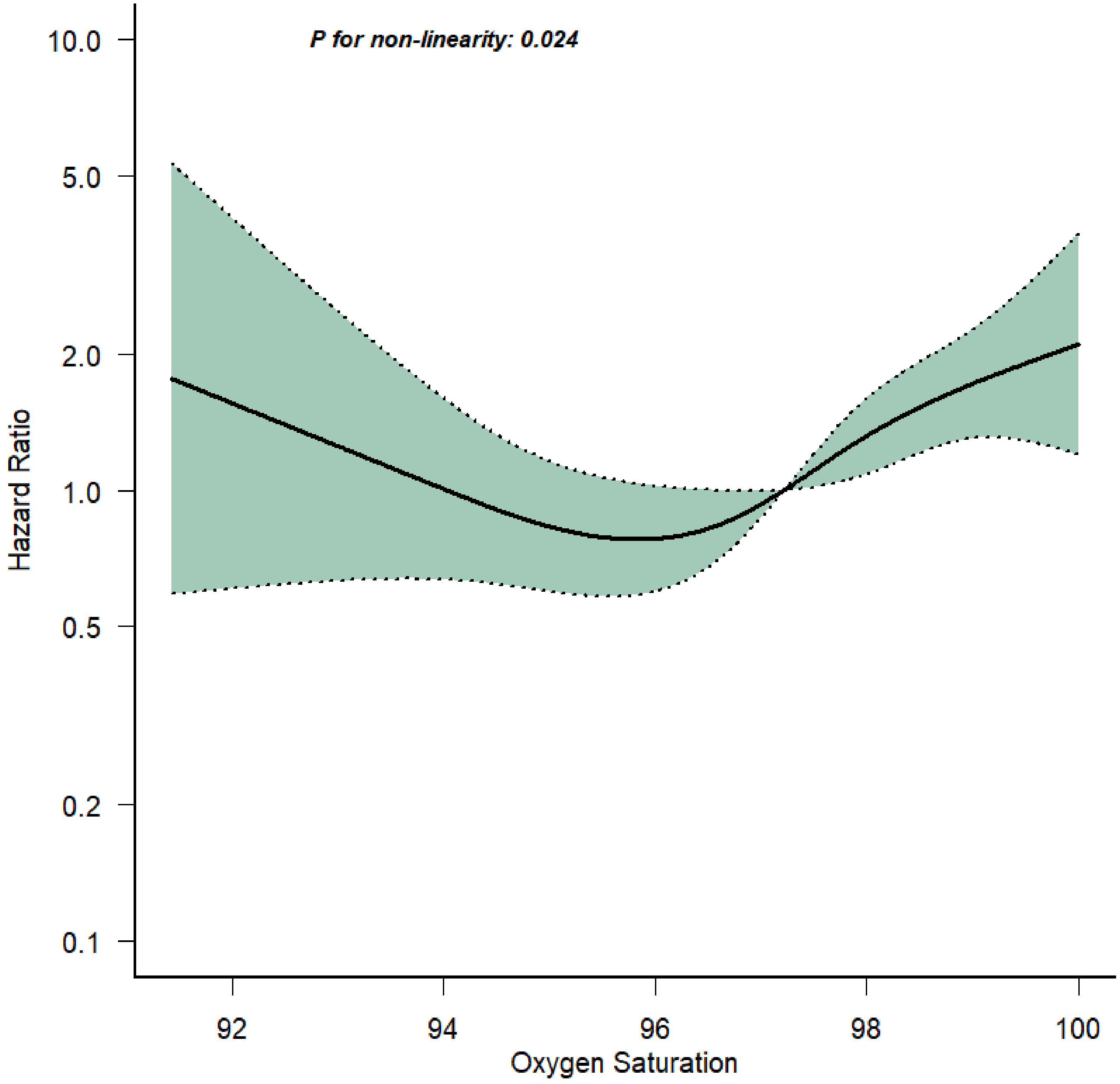

P for non-linearity: 0.024
10.0
5.0
2.0
1.0
0.5
0.2
0.1
Hazard Ratio
92
94
96
98
100
Oxygen Saturation

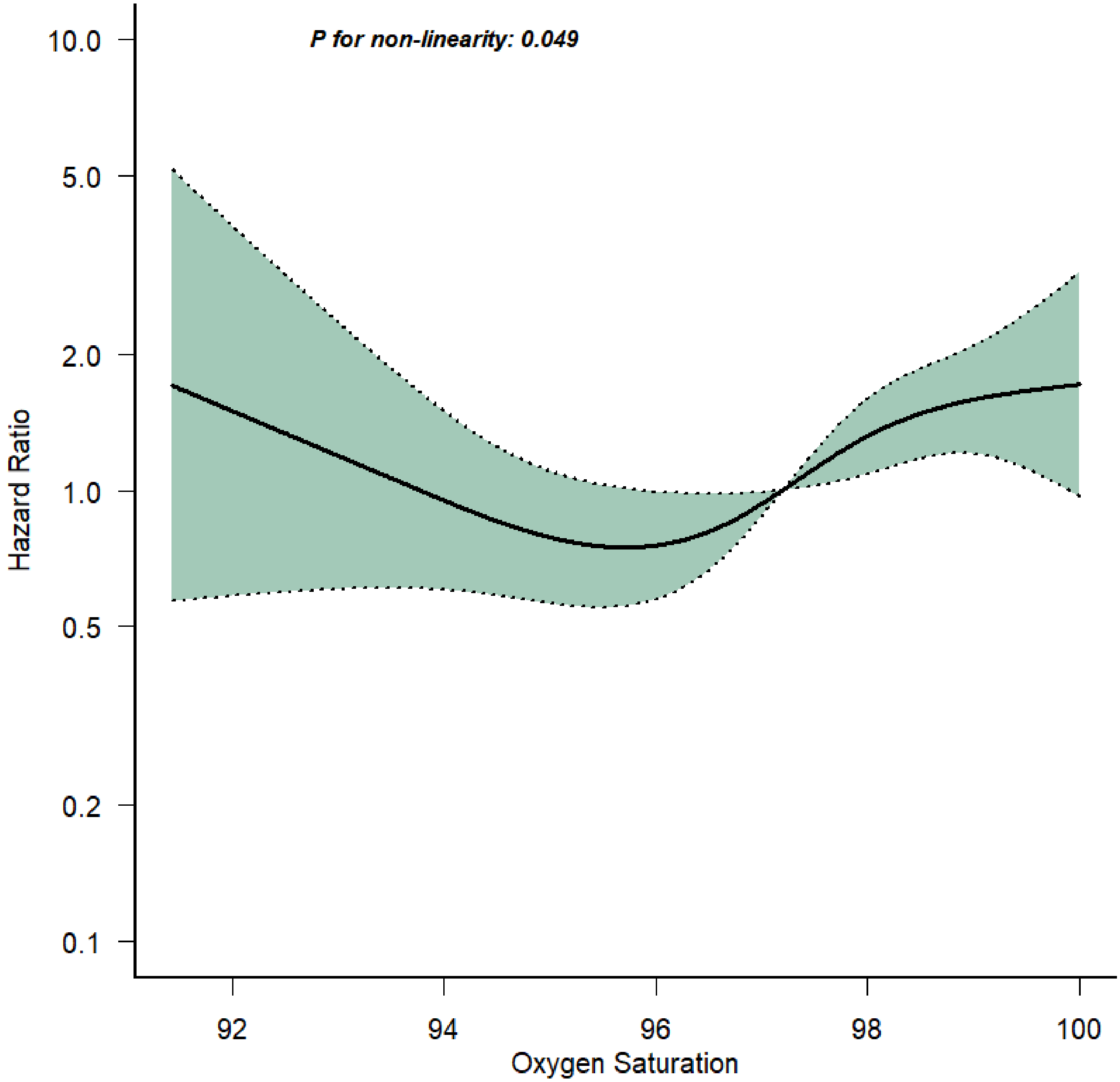
P for non-linearity: 0.049
10.0
5.0
2.0
1.0
0.5
0.2
0.1
Hazard Ratio
92
94
96
98
100
Oxygen Saturation

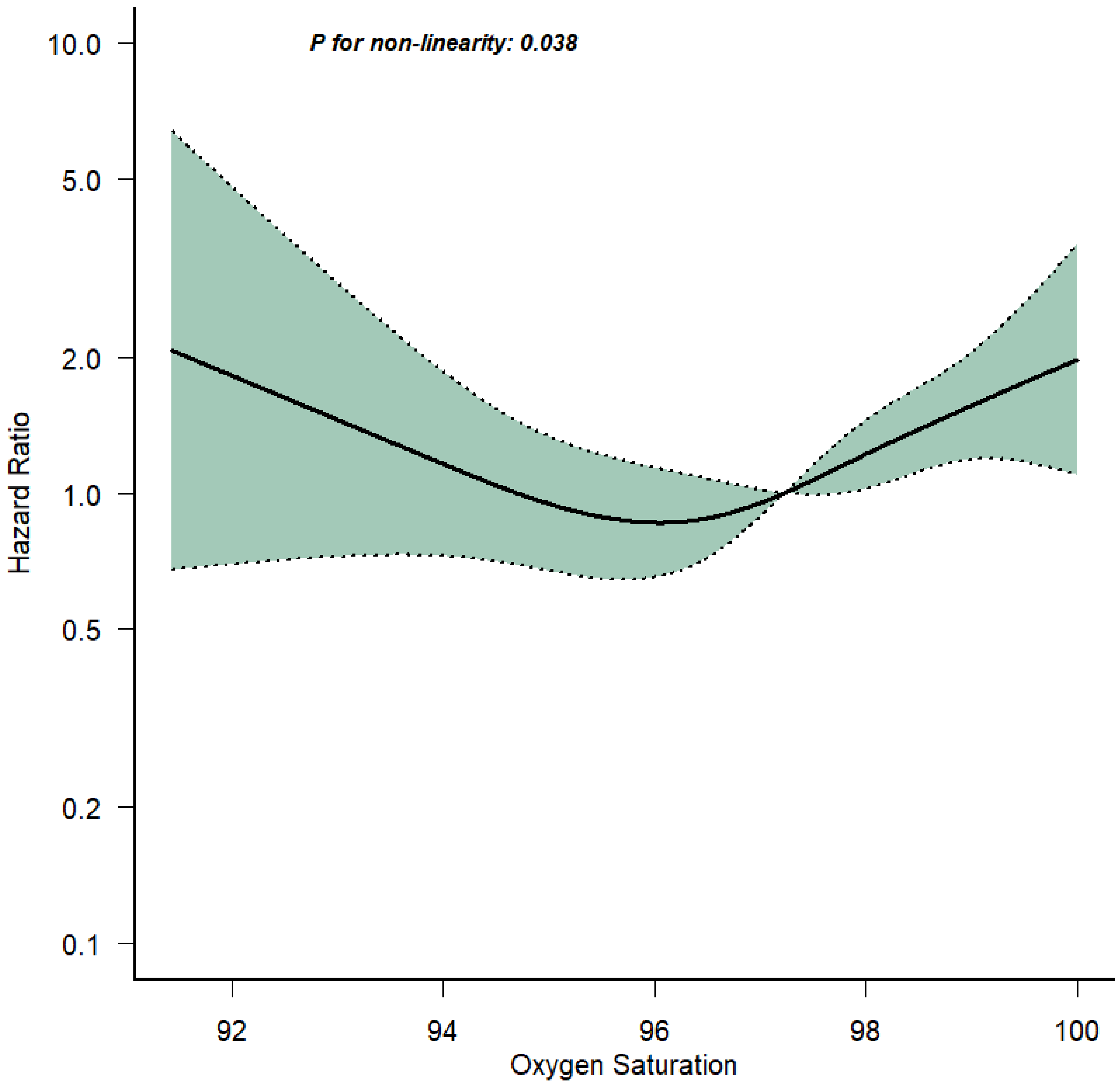

P for non-linearity: 0.038
Hazard Ratio
10.0
5.0
2.0
1.0
0.5
0.2
0.1
92
94
96
98
100
Oxygen Saturation

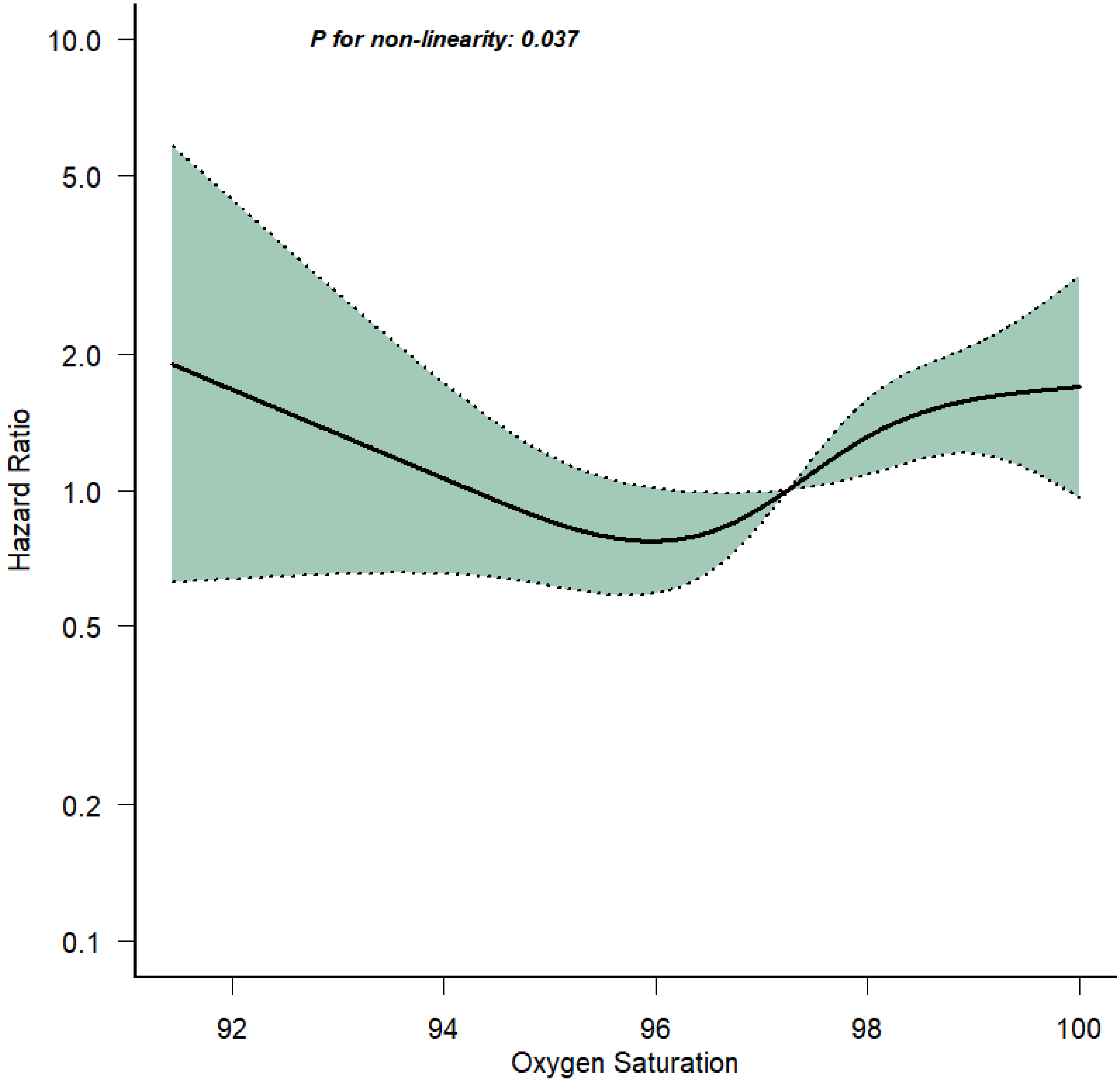
P for non-linearity: 0.037
10.0
5.0
2.0
1.0
0.5
0.2
0.1
Hazard Ratio
92
94
96
98
100
Oxygen Saturation

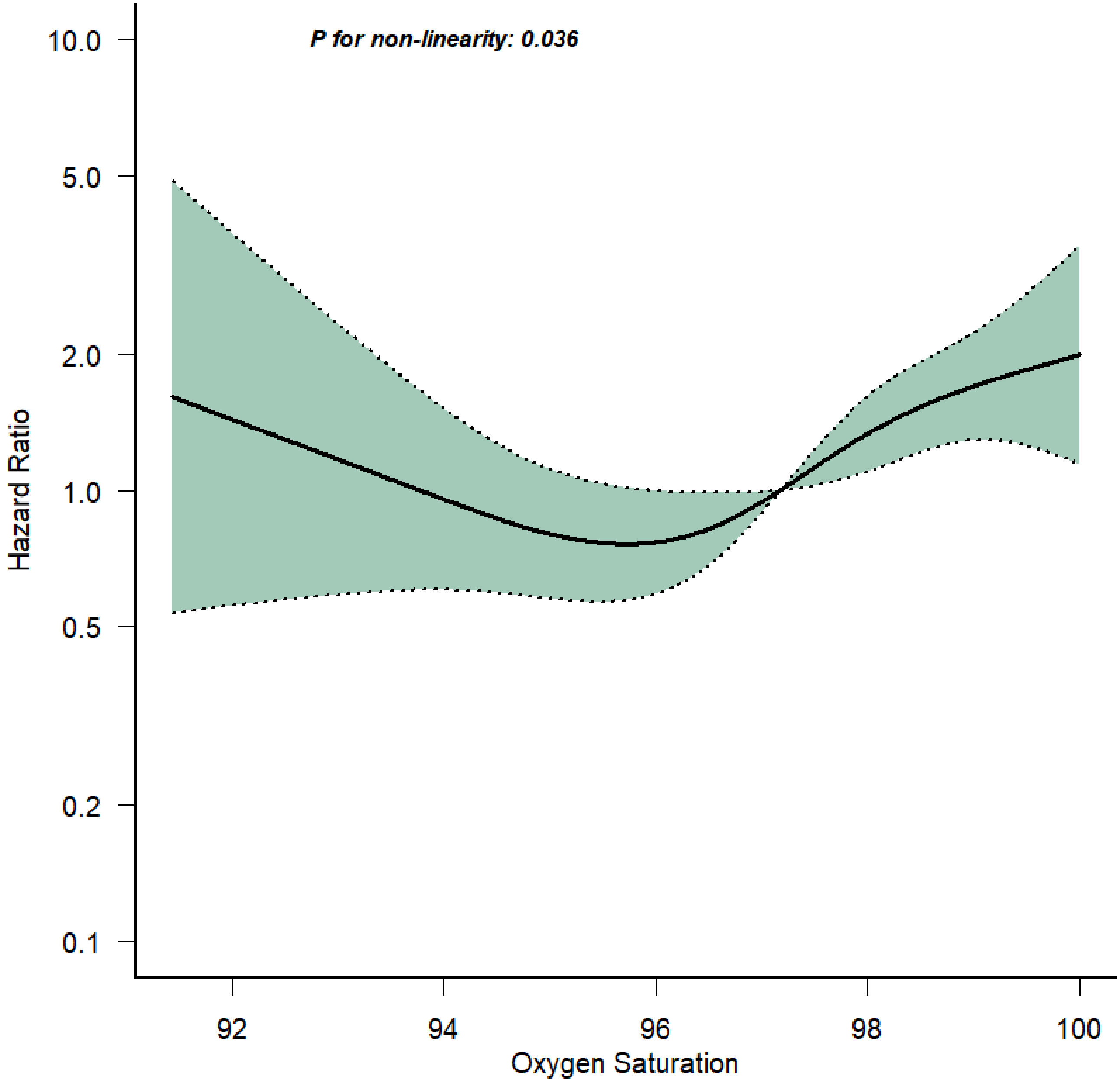
P for non-linearity: 0.036
10.0
5.0
2.0
1.0
0.5
0.2
0.1
Hazard Ratio
92
94
96
98
100
Oxygen Saturation

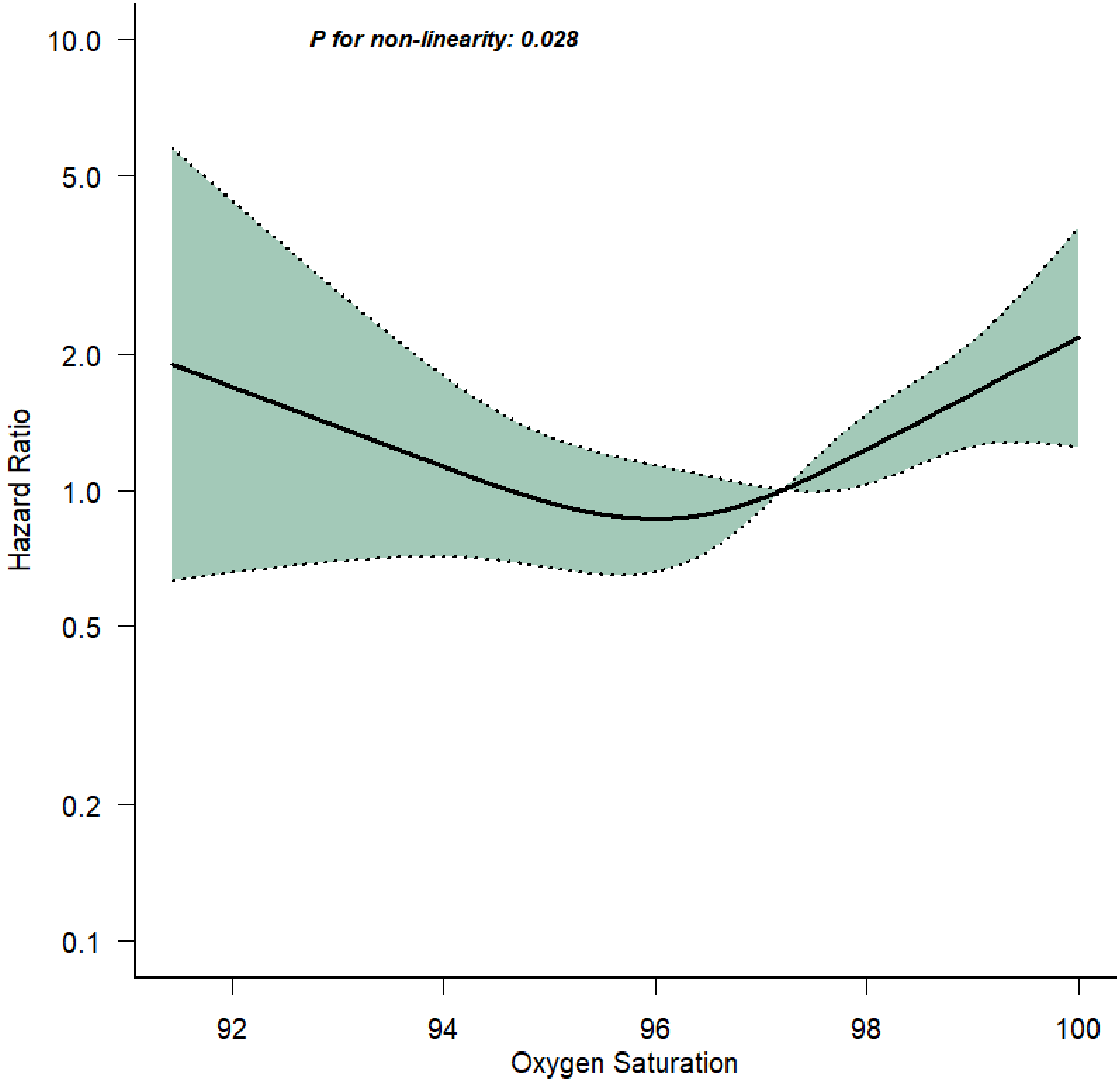
P for non-linearity: 0.028
10.0
5.0
2.0
1.0
0.5
0.2
0.1
Hazard Ratio
92
94
96
98
100
Oxygen Saturation

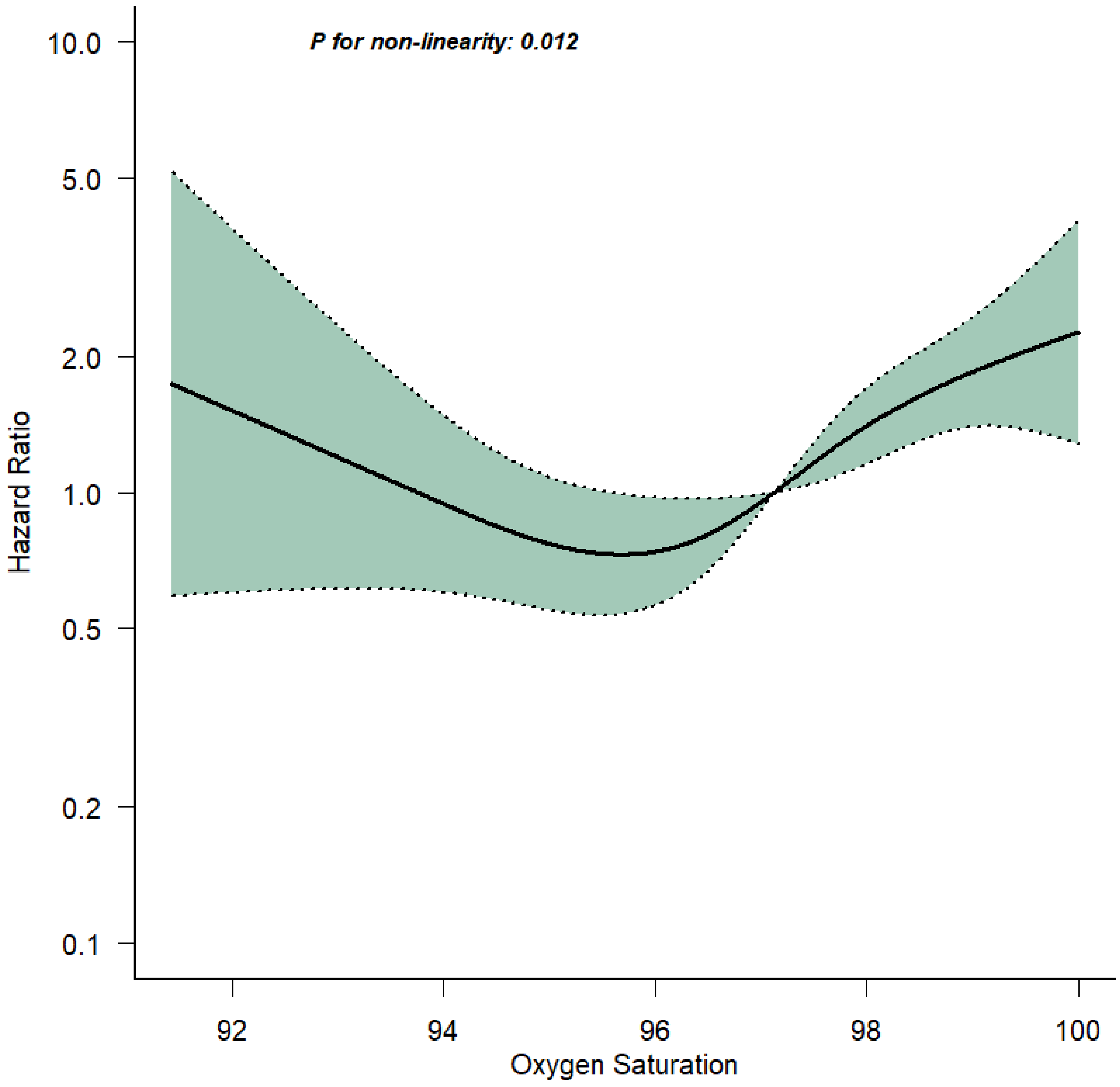
P for non-linearity: 0.012
10.0
5.0
2.0
1.0
0.5
0.2
0.1
Hazard Ratio
92
94
96
98
100
Oxygen Saturation

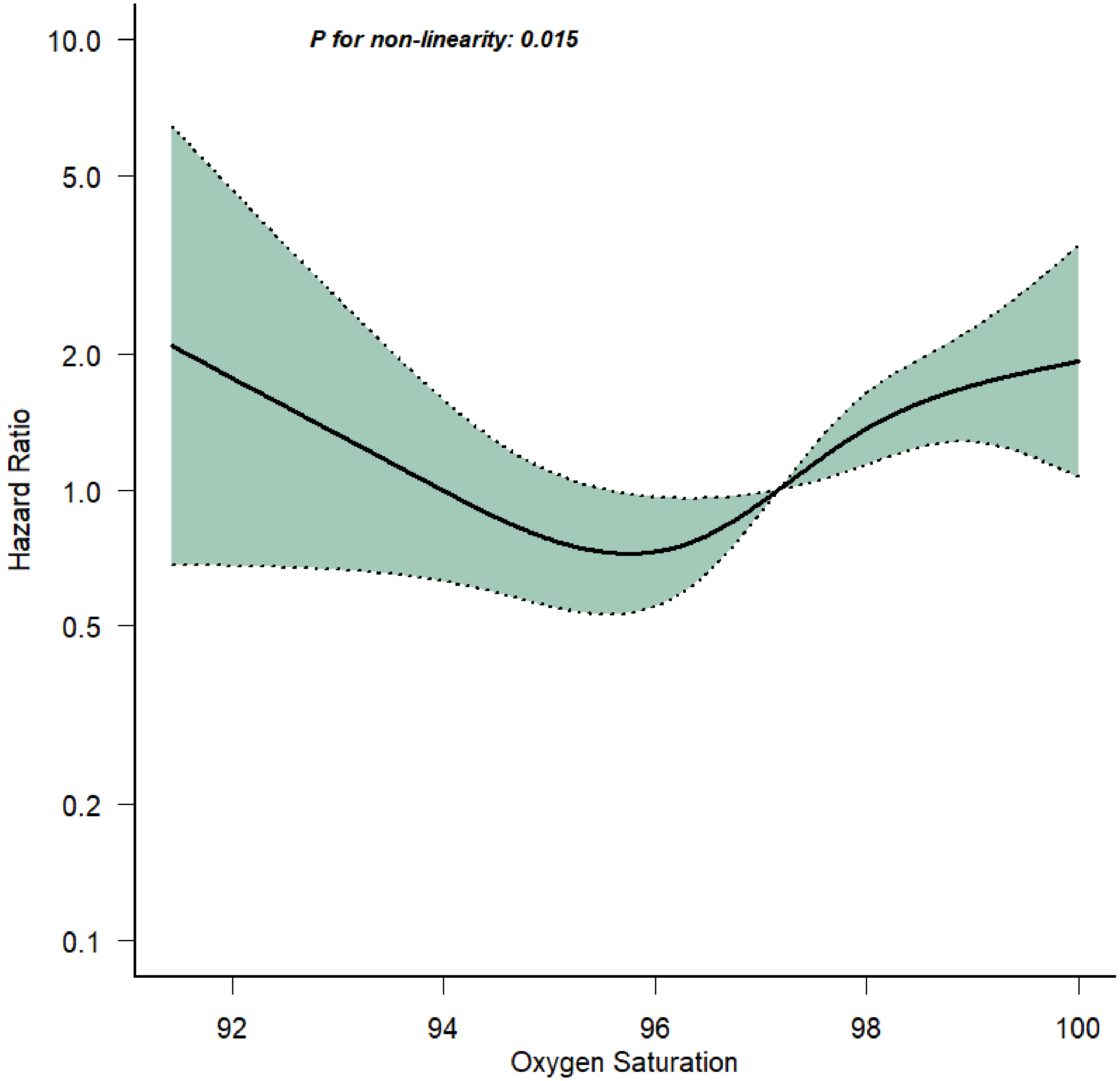
P for non-linearity: 0.015
10.0
5.0
2.0
1.0
0.5
0.2
0.1
Hazard Ratio
92
94
96
98
100
Oxygen Saturation

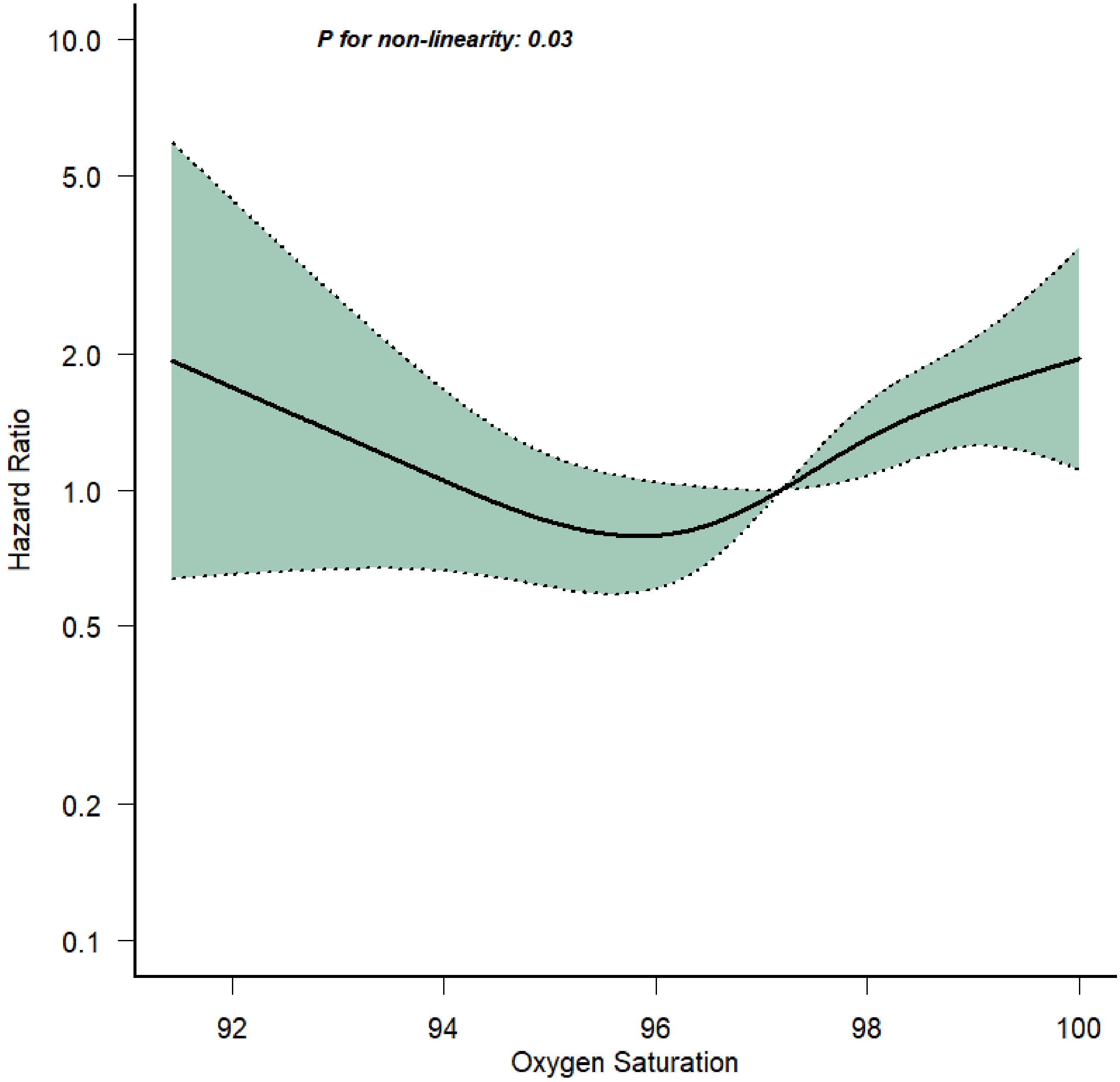

P for non-linearity: 0.03
Hazard Ratio
10.0
5.0
2.0
1.0
0.5
0.2
0.1
92
94
96
98
100
Oxygen Saturation
